# Eclipse Prediction on the Ancient Greek Astronomical Calculating Machine Known as the Antikythera Mechanism

**DOI:** 10.1371/journal.pone.0103275

**Published:** 2014-07-30

**Authors:** Tony Freeth

**Affiliations:** 1 Antikythera Mechanism Research Project, South Ealing, London, United Kingdom; 2 Images First Ltd, South Ealing, London, United Kingdom; Indiana University, United States of America

## Abstract

The ancient Greek astronomical calculating machine, known as the *Antikythera Mechanism*, predicted eclipses, based on the 223-lunar month *Saros cycle*. Eclipses are indicated on a four-turn spiral *Saros Dial* by *glyphs*, which describe type and time of eclipse and include alphabetical *index letters*, referring to *solar eclipse inscriptions*. These include *Index Letter Groups*, describing shared eclipse characteristics. The grouping and ordering of the index letters, the organization of the inscriptions and the eclipse times have previously been unsolved. A new reading and interpretation of data from the back plate of the Antikythera Mechanism, including the glyphs, the index letters and the eclipse inscriptions, has resulted in substantial changes to previously published work. Based on these new readings, two arithmetical models are presented here that explain the complete eclipse prediction scheme. The first model solves the glyph distribution, the grouping and anomalous ordering of the index letters and the structure of the inscriptions. It also implies the existence of lost *lunar eclipse inscriptions.* The second model closely matches the glyph times and explains the four-turn spiral of the Saros Dial. Together, these models imply a surprisingly early epoch for the Antikythera Mechanism. The ancient Greeks built a machine that can predict, for many years ahead, not only eclipses but also a remarkable array of their characteristics, such as *directions of obscuration*, *magnitude*, *colour*, *angular diameter of the Moon, relationship with the Moon’s node* and *eclipse time*. It was not entirely accurate, but it was an astonishing achievement for its era.

## Introduction

In the autumn of 2005, a major data gathering operation on the Antikythera Mechanism was carried out by an Anglo-Greek team of academics in collaboration with the National Archaeological Museum in Athens and two advanced technology companies [1].

**Figure 1:**
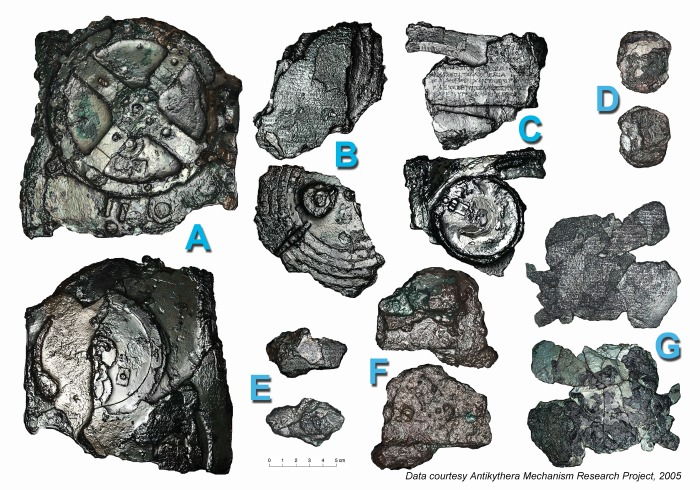
PTM of the seven lettered fragments A–G of the Antikythera Mechanism. The fragments of the Antikythera Mechanism as seen from both sides. In addition to the seven lettered fragments A–G, there are also seventy-five small fragments 1–75. The fragments are seen here using *Polynomial Texture Mapping* (PTM) [2], with specular enhancement, which emphasizes small surface details.

Figure 1 shows the surviving remains of the Antikythera Mechanism, which are now split into 82 fragments [1]. They are conserved in the National Archaeological Museum in Athens, Greece. Two new investigative techniques were used in 2005 on all the fragments of the Mechanism. *Polynomial Texture Mapping* (PTM) [2], now sometimes called *Reflectance Transformation Imaging* (RTI), is a technique for looking at fine surface details. *Microfocus X-ray Computed Tomography* (X-ray CT) [3] produces high-resolution 3D X-rays using a very small X-ray source. For details of these techniques, see Materials and Methods.

**Figure 2:**
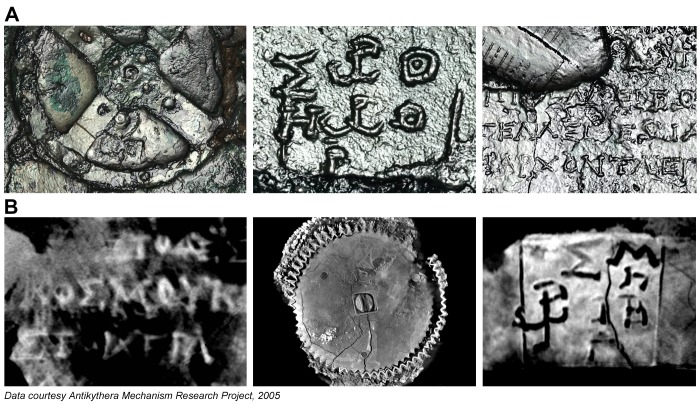
Examples of data gathered in 2005 on the Antikythera Mechanism. (A) Polynomial Texture Mapping (PTM) with specular enhancement [2]. (B) High-resolution Microfocus X-ray Computed Tomography (X-ray CT) [3].

Figure 2 shows PTM and X-ray CT data. The PTM data was expected to show new details of inscriptions on the surfaces of the fragments and the X-ray CT data to reveal the internal structure of the gearing. However, both techniques have contributed to many of the new discoveries about the structure, functions and inscriptions on the Antikythera Mechanism [1], [4], [5]. One surprising revelation was that the X-ray CT uncovered new scale divisions as well as several thousand new text characters, hidden within the fragments and entirely invisible either to visual inspection or the previous generation of 2D X-rays [6].

**Figure 3:**
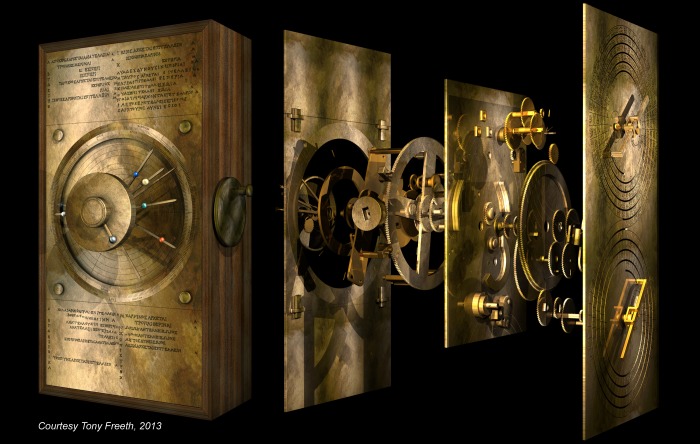
Exploded computer reconstruction of the Antikythera Mechanism. On the *left*, the front plate includes zodiac and calendar dials [6] and a conjectural reconstruction of the ancient Greek *Cosmos* [5]. In the middle is an exploded reconstruction of the gears. The input contrate gear is in the centre, with a keyway to turn the Mechanism. The planetary gearing at the front is conjectural [5], but the gearing behind the main plate for the lunar anomaly mechanism and the back dials is now firmly established [1], [6]. On the upper right is the 19-year Metonic calendar dial [1], [4], [7] and on the lower right, the 223-month Saros eclipse prediction dial [1].

The new data resulted in a sequence of major discoveries on the Antikythera Mechanism [1], [4], [5] with an underlying theme: its design was a highly ingenious fusion of ancient Babylonian and Greek mathematical astronomy. A computer reconstruction in Figure 3 shows the resulting instrument. In this research article, it is shown how mathematical concepts also underlie the eclipse prediction scheme on the Antikythera Mechanism.

**Figure 4:**
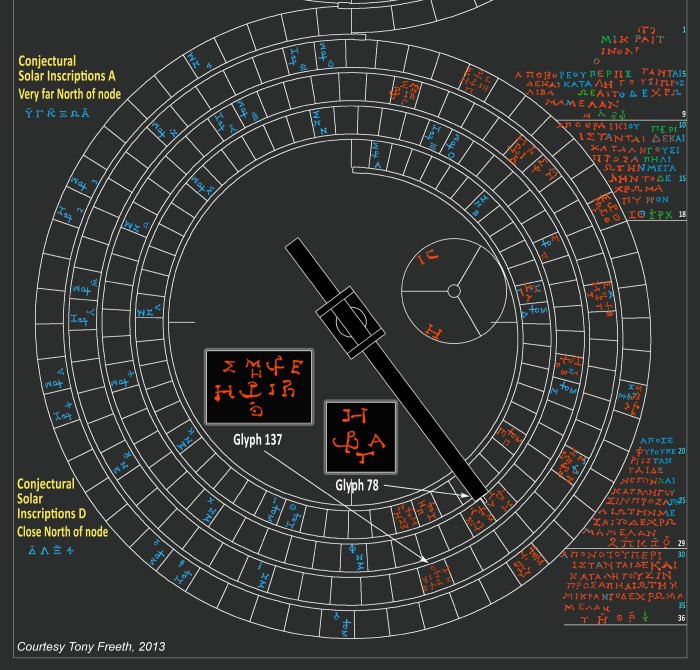
The 223-lunar month Saros Dial. Red text is traced from data; blue reconstructed from context; green is uncertain. Eclipse predictions–strictly speaking *predictions of eclipse possibilities* (EPs) [1]–are specified by *glyphs*, numbered by their month round the dial [4]: two examples are inset. Σ for ΣEΛΗΝΗ (*the goddess of the Moon*) indicates a lunar eclipse; Η for ΗΛΙΟΣ (*the god of the Sun*) a solar eclipse. In Glyph 137, Η under Μ, denoted by H^\M^, means ΗΜEΡΑΣ (*of the day*): a lunar eclipse during the day, which is therefore not visible. In other glyphs Ν under Υ, denoted by N^\Υ^, means NYKTOΣ (*of the night*): a solar eclipse during the night, which is therefore not visible. The eclipse time follows, with a ligature of ω and ρ, abbreviating ωρα (*hour*), followed by a letter for a number of hours [4]. At the bottom of the glyphs are *index letters* in alphabetic order, using two alphabets, plus three additional symbols. This alphabetic ordering previously established [4] that there were fewer solar than lunar eclipse predictions. The index letters in the glyphs reference inscriptions to the right of the dial, where the same index letters appear in groups, which are underlined in white and have white line numbers. These *Index Letter Groups* all reference *solar* eclipse inscriptions (Table S1). They are written in a perplexing non-alphabetic ordering. Conjectural inscriptions (in yellow) and conjectural *Index Letter Groups* (in blue) are predicted by the *Eclipse Year Model* (EYM). Inside the Saros Dial is the subsidiary Exeligmos Dial, which adds eight hours to the eclipse times for successive Saros periods [4].

The data that is important for this study all come from the lower half of the back plate of the Antikythera Mechanism, witnessed by fragments A, E and F (Figure 1, Figure S3). It was the scale divisions on the lower back dial, shown by these three fragments, which led to the discovery of eclipse prediction [1]. A new interpretation of the inscriptions on the lower half of the back plate of the Mechanism is given here. These are traced in Figure 4. A full analysis is given in Note S2.

Suppose a user of the Antikythera Mechanism wants to check eclipse predictions for a particular month some years ahead. The user winds the Mechanism forwards to the desired date, as shown on one of its calendars [1], [4], [7]. In Figure 4, the Mechanism’s gearing [1] has turned the Saros Dial pointer to Month 78, where Glyph 78 shows a predicted solar eclipse at the first hour after dawn. At the bottom of the glyph is an index letter, **T**. The user finds **T** in Line 36 at the bottom right of the Saros Dial: the first of five in this *Index Letter Group*. Above Line 36, six lines of inscription describe the characteristics of the eclipse: *directions*, *magnitude* and *colour* (Note S2). What are the organizing principles of the glyph distribution and the Index Letter Groups? Why are the index letters in a perplexing non-alphabetic order? What is the overall structure of the eclipse inscriptions? How were the eclipse times determined? Here it is shown that these long-unexplained issues can be solved by two arithmetic models with significant consequences.

## Materials and Methods

**Figure 5:**
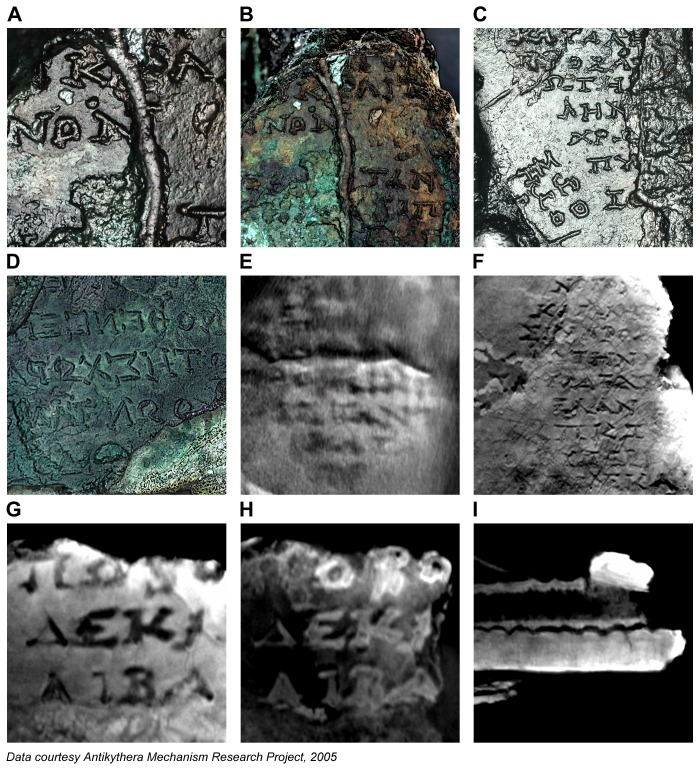
Inscriptions data from the back plate. (A) Fragment A, PTM of back plate with specular enhancement. (B) Fragment A, PTM of back plate with diffuse gain. (C) Fragment A, PTM of back plate with specular enhancement. (D) Fragment A, PTM of impression of back cover with luminance unsharp masking. (E) Fragment A, X-ray CT slice of back plate. (F) Fragment F, X-ray CT slice of back plate. (G) Fragment E, X-ray CT slice of back plate. (H) Fragment E, X-ray CT slice of accretion layer. (I) Fragment E, orthogonal X-ray CT slice of back plate and accretion layer.

This study is about the structure of eclipse prediction on the Antikythera Mechanism. Much of the relevant data comes from highly fragmentary inscriptions on the back plate, which are often very hard to decipher. Two techniques were used in the 2005 investigations [1]. PTM [2] combines many digital images, lit from different directions, with computer software (Figure 5 (**A**)–(**G**)). This gives the facility to interactively re-light a surface as well as the ability to factor out confusions of colour and texture to reveal essential surface details. A range of filters, such as *specular enhancement*, *diffuse gain* and *unsharp masking*, enable the data to be visualized for maximum character recognition. X-ray CT [3] projects images of the sample from many different angles onto an X-ray detector. These are then combined mathematically into a 3D X-ray volume. X-ray CT viewing software, for example, *VGStudio Max* (Volume Graphics), enables both 3D volumes as well as single “slices” at any angle through the volume to be isolated and analyzed.

To enable the reconstruction of the text shown in Figure 5, more than a hundred X-ray CT slices were exported as image stacks into *Photoshop* (Adobe) to enable the decipherment of the text. Together with PTMs, these enabled the surviving inscriptions to be traced using a digitizing tablet. The text characters are on average 1.6 mm high, with average line spacing of 2.5 mm. This is tiny text and the small size creates problems reading many of the characters, though it is remarkable how much has been preserved after 2,000 years under water. The quality of the X-ray CT data is variable between fragments. The X-ray technique involves projection of the sample from a microfocus X-ray source onto a 2D detector [3]. To fill the detector, the smaller fragments can be geometrically magnified to a greater degree than the larger fragments: so the resulting 3D X-ray volumes have inherently higher resolution. The resolution for Fragment E was 46 microns; for Fragment F 64 microns; and for Fragment A 101 microns (Scan 5). The highest resolution scan of Fragment A (Scan 6, 54 microns) was seriously compromised by a technical problem during data acquisition, whereby about 27 projections (out of 2,957) failed to record. There is also evidence that the fragment moved during the scan. Attempts to rectify these problems have only been partially successful. The difficulty with lack of resolution of the X-ray CT of Fragment A can be seen in Figure 5 (**E**). There have been considerable advances in X-ray CT technology since 2005, so it would be of great advantage to gather new X-ray CT data on the Antikythera Mechanism: there is much that cannot be read from the current data and X-ray CT has been developing rapidly in recent years.

Some characters are easy to read. For those that are not, many X-ray CT slices, just a few tens of microns apart, are often useful. A character sometimes appears to change as the slices are scrolled through–for example, from Λ to Δ to Α to part of Μ. It is often difficult to get a definitive interpretation, since many random marks often confuse the text.

Another aspect, which is sometimes helpful, is that much of the text is overlain with an *accretion layer* that also includes text information. The text was engraved into bronze: the accretion layer must have built up gradually on the surface, moulding itself to the form of the text letters and finally concreting into a hard deposit over time [1]. This has created a cast of the original engraved surface. The effect of the accretion layer on scrolling through X-ray CT slices is illustrated in Figure 5 (**G**) and (**H**). The text characters first appear as black on grey, Figure 5 (**G**)–black showing where the engraving tool has removed the metal, so there is an absence of X-ray density; then as white or light grey on dark grey, Figure 5 (**H**), where the X-ray CT slice intersects the cast of the same text characters in the accretion layer. In places the accretion layer has survived better than the original engraving. The advantage can be seen in reading the third character in the top row of the text: in the direct engraving in Figure 5 (**G**) this character is hard to read; in the accretion layer image in Figure 5 (**H**) it is evidently Β. In many cases the accretion layer has become detached and slightly displaced from its original position, as seen in Figure 5 (**I**). In the case of the back cover inscription, Figure 5 (**D**), most of the original text has been lost and all that is left is the accretion layer, which was deposited onto Fragments A and B and only survives as mirror text on their surfaces [1], [6].

The Antikythera Mechanism is conserved in the National Archaeological Museum in Athens, Greece (http://www.namuseum.gr/collections/bronze/ellinistiki/ellinistiki06-en.html; Accession Number X 15087). Full data from the 2005 investigations can be accessed by application to the Antikythera Mechanism Research Project (http://www.antikythera-mechanism.gr/). *All necessary permits for these investigations were obtained from the Central Archaeological Council in Greece.*

## Results

### Glyph distribution

Basic properties and definitions concerning eclipses as well as the underlying cyclic parameters of the Antikythera Mechanism can be found in Note S1. Previous research proposed a mathematical model for the distribution of the glyphs round the Saros Dial, which was consistent with the alphabetic index letters [4]. However, this model could not have been easily calculated in ancient Greece and it needed to be calibrated from a lunar eclipse 210 lunar months (nearly 17 years) earlier: so it was not really plausible. The first model presented here does not suffer from these problems. Like the earlier model, it uses principles of *closeness to node* and the *asymmetry of observability of solar eclipses* (Note S1), but it is much simpler to calculate. It solves the distribution of the glyphs, the Index Letter Groups and the structure of the inscriptions. A key concept is the *eclipse year* (Note S1), so the first model will be called the *Eclipse Year Model*, EYM.

**Figure 6:**
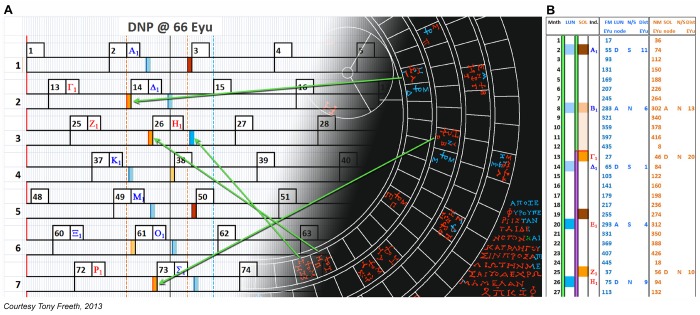
Detail of spreadsheets showing EYM. (A) Part of a spreadsheet (shown in full in Figure S9) illustrating how EYM maps syzygies onto eclipse years. EYM is based on mean months. Each month is divided into 38 EYu. The 19 *eclipse years* are numbered on the left, with 446 EYu in each eclipse year. Eclipses are clustered in *eclipse seasons* around the *node points*: here at the *Descending Node Point* (DNP) (Note S1). Observed glyphs are in bright blue for lunar and bright orange for solar; glyphs reconstructed by EYM in paler colours; observed index letters in red; index letters reconstructed by EYM in blue. The two alphabets of index letters are distinguished here by subscripts, though the original index letters were distinguished by bars on the second alphabet. The superimposed Saros Dial shows how glyphs are mapped onto eclipse years. The grey vertical line is the DNP at 66 EYu from the Saros start. The blue dotted line is one side of the symmetrical limits for lunar glyphs; the orange dotted lines are the asymmetrical limits for solar glyphs as well as the lunar limit North of the node, as described in the text. Months start at *First Crescent Moon* (Note S1), 2 EYu (1.55 days) after New Moon. Full Moon is at 17 EYu from the start of each Month; and New Moon at 36 EYu. (B) Part of a spreadsheet (shown in full in Figure S10), which calculates the glyphs generated by EYM, with *index letter*; *EYu from eclipse year start*; *Descending or Ascending node*; *North or South of the node*; *EYu from node point*.

In each Saros period, there are 223 lunar months and 19 eclipse years (Note S1). In Figure 6 and Figure S9, each eclipse year is lined up exactly below the preceding one. To accomplish this, each lunar month is divided into 38 units, called *Eclipse Year units* (EYu), each representing 0.78 days. There is a precedent in dividing the synodic month into artificial units in the Babylonian division of the month into 30 *tithis* [9]. However, the author has found no classical sources with evidence that EYu were used in ancient times. One source describes ancient Chinese lunar astronomy by Liu Hong in c. 200 AD [10], which used a table with units that are 1/19^th^ of a *du,* which is a Chinese degree, where there are 365.25 *du* in a full circle. The following theory could equally well be expressed in terms of the number of days or degrees that the Moon is from its nodes, without any reference to EYu. However, this approach would carry an unnecessary constant in all the calculations, both complicating the arithmetic and obscuring the very economic and coherent basis for the theory.

The definition of EYu means that each eclipse year has 446 EYu and the distance between the node points is 223 EYu. EYM is defined entirely using integers. In Figure 6, the positions of the n^th^ Full Moon (FM_n_) and n^th^ New Moon (NM_n_) in EYu from the start of the eclipse year are 38(n-1)+17 (mod 446) and 38(n-1)+36 (mod 446)–both easy to calculate for the ancient Greeks. A key issue is the positions of the *node points* (Note S1). The observed glyphs give initial estimates, with lunar glyphs a better guide than solar glyphs, since they are not affected by *asymmetry* (Note S1): the average position at the first node is 64.2 EYu and at the second is 289.7 EYu (Table S2). For EYM, the difference should be 223 EYu, so these must be modified. Trial-and-error finally determined 66 EYu and 289 EYu as the figures that generate all the data. EYM creates a glyph if it is sufficiently close to the nearest node point in EYu, within the following limits:

**A lunar glyph:** *if Full Moon is ≤* ***20*** *EYu from the node point*.

**A solar glyph:** *if New Moon is ≤* ***20*** *EYu from the node point, if* ***North*** *of the node.*

***≤7*** *EYu from the node point, if* ***South*** *of the node*.

These limits were determined by trial-and-error so that the model fits the data. To decide whether the first node point is *Ascending* or *Descending*, the asymmetry of the solar glyphs is exploited. By calculation, Glyph 13 is 46 EYu from the start of the eclipse year, in other words, 20 EYu before the node point at 66 EYu. So it must be North of the node: otherwise it would have been excluded by the asymmetrical criterion for solar glyphs. So the first node point must be the *Descending Node Point* (DNP) (Note S1). *Closeness to node* is not the same as *Closeness to node point*, since the node point is defined when the Sun is at the Moon’s node, not when the Sun is at a nearby eclipse. However, these two concepts are related by a constant multiple of ^235^/223 (Note S3). So EYM is defined by *Closeness to node point* to preserve simple integer calculations. One additional factor must be added to EYM: the *consecutive month rule* excludes a second lunar eclipse prediction in the same month. Such eclipses are nearly always penumbral and were never included in Babylonian eclipse prediction schemes [8].

EYM generates glyphs, which match the alphabetical index letters (Figure 6, Figure S10). There are 51 glyphs, with 38 predictions of lunar eclipses and 28 predictions of solar eclipses. It only differs from a previous model [4] in that Glyph 149 is Σ, Η in EYM, whereas it is Σ-only in the old model. The pattern of lunar glyphs generated by EYM conforms to the 8-7-8-7-8- pattern of Babylonian eclipse prediction schemes [8] (Note S1). Surprisingly the solar glyphs are a subset of the non-Babylonian pattern 8-8-8-7-7-, a feature shared by the old model [4], though not apparently noticed when this was published.

### Index Letter Groups

There are four surviving Index Letter Groups (Figure 4). Here they are considerably revised and augmented from previously published versions: full details of their Interpretation from the data are in Figure S6. In the following, each group is preceded by Its line number; BOLD is traced from the data, REGULAR is reconstructed from the context and ITALICS is uncertain.

**L. 9: Ν_1_**, *Λ_2_*, *Β_1_*, *Φ_2_*

**L. 18: Ζ_1_**, Θ_1_, *Σ_2_*, *Ρ_1_*, *Χ_1_*

**L. 29: 2, Π_2_**, **Κ_1_**, **Ζ** *_2_*, **Φ_1_**

**L. 36: Τ_1_**, **Η_2_**, **Θ_1_**, **Ρ_2_**, ***Ψ*** *_2_*

All the index letters in these groups refer to glyphs that include a solar eclipse prediction; less than half also include a lunar eclipse prediction (Table S1). So these Index Letter Groups refer to solar eclipses and the corresponding lines of inscription describe shared characteristics of solar eclipses. The grouping of the index letters and their ordering within each group have been long-term unsolved problems. Here it is shown how a solution is provided by EYM. The essential idea is to calculate each eclipse prediction’s distance in EYu North or South of the node point and then list the North eclipses followed by the South eclipses in descending distance order.

**Figure 7:**
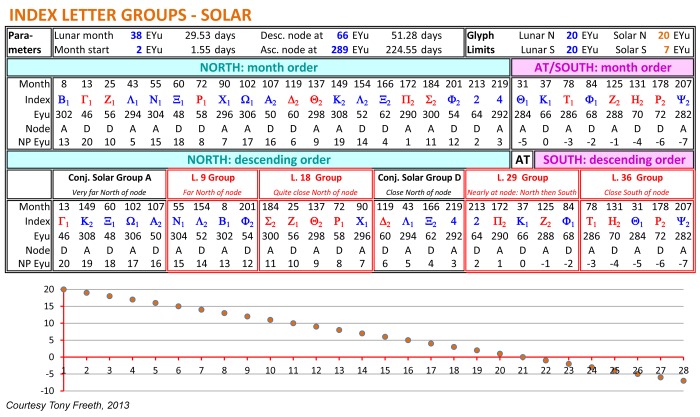
Generation by EYM of the Index Letter Groups. EYM’s predictions *North of the node* are first arranged in *month order*, including: *month number*, *index letter*, *EYu from eclipse year start*, *Ascending or Descending node* and *EYu from node point* (NP EYu). Similarly for predictions *At the node/South of the node*, with a negative sign attached to their NP EYu to match their negative *ecliptic latitude* (Note S1). They are then re-ordered by *EYu from node point* in *descending order*. This generates the observed Index Letter Groups (in red) and the ordering of the letters within each group (with one exception). It also shows how EYM completes the picture with two conjectural solar Index Letter Groups (in black).

As shown in Figure 7, EYM reconstructs the observed Index Letter Groups and implies the existence of two further lost Index Letter Groups. EYM establishes that the eclipse inscriptions were inscribed round the dial in descending order from their furthest distance North of the node to their furthest distance South. It is striking that the re-ordered EPs alternate between Ascending and Descending node and the NP EYu figures form an exactly linear ordering. The underlying reason for these patterns is that the positions of the New Moons in EYu within the eclipse year form a complete set of odd numbers from 1–445 (with no two being equal), because of their mathematical definition (Note S3).

EYM has a flaw: Σ_2_ is in the right L.18 Group but in the wrong place within the group. Σ_2_ is the first letter according to EYM but the evidence shows that it is not the first and is almost certainly the third letter, between Θ_2_ and Ρ_1_ (Figure S6). All the rest of the data fit exactly with EYM, so this is surely evidence of a mistake. *Closeness to node point* can be regarded as a surrogate measure for *ecliptic latitude* (Note S3). So an Index Letter Group defined by EYM is essentially a band of ecliptic latitude, analogous to a *clima* in ancient geography–with the frame of reference being the ecliptic plane, not the equator. To understand the eclipse characteristics grouped by the Index Letters, it is necessary to decipher the inscriptions round the Saros Dial.

### The eclipse inscriptions

A full epigraphic interpretation and analysis of the inscriptions by Dr Charles Crowther (Oxford University) is included in Note S2. The following is based on his interpretation of the data.

**Figure 8:**
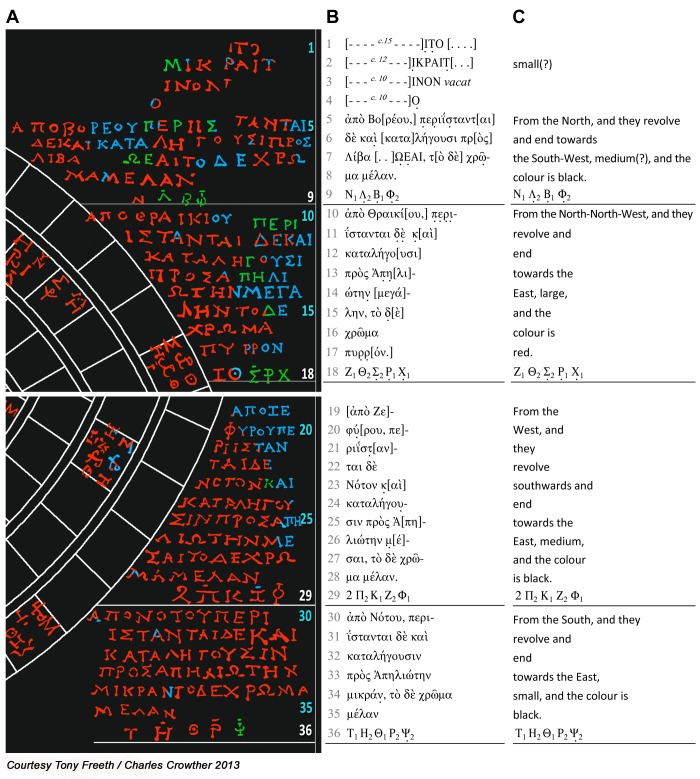
The solar eclipse inscriptions. (A) Text that is traced from the data is in red; text reconstructed from the context in blue; uncertain text in green. The Index Letter Groups, underlined in white and with white line numbers, refer to the lines of inscription above them. (B) Transcription using Leiden conventions. (C) Translation.

The inscriptions, which are traced and interpreted in Figure 8, have a repetitive pattern, with *directions*, *magnitude* and *colour* for each group. It was argued in a publication of 1974 [6] that the *directions* refer to winds, though it was not understood at that early stage that the dial predicted eclipses. This idea has persisted, though it is argued here that they must refer not to winds but to *directions of obscuration* of eclipses (Note S2). It must be said that the whole scheme does not work well for solar eclipses: for a total solar eclipse, both *directions of obscuration* and *magnitude* are critically dependent not only on the Moon’s ecliptic latitude but also on the location of the observer relative to the path of totality. *Colour* is rarely observed or recorded for solar eclipses in ancient or modern astronomy. The system works much better for lunar eclipses, since their visibility and characteristics are not dependent on the location of the observer. The same mathematical exercise that derived the *solar* Index Letter Groups can also generate *lunar* Index Letter Groups (Figure S11): the difference being that they are symmetrical relative to North and South (Note S3). All the observed glyphs have index letters, yet twenty-one of these glyphs are lunar-only. There was no point in indexing these if there were no associated *lunar eclipse inscriptions*: nearly a whole alphabet of index letters could have been saved. This argument alone is enough to establish the strong likelihood of lunar Index Letter Groups and associated inscriptions. All the characteristics work better for lunar eclipses: ecliptic latitude is a reasonable indicator of both directions of obscuration and magnitude (Note S2); lunar eclipse colour was often recorded in antiquity (Note S2). It appears that the lunar eclipse inscriptions were conceived first and the dysfunctional solar eclipse inscriptions added for completeness. Plausible inscriptions could be reconstructed for all the conjectural lunar Index Letter Groups.

**Figure 9:**
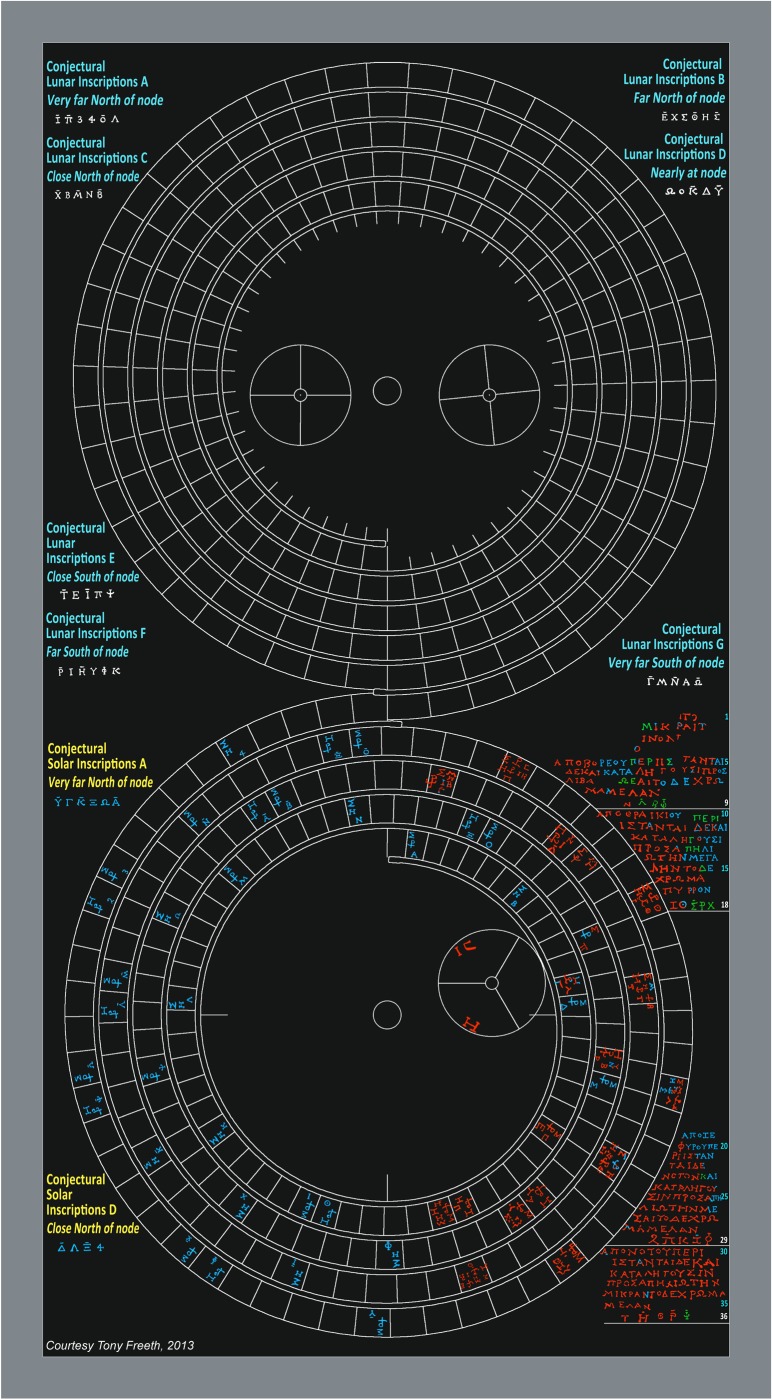
Conjectural arrangement of back plate inscriptions. This uses the same colour conventions as described in the legend for Figure 9.

With no room round the Saros Dial, the only possible place for lunar eclipse inscriptions is round the Metonic Dial, where the plate has only survived for a few millimetres beyond the dial itself: so the direct evidence is lost. Figure 9 shows how they fit neatly here. The geometry of the spiral dials means that there is more room on the left of the Metonic Dial than on the right, so the reconstruction includes four Index Letter groups on the left and three on the right.

All the evidence [1], [4], [5] suggests that the designer of the Antikythera Mechanism wanted to create a complete machine–an astronomical compendium that would answer all predictive questions within the scope of the astronomy of the time. Though there is no direct evidence, the arguments that the Antikythera Mechanism also included lunar eclipse inscriptions are compelling. They would also provide a satisfying mathematical completeness to this instrument of mathematical astronomy.

### Predicted eclipse times

Each glyph on the Antikythera Mechanism includes a predicted eclipse time in hours. A previous paper [4] concluded that, *“… the process of generation of glyph times was not sound and may remain obscure.”* Counteracting this pessimism, a model is described here, derived from Babylonian *System B* [9], [11], which closely matches the glyph times. A re-examination of previous readings of the data [4] resulted in crucial modifications to several glyph times (Figure S14, Note S4, Table S3). The essential difficulty of modelling the eclipse times is as follows: if the time of FM_1_ is given, calculating the time of FM_n_ involves adding the sum of all previous variable month lengths (Figure S15). After many unproductive attempts with epicyclic models [1] and Babylonian *System B* models based on daily increments [9], [11], [12], another type of System B model was found to be successful (Note S4). This calculates the synodic month length much more simply from zigzag functions, dependent only on the phases of the lunar and solar anomalies at the end of each month [9], [11]. The Babylonian data is uncertain: the solar contribution to month length appears to have been calculated from second-order differences, generating piecewise-parabolic arcs [11], [13] (Note S4). Since these have not proved to be more successful than linear zigzags, the *ZigZag Model* (ZZM) described here uses only linear zigzags. There are two types of variable input parameter: *parameters tied to the astronomy* are the *minima* and *maxima* of the zigzag functions, which define the month lengths and can only be altered slightly before a good fit with actual month lengths breaks down (Figure S15); *free parameters* are the *phases of the lunar and solar anomalies* at FM_1_ and the *input times* of FM_1_ and NM_1_, which can be chosen freely to optimize ZZM.

**Figure 10:**
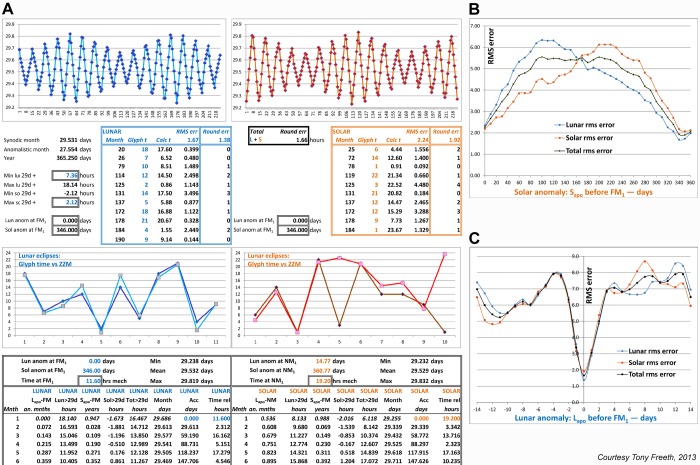
The ZigZag model, ZZM. (A) Detail of spreadsheet (described in Note S4), showing the first few rows of calculation out of 223, with parameters optimized and final errors rounded to whole numbers. The graphs at the top show the generated month lengths and the graphs in the middle show the close match of the model with the glyph times, with a lunar rms error of 1.4 hours and a solar rms error of 1.9 hours, giving a total rms error of 1.7 hours. The apparently large errors in the fifth and tenth solar times are much smaller than they seem, since the *clock distance* error is the relevant measure (Note S4). (B) Optimization of the solar anomaly parameter: rms error of model times vs glyph times, dependent on solar anomaly, with lunar anomaly fixed when *lunar apogee*, L_apo_ = FM_1_. Optimal value is 346 days before *solar apogee*, S_apo_. (C) Optimization of the lunar anomaly parameter: rms error of model times vs glyph times, dependent on lunar anomaly, with solar anomaly fixed at 346 days before S_apo_. Optimal value is at zero when L_apo_ = FM_1_.

Figure 10. shows the results of optimizing the free input parameters. The optimal value of the lunar anomaly occurs at a sharp minimum where the *lunar apogee*, L_apo_ = FM_1_ (Figure 10 (**B**)), strongly supporting a previous proposal [4] that each quadrant of the Saros Dial was synchronized with the *Full Moon Cycle* (Note S5): at each eclipse, the position of the Saros pointer within each quadrant tells the user the angular diameter of Full Moon, which is at a minimum at the start of the quadrant and reaches a maximum in the middle. The inverse is true for New Moons (Note S5). A central solar eclipse should be total if it is predicted by a glyph near the cardinal points of the Saros Dial and annular near the intercardinal points, with hybrid eclipses between these regions. Figure S21 (**C**) shows that a “matching” sequence (Figure S19) of actual solar eclipses [14] strongly supports this design.

EYM and ZZM put constraints on the possible start dates for the Saros Dial. EYM establishes the DNP as 66 EYu after the dial start and ZZM adds the condition that L_apo_ = FM_1_. Calculated ephemerides [14] determine that there are only eight matching start dates from 250 BC to 1 BC (Table S5), a period that almost certainly includes the epoch of the Mechanism (Note S5). Also optimizing ZZM is the solar anomaly at 346 days before FM_1_. This equates to the ecliptic longitude of the mean Sun at FM_1_ being 46.53° (Note S5). The only matching date is FM_1_ at-204 May-12 (Table S5), when the mean Sun was at 46.75°: all the other dates are more than 10° wrong, reflecting an underlying flaw in the glyph time system (Table S4). This surprisingly early date for the Saros Dial has been suggested previously, using conflicting methods (Evans, J., Carman, C. C. *On the Epoch of the Antikythera Mechanism*, Workshop presentation, Leiden, 2013.) The result here should be treated with due caution, since ZZM is not an exact model, though all variants of the model considered to date share the same optimizing parameters. Further modifications may eventually lead to an exact model but the key input parameters are not expected to change. In addition, it should not necessarily be inferred that the date of the Antikythera Mechanism is the same as the date for which the Saros Dial was designed. The Mechanism could, for example, have been made at a later date. The new epigraphic analysis of the eclipse inscriptions presented here (Note S2) is fully consistent with 205 BC, though there is still debate about the relevance of some comparanda from the Athenian palaeographical tradition from the Hellenistic period (Note S2).

To make predictions, ZZM must be synchronized with both a lunar and a solar eclipse before the start of the dial (Note S5). Suitable eclipses are the total lunar eclipse of -207 Feb-16 19∶14 UT and the partial solar eclipse of -206 Jul-17 15∶50 UT [14], as established in Note S5 and shown in Table S6 (**A**). Whether or not these give a match to the data depends on the local time of their observation: in other words the longitude of the Mechanism’s intended use. A good candidate region for this is Epiros in Northwestern Greece [4], [16] (UT+1.3 hours). For this region, the errors in these eclipse times for optimizing ZZM are only −16 minutes for the lunar eclipse and +5 minutes for the solar eclipse, as described in Note S5 and shown in Table S6 (**B**).

## Conclusions

An epoch for the Antikythera Mechanism in 205 BC brings it close to the life of Archimedes, who was killed in the siege of Syracuse in Sicily in 212 BC. It is known from the writings of Cicero that Archimedes built a machine just like the Antikythera Mechanism [6]:

*“… the famous Sicilian had been endowed with greater genius than one would imagine it possible for a human being to possess… this… globe… on which were delineated the motions of the sun and moon and of those five stars which are called wanderers… (the five planets)… Archimedes… had thought out a way to represent accurately by a single device for turning the globe those various and divergent movements with their different rates of speed…”*

Cicero, De re publica, 54–51 BC

It also brings the Antikythera Mechanism close to Apollonios of Perga, who died in about 190 BC. He initiated the epicyclic theories [15] on which the lunar and (very likely) the planetary mechanisms were based [5]. It would be purely speculative to suggest that the Antikythera Mechanism owed its design to the greatest mathematician and scientist from ancient times, Archimedes, in collaboration with one of the greatest mathematicians and geometers, Apollonios of Perga. The historical record is so fragmentary that it could have been made by an unknown genius, with knowledge of the mathematical astronomy of the era, who made one of the greatest technological advances of all time, yet has left no known trace on history–except the Antikythera Mechanism!

The author has found it very productive to view the Antikythera Mechanism from his own academic background as a mathematician. Though subjective, this perspective, emphasizing the idea that the Antikythera Mechanism was essentially a mathematician’s instrument, has proved very successful in discovering its structure and functions. Its Earth-Sun-Moon system has a brilliant design, based on two great arithmetic cycles from ancient Babylon and the beautiful geometric theory of lunar motion from ancient Greece [1]. The mechanism that calculates the lunar phases is an exquisite and economic differential design [17]. The likely incorporation of the planets into the Antikythera Mechanism was almost certainly based on arithmetic period relations from Babylon and virtuoso epicyclic mechanisms to follow variable motions, just like the lunar anomaly mechanism [5]. The design of the upper Metonic calendar dial, with its five-turn spiral of 235 lunar months and 110 *excluded days*, is a highly ingenious concept [4].

**Figure 11:**
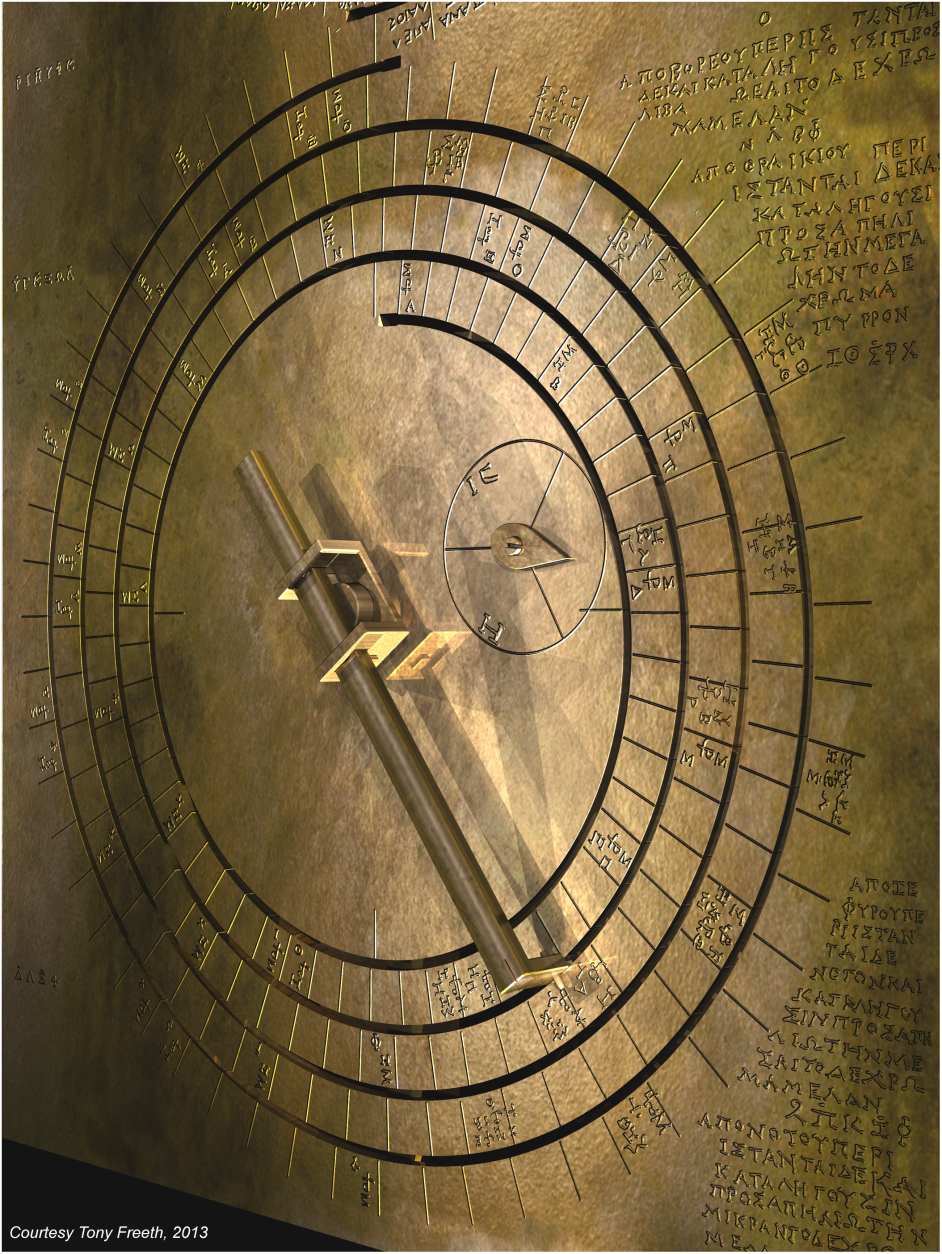
Computer reconstruction of the Saros and Exeligmos Dials.

Figure 11 shows a computer reconstruction of the Saros and Exeligmos Dials and associated solar eclipse inscriptions. The mathematical basis of the Antikythera Mechanism is further underlined by this research article, with its eclipse prediction scheme based on the four-turn geometry of the Saros Dial and synchronized with the Full Moon Cycle. It was driven by the Saros cycle–a surprising arithmetic resonance between three orbital periods of the Moon. It was designed using whole number arithmetic, which was highly regarded in ancient Greece [18] as well as the remarkable arithmetic prediction schemes of ancient Babylon [8], [9], [11]. The Antikythera Mechanism was an inspired synthesis of arithmetic and geometry as well as of Babylonian and Greek scientific cultures. It was a brilliant mathematician’s creation.

The main text is enhanced with notes, some of which include supplementary references. Note S2 includes additional references [19], [20], [21], [22], [23], [24], [25]. Note S3 includes additional references [26], [27], [28]. Note S4 includes additional references [29], [30], [31], [32], [33], [34], [35], [36], [37].

## Supporting Information

Figure S1 **The eclipse paths of nine solar Saros repeats.**
(PDF)

Figure S2 **Total solar eclipse diagrams.**
(PDF)

Figure S3 **The Saros Dial, superimposed on X-ray data of Fragments A, E and F.**
(PDF)

Figure S4 **The published inscriptions round the Saros Dial.**
(PDF)

Figure S5 **Tracing of the back plate inscriptions.**
(PDF)

Figure S6 **Data and interpretation for Index Letter Groups.**
(PDF)

Figure S7 **Comparative inscription on stone from Hellenistic Corinth.**
(PDF)

Figure S8 **Possible definitions of directions of obscuration.**
(PDF)

Figure S9 **The Saros Dial divided into eclipse years.**
(PDF)

Figure S10 **The glyphs that are automatically generated by EYM.**
(PDF)

Figure S11 **Spreadsheet generating the lunar Index Letter Groups according to EYM.**
(PDF)

Figure S12 **Graphic of Total lunar eclipse of 2011 Jun-15.**
(PDF)

Figure S13 **Glyph data and its interpretation.**
(PDF)

Figure S14 **Data for questioned glyph times.**
(PDF)

Figure S15 **Actual month lengths vs ZZM month lengths.**
(PDF)

Figure S16 **Graphics showing ZZM.**
(PDF)

Figure S17 **Excel spreadsheet that calculates ZZM with arbitrary input parameters.**
(PDF)

Figure S18 **Lunar eclipses for matching sequence beginning-04 May-12.**
(PDF)

Figure S19 **Solar eclipse paths for matching sequence beginning-204 May-12.**
(PDF)

Figure S20 **Theoretical EYu vs Actual gamma for sequence starting-204 May-12.**
(PDF)

Figure S21 **The Saros Dial and the Full Moon Cycle.**
(PDF)

Tables S1 **The observed index letter groups and corresponding glyphs.**
(PDF)

Tables S2 **The positions of the observed glyphs on the Saros Dial.**
(PDF)

Tables S3 **Eclipse times in the glyphs.**
(PDF)

Tables S4 **Comparison between eclipse times one Saros apart.**
(PDF)

Tables S5 **Astronomical parameters at possible start dates for the Saros Dial.**
(PDF)

Tables S6 **Possible synchronizing eclipses and times.**
(PDF)

Note S1 **Eclipses & Predictions.**
(PDF)

Note S2 **Eclipse Inscriptions.**
(PDF)

Note S3 **Eclipse Year Model, EYM.**
(PDF)

Note S4 **Glyph Times.**
(PDF)

Note S5 **Astronomy & Epoch.**
(PDF)
