# Supplementary material for: Eclipse Prediction on the Ancient Greek Astronomical Calculating Machine Known as the Antikythera Mechanism
Source: PLoS One. 2014 Jul 30;9(7):e103275. doi: 10.1371/journal.pone.0103275 (PMC4116162; doi:10.1371/journal.pone.0103275)
Supplement: Figure S1 — The eclipse paths of nine solar Saros repeats. (PDF) [file pone.0103275.s001.pdf]

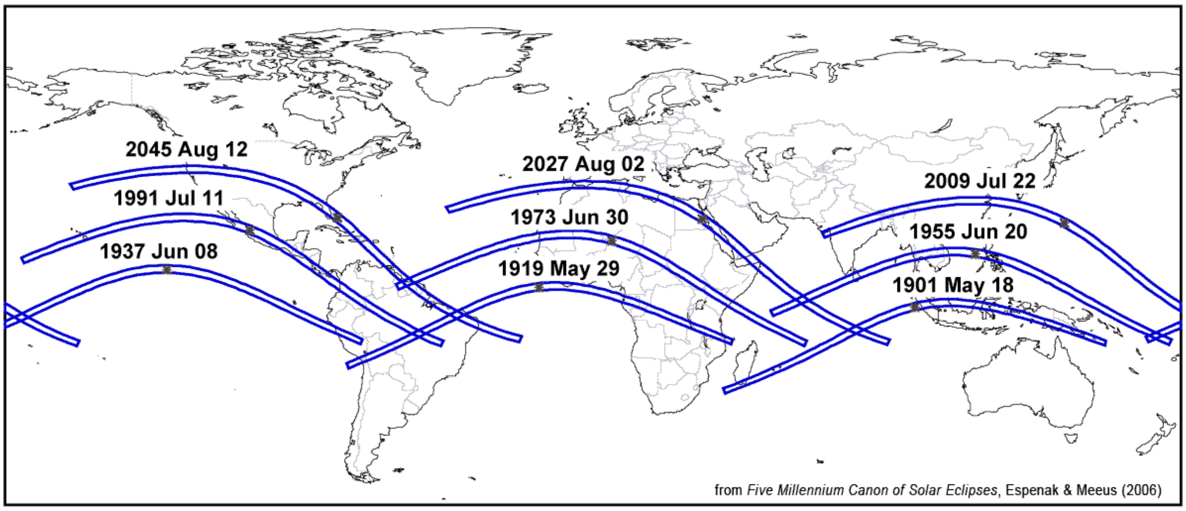

Eclipse map/figure/table/predictions courtesy of Fred Espenak, NASA/Goddard Space Flight Center

Figure S1 | The eclipse paths of nine solar Saros repeats [14]**.** The Saros period is approximately 6,585⅓ days [14]. The ⅓ day means that the Saros repeat is moved about 120° around the world and happens about 8 hours later. After three Saros periods, the eclipse repeat returns to almost the same longitude and time. This triple Saros cycle is known as the *Exeligmos cycle* (meaning *"Turn of the Wheel"*) and it is represented on the Mechanism by the subsidiary *Exeligmos Dial* [1]. Remarkably, the Saros repeat continues for 12 - 15 centuries. During this time the sequence starts as a small partial eclipse, visible near one of the poles. It then spirals up or down the globe, with about 70 central eclipses in the middle of its life, before spiralling to the opposite pole [14]. The figure shows the central part of the life of such a Saros series of repeats. The fact that the Saros period is also close to a whole number of anomalistic months means that the type of eclipse will also repeat, since the angular diameter of the Moon at the eclipse is also repeated. Both parts of the Saros equation are used in the design of the Saros Dial.
