# Supplementary material for: Eclipse Prediction on the Ancient Greek Astronomical Calculating Machine Known as the Antikythera Mechanism
Source: PLoS One. 2014 Jul 30;9(7):e103275. doi: 10.1371/journal.pone.0103275 (PMC4116162; doi:10.1371/journal.pone.0103275)
Supplement: Figure S2 — Total solar eclipse diagrams. (PDF) [file pone.0103275.s002.pdf]

A **B**

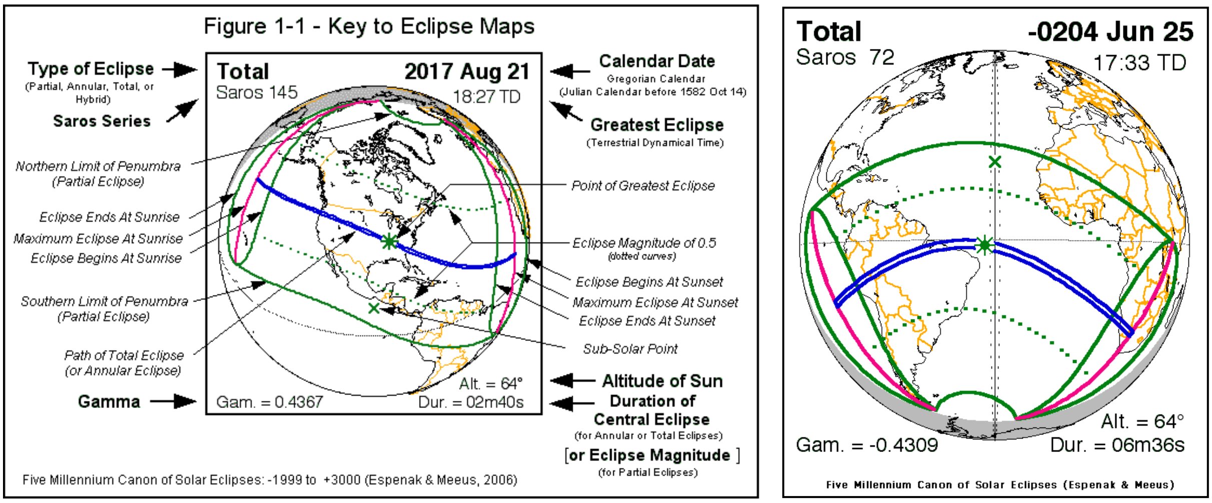

Eclipse map/figure/table/predictions courtesy of Fred Espenak, NASA/Goddard Space Flight Center

Figure S2 | Total solar eclipse diagrams [14]. (**A**) This eclipse is just North of the node (gamma = 0.44). The key explains the features of the eclipse. The blue lines show the passage of the umbral shadow, which is the *path of totality*. It is typically about 160 km in diameter, travelling from West to across the Earth's surface at about 1,700 km/hour. The green lines represent the limits of the penumbral shadow, where the eclipse appears to be partial. This is simply the effect of *parallax*, whereby a close object (the Moon) changes its relationship with a distant object (the Sun), when the observer moves position. (**B**) The date of the eclipse is given in the customary way using negative numbers, so that -204 refers to 205 BC. This is a total solar eclipse, which was too far South of the node to be visible (gamma = -0.43), even as a partial eclipse, from anywhere in the ancient Greek empire, as the upper green line shows. This parallax effect was recognized by the second century AD Greek astronomer, Claudius Ptolemy, who calculated limits for the visibility of solar eclipses, which depend on the Moon's position relative to the node: a solar eclipse North of the node can be observed if its ecliptic longitude is within 17.7° of the node; South of the node within 8.4° of the node [19]. Ptolemy's observations were made in Alexandria in Egypt (31° N), where he was based. For observers further north, a tighter Southern limit would be expected. This asymmetry is crucial for understanding eclipse prediction on the Antikythera Mechanism [4].
