# Supplementary material for: Eclipse Prediction on the Ancient Greek Astronomical Calculating Machine Known as the Antikythera Mechanism
Source: PLoS One. 2014 Jul 30;9(7):e103275. doi: 10.1371/journal.pone.0103275 (PMC4116162; doi:10.1371/journal.pone.0103275)
Supplement: Figure S3 — The Saros Dial, superimposed on X-ray data of Fragments A, E and F. (PDF) [file pone.0103275.s003.pdf]

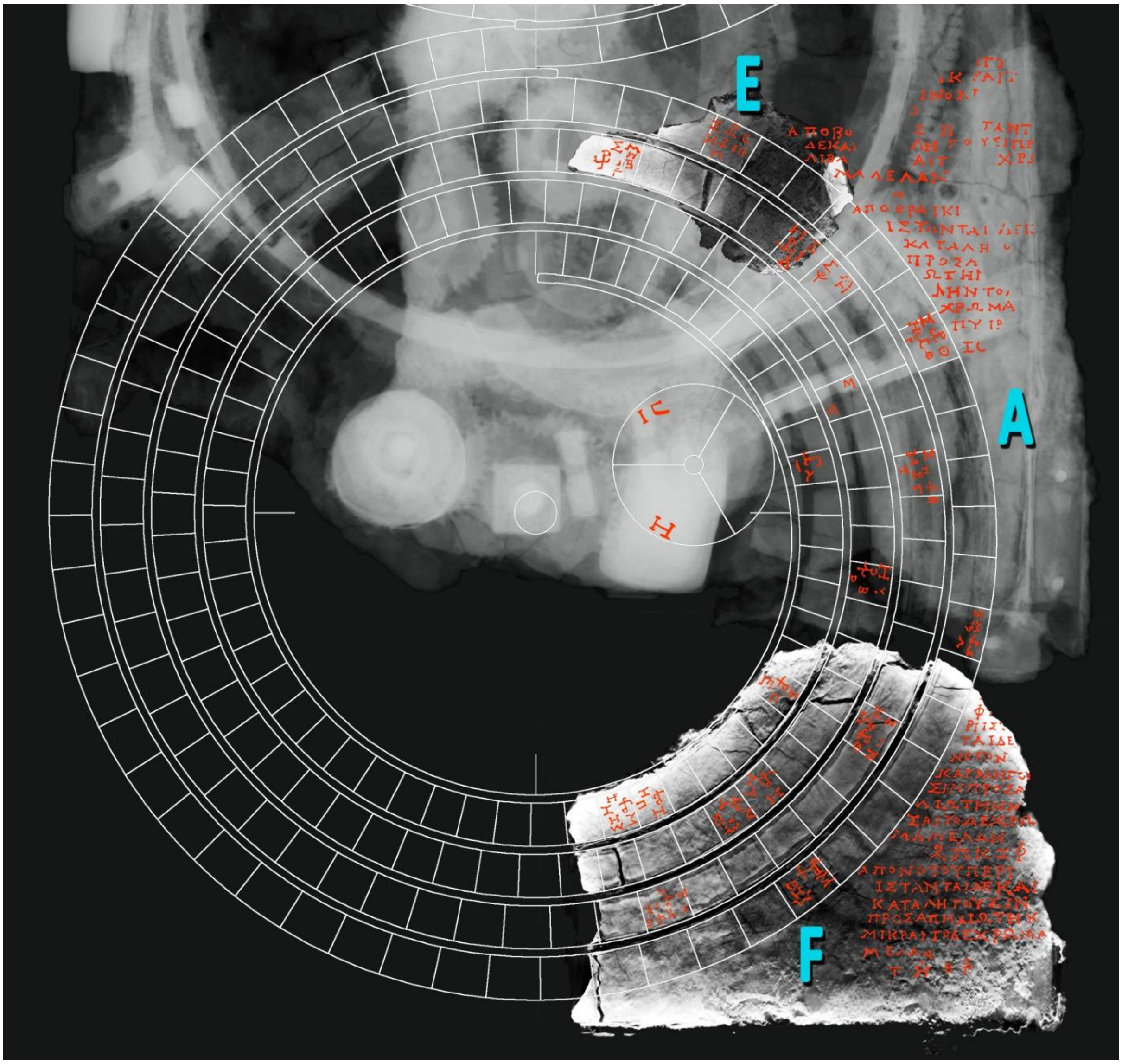

Background data: *Courtesy Antikythera Mechanism Research Project, 2005.* Foreground graphic: *Courtesy Tony Freeth, 2013*

Figure S3 | The Saros Dial, superimposed on X-ray data of Fragments A, E and F. An X-ray radiograph of Fragment A; X-ray CT slices of Fragments E and F. The glyphs and eclipse inscriptions are traced from the data in red. The subsidiary dial is the Exeligmos Dial [1]**.** The diagram of the scales does not follow the visible scales in Fragment A because of distortion in the fragment. The eclipse inscriptions data comes from Fragments A, E and F. Fragment A has some of the inscription on the surface, so both still photographs and PTMs were used to read the inscriptions, augmented by X-ray CT, which enables characters to be read from residual traces beneath the surface. For Fragments E and F, all the data were from X-ray CT, since no text from the back plate is visible on the surface. For discussion of the techniques used to read the inscriptions, see Materials and Methods in the main text.
