# Supplementary material for: Eclipse Prediction on the Ancient Greek Astronomical Calculating Machine Known as the Antikythera Mechanism
Source: PLoS One. 2014 Jul 30;9(7):e103275. doi: 10.1371/journal.pone.0103275 (PMC4116162; doi:10.1371/journal.pone.0103275)
Supplement: Figure S4 — The published inscriptions round the Saros Dial. (PDF) [file pone.0103275.s004.pdf]

A

PRICE: GEARS FROM THE GREEKS

Page 48, Fig. 37 Lower back dial inscription

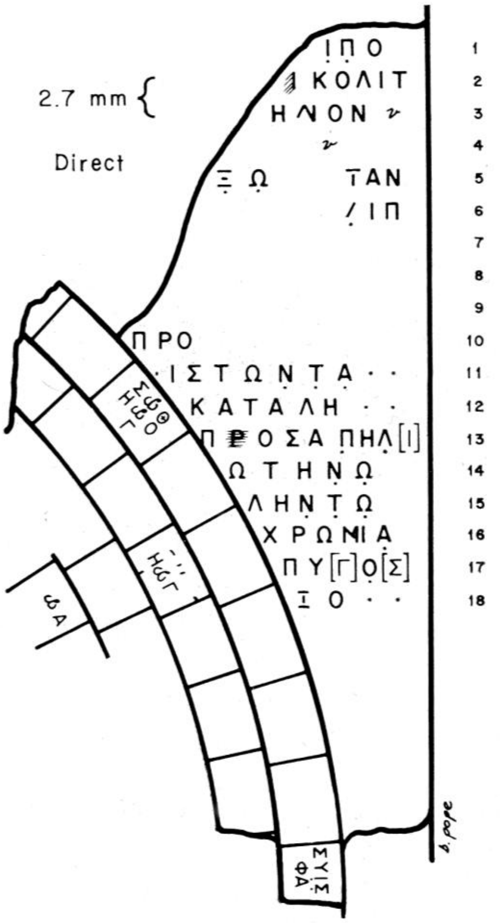

Courtesy American Philosophical Association, 1974

B

Greek Text of Back Plate inscription, near the Lower Back Dial

Black and blue letters are believed to be good, red and orange are dubious. Black and red are from fragments A and F, blue and orange from the other side of fragment E.

1 Ι Π Ο

2 Ι Κ Ο Λ Ι Τ

3 Ι Ν Ο Ν

4 Α Π Ο Χ Ο Ο

5 Δ Ε Κ Α Τ Ξ Ω Ν Τ Α Ν

6 Λ Ι Β Α Ν Χ Ι Π

7 Λ Μ Α

8 Ν

9

10 Π Ρ Ο

11 Ι Σ Τ Ω Ν Τ Α Σ

12 Κ Α Τ Α Λ Η

13 Π Ρ Ο Σ Α Π Η Λ

14 Ω Τ Η Ν Ω

15 Λ Η Ν Τ Ω

16 Χ Ρ Ω Ν Ι Α

17 Π Υ Ο

18 Ι Ο

/ /

19

20 Φ

21 Ρ Ι Ι Σ

22 Τ Α Α Δ

23 Ν Ο Τ Ο Ν

24 Κ Α Ι Α Ρ Η Σ

25 Σ Η Φ Α Ρ Ο Σ

26 Λ Ε Ν Τ Η Ν Κ

27 Σ Α Φ Υ Λ Α Ξ Α Σ

28 Ι Λ Α Μ Ε Λ Α Ν

29 Χ 2 Π Κ Ζ Φ

30 Α Π Ο Ν Ο Τ Ο Υ Π Ε Ρ Ι

31 Ι Σ Π Α Ν Ι Α Σ Δ Ε Κ Α

32 Δ Υ Σ Α Ν

33

34

Reprinted by permission from Macmillan Publishers Ltd: Nature ***444**, Freeth et al., 2006*

Figure S4 | The published inscriptions round the Saros Dial. (**A**) As published in 1974 [6]. (**B**) As published in 2006 [1]. The text in blue and orange on the left of lines 4 - 7 is one row too high.
