# Supplementary material for: Eclipse Prediction on the Ancient Greek Astronomical Calculating Machine Known as the Antikythera Mechanism
Source: PLoS One. 2014 Jul 30;9(7):e103275. doi: 10.1371/journal.pone.0103275 (PMC4116162; doi:10.1371/journal.pone.0103275)
Supplement: Figure S5 — Tracing of the back plate inscriptions. (PDF) [file pone.0103275.s005.pdf]

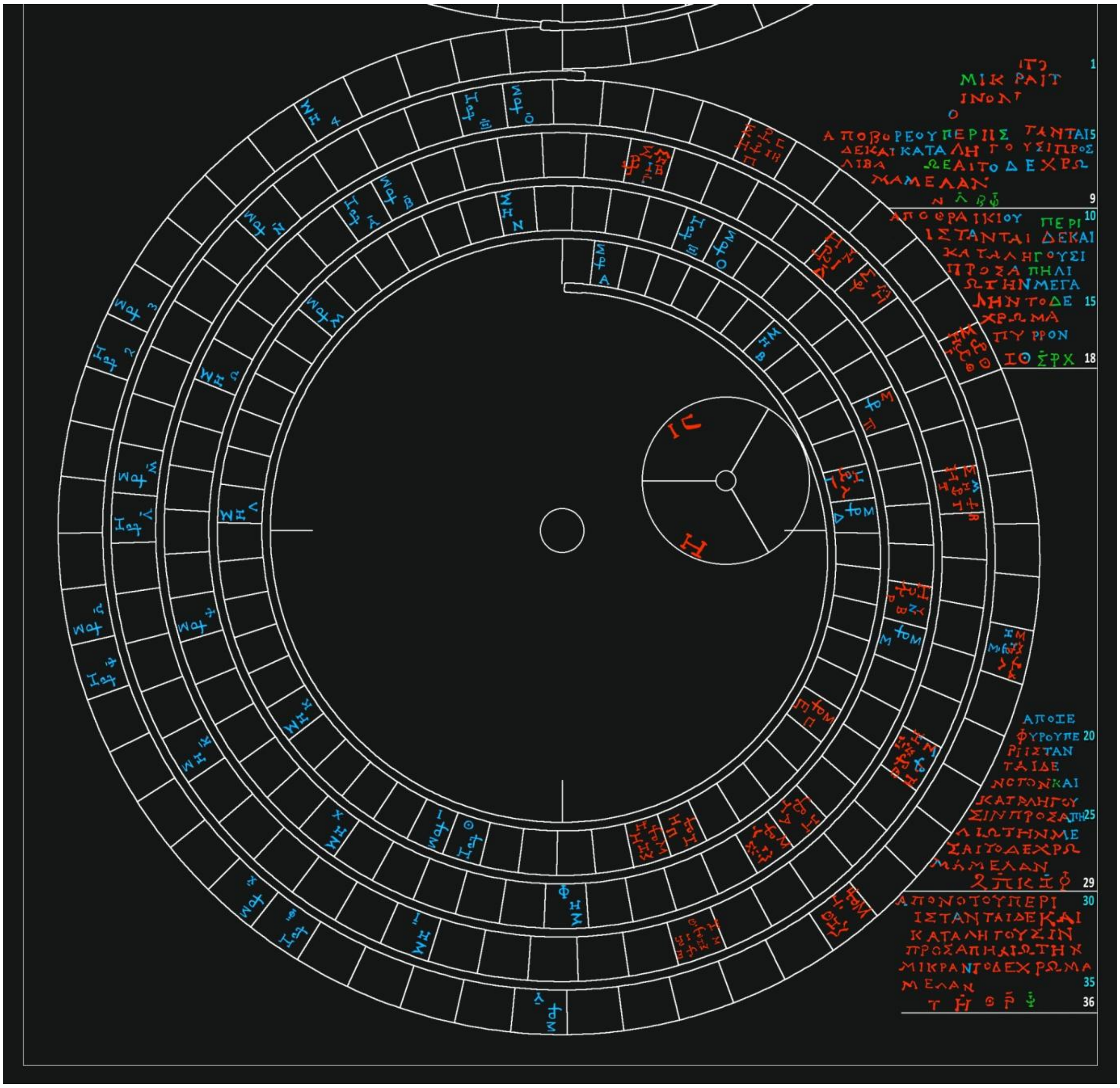

Courtesy Tony Freeth, 2013

Figure S5 | Tracing of the back plate inscriptions. These have been traced from PTMs and more than a hundred X-ray CT slices (see main text Materials and Methods). Text in **red** is traced; text reconstructed from the context in **blue**; uncertain text in **green**. Groups of index letters have been underlined with a white line and have white line numbers. The line numbers have been inherited from previous publications [1], [6]. The inscriptions are divided into sets of 6 - 9 lines, with an *Index Letter Group* under each set of lines. The index letters are the same as those that index the glyphs, with two alphabets—the second with bars on top—and additional extra-alphabetic characters, such as "2" in L. 29. The sets of lines above each Index Letter Group describe characteristics that are shared by all the eclipses, indexed by the letters of this group. There are Index Letter Groups at L. 9, L. 18, L. 29 and L. 36.
