# Supplementary material for: Eclipse Prediction on the Ancient Greek Astronomical Calculating Machine Known as the Antikythera Mechanism
Source: PLoS One. 2014 Jul 30;9(7):e103275. doi: 10.1371/journal.pone.0103275 (PMC4116162; doi:10.1371/journal.pone.0103275)
Supplement: Figure S6 — Data and interpretation for Index Letter Groups. (PDF) [file pone.0103275.s006.pdf]

A

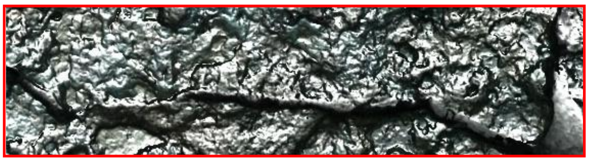

B

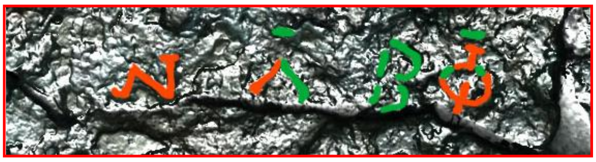

L. 9: N_1, Λ_2, B_1, Φ_2

N_1 is definite. Λ_2 and B_1 would have been difficult to identify without EYM: they are certainly plausible, but only if you are looking for these characters. Φ_2 is convincing but still uncertain.

C

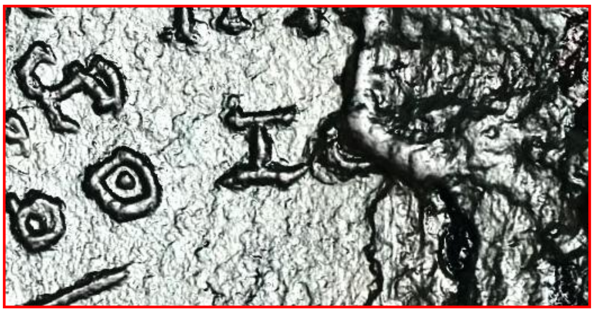

D

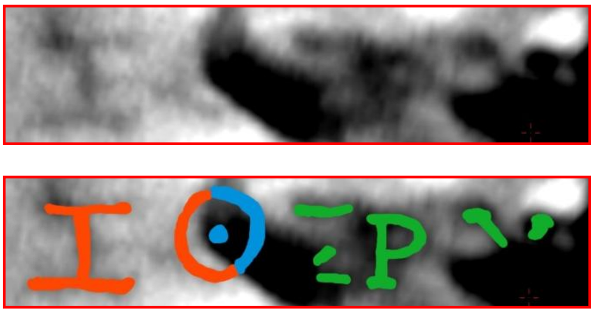

L. 18: Z_1, Θ_1, Σ_2, P_1, X_1

Z_1 is definite and Θ_1 can be reconstructed with confidence. Σ_2, P_1 and X_1 are all uncertain but plausible, though hard to identify without the theory of EYM. P_1 appears to be very likely and Σ_2 and X_1 are consistent with the data. The theory of EYM implies that Σ_2 "ought" to be before Z_1, but it is evident that this is not true.

E

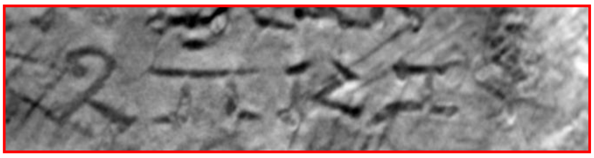

F

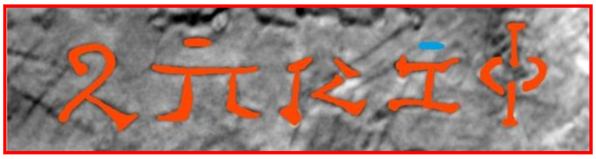

L. 29: 2, Π_2, K_1, Z_2, Φ_1

All of these characters are clear and definite. The first character is the extra-alphabetic symbol "2", which is not known from other ancient Greek inscriptions. The bar on Z is not apparent, but it does not make sense to have Z_1 here, since it is already included in the L. 18 group. It must be inherent in the design that the groups of index letters are mutually exclusive. It appears that the bar was left off by mistake, so it has been restored from context.

G

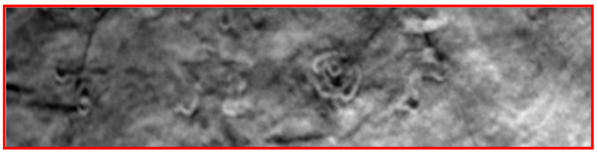

H

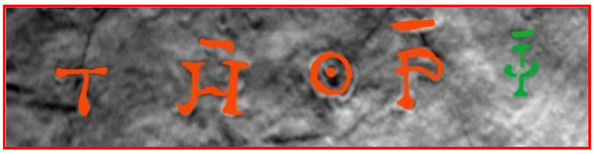

I

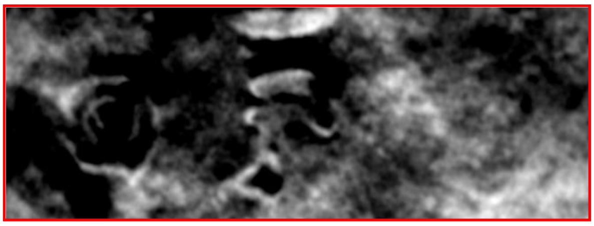

J

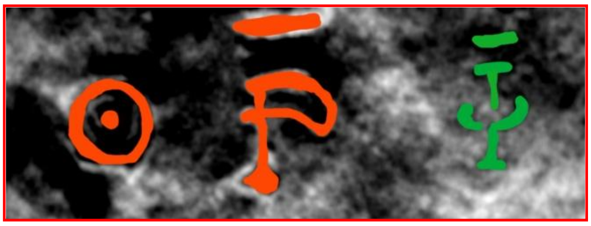

L. 36: T_1, H_2, Θ_1, P_2, Ψ_2

All are clear and definite, except for Ψ_2, which is hard to read, though definitely plausible. In terms of EYM, this character is optional: it is simply a matter of how the EYM limits are set, as discussed in Note S3. Since the characters before Ψ_2 are clear in the X-ray CT, it might be expected that Ψ_2 would also be easy to read. However, the surface of Fragment F is clearly damaged in the region of Ψ_2 but not in the region of the other characters.

Background data: *Courtesy Antikythera Mechanism Research Project, 2005.* Foreground graphics: *Courtesy Tony Freeth, 2013.*

Figure S6 | Data and interpretation for Index Letter Groups. Red = definite; **Blue** = restored from context; **Green** = uncertain. (**A**) - (**B**) **L. 9**: PTM of Fragment A. (**C**) **L. 18**: PTM of Fragment A. (**D**) **L. 18**: X-ray CT of Fragment A. (**E**) - (**F**) **L. 29**: X-ray CT of Fragment A. (**G**) - (**J**) **L. 36**: X-ray CT of Fragment A, with close-ups.
