# Supplementary material for: Eclipse Prediction on the Ancient Greek Astronomical Calculating Machine Known as the Antikythera Mechanism
Source: PLoS One. 2014 Jul 30;9(7):e103275. doi: 10.1371/journal.pone.0103275 (PMC4116162; doi:10.1371/journal.pone.0103275)
Supplement: Figure S7 — Comparative inscription on stone from Hellenistic Corinth. (PDF) [file pone.0103275.s007.pdf]

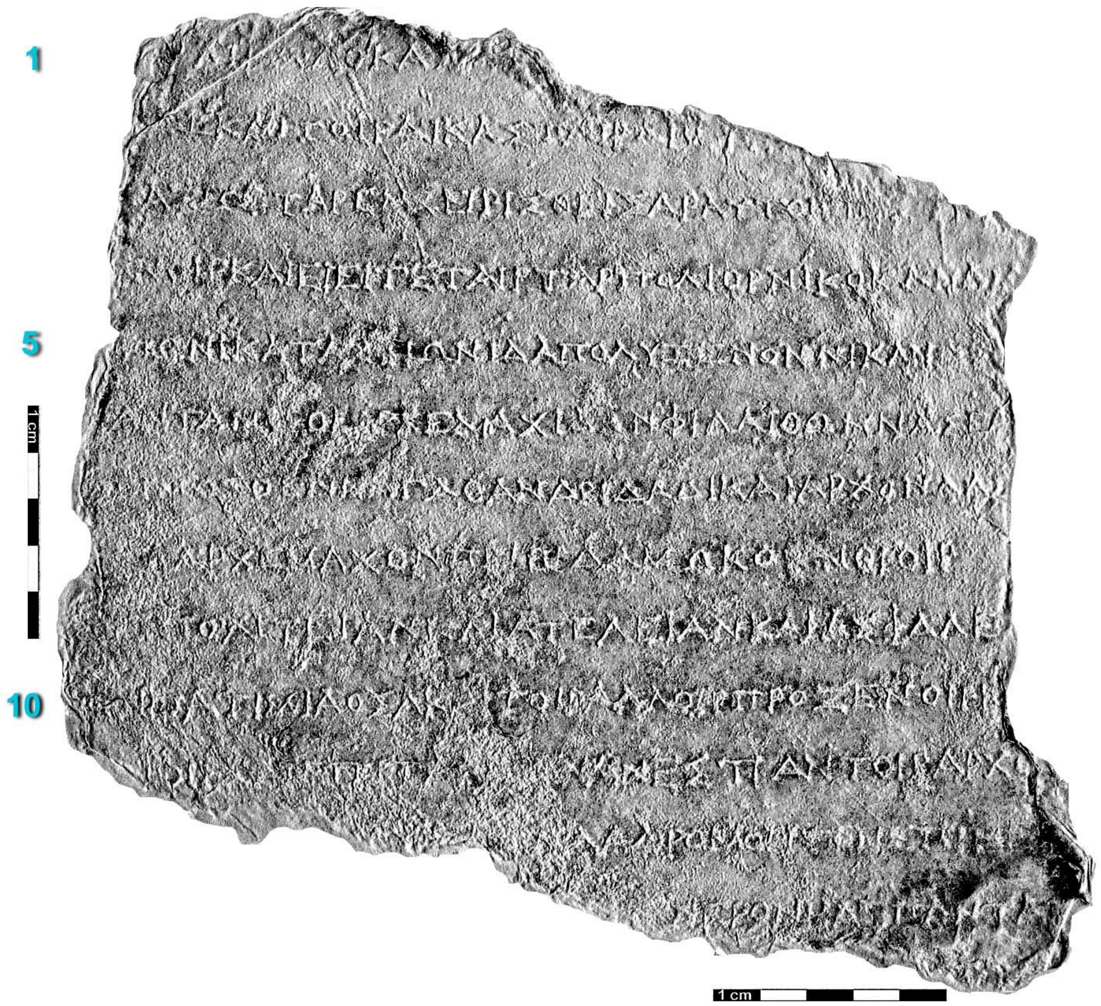

Courtesy Centre for the Study of Ancient Documents, 2013

Figure S7 | Comparative inscription on stone from Hellenistic Corinth. Photograph of a paper squeeze of SEG XXVI 392.
