# Supplementary material for: Eclipse Prediction on the Ancient Greek Astronomical Calculating Machine Known as the Antikythera Mechanism
Source: PLoS One. 2014 Jul 30;9(7):e103275. doi: 10.1371/journal.pone.0103275 (PMC4116162; doi:10.1371/journal.pone.0103275)
Supplement: Figure S8 — Possible definitions of directions of obscuration. (PDF) [file pone.0103275.s008.pdf]

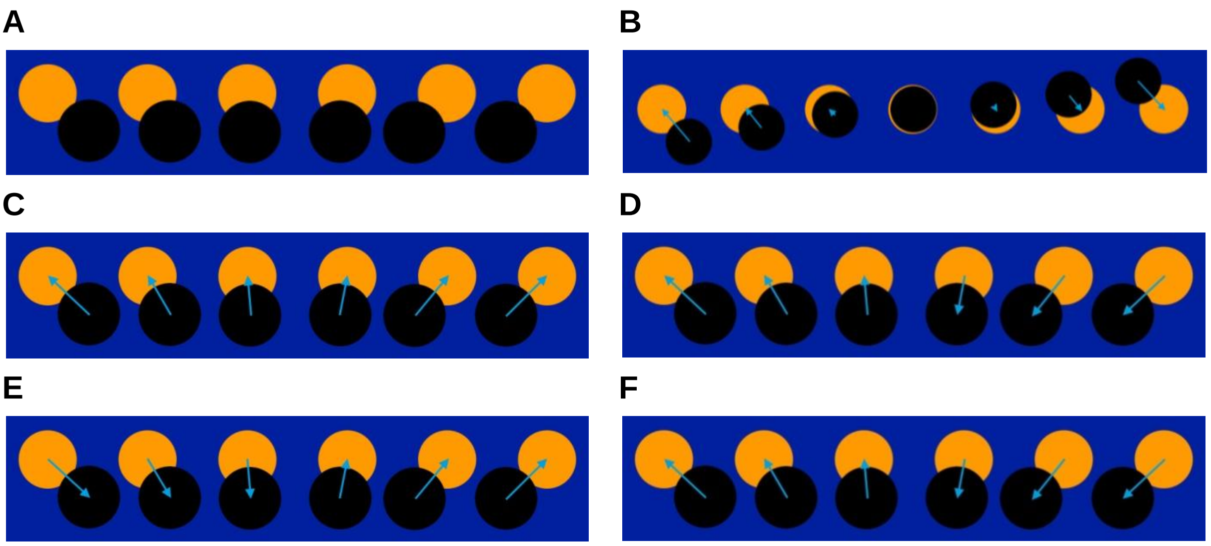

Courtesy Tony Freeth, 2013

Figure S8 | Possible definitions of directions of obscuration. Time progresses from left to right. (**A**) Graphic of total solar eclipse of 2006 Mar-29, seen as a partial eclipse from Puertollano, Spain (39°N, 40°W), which is well North of the eclipse path at this longitude. Gamma = 0.3843. (**B**) Graphic of annular solar eclipse of 2012 May-20, as seen from Chico, California (40°N, 122°W), which was on the eclipse path. Gamma = 0.4828. (**C**) - (**F**) Possible conventions for defining the *shadow vector*. These can be characterized as (**c**) *Always Towards*; (**D**) *Towards then Away*; (**E**) *Away then Towards*. (**F**) *Always Away*.
