# Supplementary material for: Eclipse Prediction on the Ancient Greek Astronomical Calculating Machine Known as the Antikythera Mechanism
Source: PLoS One. 2014 Jul 30;9(7):e103275. doi: 10.1371/journal.pone.0103275 (PMC4116162; doi:10.1371/journal.pone.0103275)
Supplement: Figure S9 — The Saros Dial divided into eclipse years. (PDF) [file pone.0103275.s009.pdf]

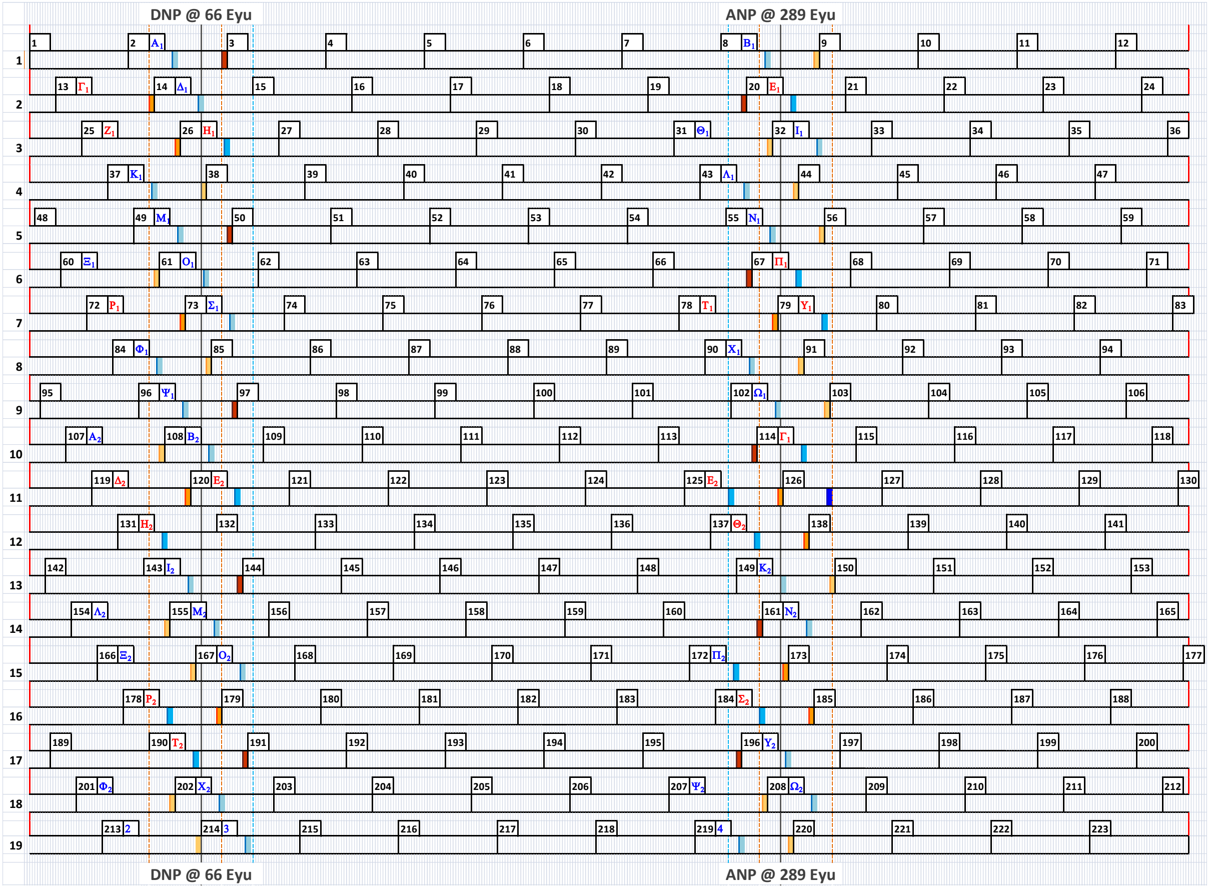

Courtesy Tony Freeth, 2013

Figure S9 | The Saros Dial divided into eclipse years. Eclipse years are numbered on the left. There are 19 eclipse years in total, with 446 EYu in each eclipse year. Eclipses are clustered in the eclipse season around the *Descending Node Point* (DNP). Each month is numbered by its place round the Saros Dial and is divided into 38 EYu. The known glyphs are indicated in bright colours—blue for lunar and orange for solar—with index letters observed in the glyphs in red and reconstructed index letters in dark blue. The paler glyphs are those that are reconstructed by EYM. Solar glyphs, excluded by the solar eclipse asymmetry, are in brown. For typographic reasons, the two alphabets of index letters are distinguished by subscripts, rather than bars on the second alphabet as in the original glyphs. The continuous grey vertical lines show the descending node point at 66 EYu and the ascending node point at 289 EYu from the Saros start. The blue dotted line shows one side of the symmetrical limits for lunar glyphs; the orange dotted lines show the asymmetrical limits for solar glyphs as well as the lunar limit North of the node, which is in the same position as the solar limit.
