# Supplementary material for: Eclipse Prediction on the Ancient Greek Astronomical Calculating Machine Known as the Antikythera Mechanism
Source: PLoS One. 2014 Jul 30;9(7):e103275. doi: 10.1371/journal.pone.0103275 (PMC4116162; doi:10.1371/journal.pone.0103275)
Supplement: Figure S10 — The glyphs that are automatically generated by EYM. (PDF) [file pone.0103275.s010.pdf]

Mnth	LUN	SOL	Ind.	FM EYu	LUN node	N/S	Dist EYu	NM EYu	SOL node	N/S	Dist EYu
1				17				36			
2			A_1	55	D	S	11	74			
3				93				112			
4				131				150			
5				169				188			
6				207				226			
7				245				264			
8			B_1	283	A	N	6	302	A	N	13
9				321				340			
10				359				378			
11				397				416			
12				435				8			
13			Γ_1	27				46	D	N	20
14			Δ_1	65	D	S	1	84			
15				103				122			
16				141				160			
17				179				198			
18				217				236			
19				255				274			
20			E_1	293	A	S	4	312			
21				331				350			
22				369				388			
23				407				426			
24				445				18			
25			Z_1	37				56	D	N	10
26			H_1	75	D	N	9	94			
27				113				132			
28				151				170			
29				189				208			
30				227				246			
31			Θ_1	265				284	A	S	5
32			I_1	303	A	S	14	322			
33				341				360			
34				379				398			
35				417				436			
36				9				28			
37			K_1	47	D	S	19	66	D		0
38				85	D	N	19	104			
39				123				142			
40				161				180			
41				199				218			
42				237				256			
43			Λ_1	275	A	N	14	294	A	N	5
44				313				332			
45				351				370			
46				389				408			
47				427				0			
48				19				38			
49			M_1	57	D	S	9	76			
50				95				114			
51				133				152			
52				171				190			
53				209				228			
54				247				266			
55			N_1	285	A	N	4	304	A	N	15
56				323				342			
57				361				380			
58				399				418			
59				437				10			
60			Ξ_1	29				48	D	N	18
61			O_1	67	D	N	1	86			
62				105				124			
63				143				162			
64				181				200			
65				219				238			
66				257				276			
67			Π_1	295	A	S	6	314			
68				333				352			
69				371				390			
70				409				428			
71				1				20			
72			P_1	39				58	D	N	8
73			Σ_1	77	D	N	11	96			
74				115				134			
75				153				172			
76				191				210			
77				229				248			
78			T_1	267				286	A	S	3
79			Υ_1	305	A	S	16	324			
80				343				362			
81				381				400			
82				419				438			
83				11				30			
84			Φ_1	49	D	S	17	68	D	S	2
85				87				106			
86				125				144			
87				163				182			
88				201				220			
89				239				258			
90			X_1	277	A	N	12	296	A	N	7
91				315				334			
92				353				372			
93				391				410			
94				429				2			
95				21				40			
96			Ψ_1	59	D	S	7	78			
97				97				116			
98				135				154			
99				173				192			
100				211				230			
101				249				268			
102			Ω_1	287	A	N	2	306	A	N	17
103				325				344			
104				363				382			
105				401				420			
106				439				12			
107			A_2	31				50	D	N	16
108			B_2	69	D	N	3	88			
109				107				126			
110				145				164			
111				183				202			
112				221				240			
113				259				278			
114			Γ_2	297	A	S	8	316			
115				335				354			
116				373				392			
117				411				430			
118				3				22			
119			Δ_2	41				60	D	N	6
120			E_2	79	D	N	13	98			
121				117				136			
122				155				174			
123				193				212			
124				231				250			
125			Z_2	269	A	N	20	288	A	S	1
126				307	A	S	18	326			
127				345				364			
128				383				402			
129				421				440			
130				13				32			
131			H_2	51	D	S	15	70	D	S	4
132				89				108			
133				127				146			
134				165				184			
135				203				222			
136				241				260			
137			Θ_2	279	A	N	10	298	A	N	9
138				317				336			
139				355				374			
140				393				412			
141				431				4			
142				23				42			
143			I_2	61	D	S	5	80			
144				99				118			
145				137				156			
146				175				194			
147				213				232			
148				251				270			
149			K_2	289	A	S		308	A	N	19
150				327				346			
151				365				384			
152				403				422			
153				441				14			
154			Λ_2	33				52	D	N	14
155			M_2	71	D	N	5	90			
156				109				128			
157				147				166			
158				185				204			
159				223				242			
160				261				280			
161			N_2	299	A	S	10	318			
162				337				356			
163				375				394			
164				413				432			
165				5				24			
166			Ξ_2	43				62	D	N	4
167			O_2	81	D	N	15	100			
168				119				138			
169				157				176			
170				195				214			
171				233				252			
172			Π_2	271	A	N	18	290	A	N	1
173				309	A	S	20	328			
174				347				366			
175				385				404			
176				423				442			
177				15				34			
178			P_2	53	D	S	13	72	D	S	6
179				91				110			
180				129				148			
181				167				186			
182				205				224			
183				243				262			
184			Σ_2	281	A	N	8	300	A	N	11
185				319				338			
186				357				376			
187				395				414			
188				433				6			
189				25				44			
190			T_2	63	D	S	3	82			
191				101				120			
192				139				158			
193				177				196			
194				215				234			
195				253				272			
196			Υ_2	291	A	S	2	310			
197				329				348			
198				367				386			
199				405				424			
200				443				16			
201			Φ_2	35				54	D	N	12
202			X_2	73	D	N	7	92			
203				111				130			
204				149				168			
205				187				206			
206				225				244			
207			Ψ_2	263				282	A	S	7
208			Ω_2	301	A	S	12	320			
209				339				358			
210				377				396			
211				415				434			
212				7				26			
213			2	45				64	D	N	2
214			3	83	D	N	17	102			
215				121				140			
216				159				178			
217				197				216			
218				235				254			
219			4	273	A	N	16	292	A	N	3
220				311				330			
221				349				368			
222				387				406			
223				425				444			

Courtesy Tony Freeth, 2013

Figure S10 | The glyphs that are automatically generated by EYM. Observed glyphs are shown in bright colours; reconstructed glyphs in paler colours; solar glyphs excluded by the asymmetrical criterion are in dark brown; lunar glyphs excluded by the *consecutive month rule* in dark blue. Observed index letters are in red; reconstructed in blue. The green and purple vertical bars represent *Heptons* and *Octons* (Note S1) for both lunar and solar EPs. The following are shown for each EP: its *index letter* (observed or reconstructed); its *place in EYu in the Eclipse Year*; whether it is at the *Descending or Ascending node point*; whether it is *North or South of the node*; and its *distance in EYu from the node point*.
