# Supplementary material for: Eclipse Prediction on the Ancient Greek Astronomical Calculating Machine Known as the Antikythera Mechanism
Source: PLoS One. 2014 Jul 30;9(7):e103275. doi: 10.1371/journal.pone.0103275 (PMC4116162; doi:10.1371/journal.pone.0103275)
Supplement: Figure S11 — Spreadsheet generating the lunar Index Letter Groups according to EYM. (PDF) [file pone.0103275.s011.pdf]

INDEX LETTER GROUPS - LUNAR

**Parameters:**	Synodic month	**38**	units	29.53	days	**Glyph limits:**	Lunar N	**20**	units
	Month start after N	**2**	units	1.55	days		Lunar S	**20**	units
	Eclipse Year Unit	**1**	unit	0.78	days		Solar N	**21**	units
	Descending node at	**66**	units	102.56	days		Solar S	**7**	units
	Ascending node at	**289**	units	449.11	days				
	Time between node	**223**	units	346.54	days				

Red Index Letter, such as H_1, are those observed in the glyphs
Blue Index Letters, such as B_1, are those reconstructed by EYM

Lunar Glyphs *North of node* - month order

Month No.	8	26	43	55	61	73	90	102	108	120	125	137	155	167	172	184	202	214	219
Index	B_1	H_1	Λ_1	N_1	O_1	Σ_1	X_1	Ω_1	B_2	E_2	Z_2	Θ_2	M_2	O_2	Π_2	Σ_2	X_2	3	4
Eclipse Year, Eyu	283	75	275	285	67	77	277	287	69	79	269	279	71	81	271	281	73	83	273
Node	D	A	D	D	A	A	D	D	A	A	D	D	A	A	D	D	A	A	D
From Node, EYu	6	9	14	4	1	11	12	2	3	13	20	10	5	15	18	8	7	17	16

Lunar Glyphs *North of node* - EYu descending

Month No.	125	172	214	219	167	43	120	90	73	137	26	184	202	8	155	55	108	102	61
Index	Z_2	Π_2	3	4	O_2	Λ_1	E_2	X_1	Σ_1	Θ_2	H_1	Σ_2	X_2	B_1	M_2	N_1	B_2	Ω_1	O_1
Eclipse Year, Eyu	269	271	83	273	81	275	79	277	77	279	75	281	73	283	71	285	69	287	67
Node	D	D	A	D	A	D	A	D	A	D	A	D	A	D	A	D	A	D	A
From Node, EYu	20	18	17	16	15	14	13	12	11	10	9	8	7	6	5	4	3	2	1

Lunar Glyphs *South of node/At node* - month order

Month No.	2	14	20	32	37	49	67	79	84	96	114	131	143	149	161	178	190	196	208
Index	A_1	Δ_1	E_1	I_1	K_1	M_1	Π_1	Y_1	Φ_1	Ψ_1	Γ_2	H_2	I_2	K_2	N_2	P_2	T_2	Y_2	Ω_2
Eclipse Year, Eyu	55	65	293	303	47	57	295	305	49	59	297	51	61	289	299	53	63	291	301
Node	A	A	D	D	A	A	D	D	A	A	D	A	A	D	D	A	A	D	D
From Node, EYu	11	1	4	14	19	9	6	16	17	7	8	15	5	0	10	13	3	2	12

Lunar Glyphs *South of node/At node* - EYU ascending (negative)

Month No.	149	14	196	190	20	143	67	96	114	49	161	2	208	178	32	131	79	84	37
Index	K_2	Δ_1	Y_2	T_2	E_1	I_2	Π_1	Ψ_1	Γ_2	M_1	N_2	A_1	Ω_2	P_2	I_1	H_2	Y_1	Φ_1	K_1
Eclipse Year, Eyu	289	65	291	63	293	61	295	59	297	57	299	55	301	53	303	51	305	49	47
Node	D	A	D	A	D	A	D	A	D	A	D	A	D	A	D	A	D	A	A
From Node, EYu	0	1	2	3	4	5	6	7	8	9	10	11	12	13	14	15	16	17	19

	NORTH: descending order																
	**Conjectural Lunar Group A** *Very far North of node*						**Conjectural Lunar Group B** *Far North of node*						**Conj. Lunar Group C** *Close North of node*				
	125	172	214	219	167	43	120	90	73	137	26	184	202	8	155	55	108
	Z_2	Π_2	3	4	O_2	Λ_1	E_2	X_1	Σ_1	Θ_2	H_1	Σ_2	X_2	B_1	M_2	N_1	B_2
	269	271	83	273	81	275	79	277	77	279	75	281	73	283	71	285	69
	D	D	A	D	A	D	A	D	A	D	A	D	A	D	A	D	A
	20	18	17	16	15	14	13	12	11	10	9	8	7	6	5	4	3
γ	1.13	1.02	1.10	0.87	1.32	0.92	1.03	1.04	0.75	0.63	0.42	0.24	0.42	0.19	0.60	0.26	0.31

			AT	SOUTH: ascending negative order																	
	**Conj. Lunar Group D** *Nearly at node: North then South*					**Conj. Lunar Group E** *Close South of node*					**Conj. Lunar Group F** *Far South of node*					**Conjectural Lunar Group G** *Very far South of node*					
	102	61	149	14	196	190	20	143	67	96	114	49	161	2	208	178	32	131	79	84	37
	Ω_1	O_1	K_2	Δ_1	Y_2	T_2	E_1	I_2	Π_1	Ψ_1	Γ_2	M_1	N_2	A_1	Ω_2	P_2	I_1	H_2	Y_1	Φ_1	K_1
	287	67	289	65	291	63	293	61	295	59	297	57	299	55	301	53	303	51	305	49	47
	D	A	D	A	D	A	D	A	D	A	D	A	D	A	D	A	D	A	D	A	A
	2	1	0	-1	-2	-3	-4	-5	-6	-7	-8	-9	-10	-11	-12	-13	-14	-15	-16	-17	-19
γ	0.26	-0.03	-0.14	-0.29	-0.47	-0.25	-0.50	-0.15	-0.46	-0.47	-0.48	-0.76	-0.91	-0.99	-1.21	-0.98	-1.15	-0.84	-1.19	-1.19	

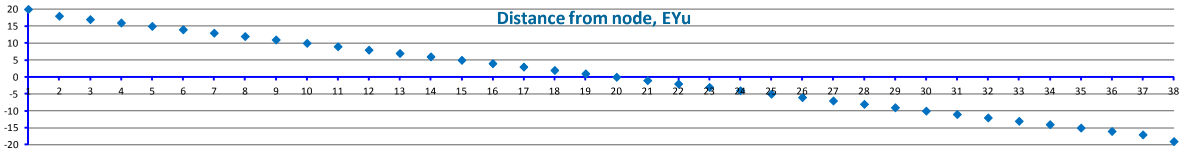

Courtesy Tony Freeth, 2013

Figure S11 | Spreadsheet generating the lunar Index Letter Groups according to EYM. In contrast to the solar groups, the result is nearly symmetrical between North and South and produces a full 38 EP Babylonian scheme, as might be expected, since there is no asymmetry in the visibility of lunar eclipses. There are 19 EPs North of the node and 18 EPs South, with one EP at the node. The lunar EPs break down into four groups of five index letters and three groups of six. Though there is some limited flexibility in deciding on the divisions between the groups, those illustrated appear to be the most natural.
