# Supplementary material for: Eclipse Prediction on the Ancient Greek Astronomical Calculating Machine Known as the Antikythera Mechanism
Source: PLoS One. 2014 Jul 30;9(7):e103275. doi: 10.1371/journal.pone.0103275 (PMC4116162; doi:10.1371/journal.pone.0103275)
Supplement: Figure S12 — Graphic of Total lunar eclipse of 2011 Jun-15. (PDF) [file pone.0103275.s012.pdf]

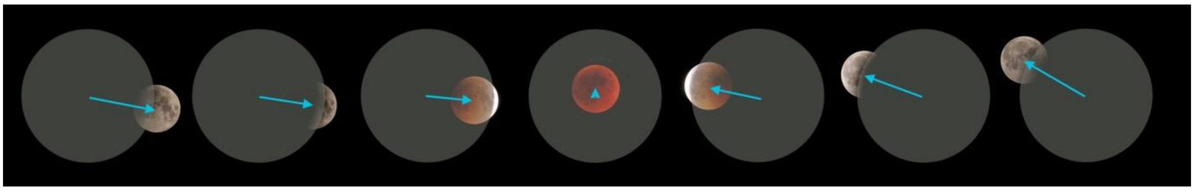

Courtesy Tony Freeth, 2013

Figure S12 | Graphic of Total lunar eclipse of 2011 Jun-15. The sequence should be read from left to right. The eclipse was at the Ascending Node, just North of the node: gamma = 0.0897. The blue arrow shows the directions of the *shadow vector*.
