# Supplementary material for: Eclipse Prediction on the Ancient Greek Astronomical Calculating Machine Known as the Antikythera Mechanism
Source: PLoS One. 2014 Jul 30;9(7):e103275. doi: 10.1371/journal.pone.0103275 (PMC4116162; doi:10.1371/journal.pone.0103275)
Supplement: Figure S13 — Glyph data and its interpretation. (PDF) [file pone.0103275.s013.pdf]

Month	*Fragment*	*Glyph & symbols* / *Symbols*	*Interpretation*
**013**	A-1.1	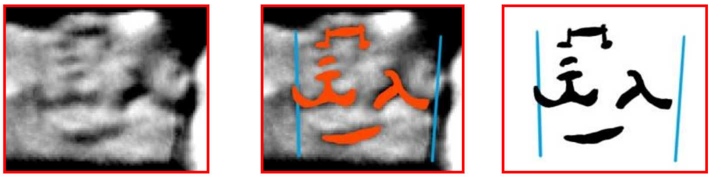	H<br>ω\ρ A or Δ<br>Γ
**020**	F-1.1	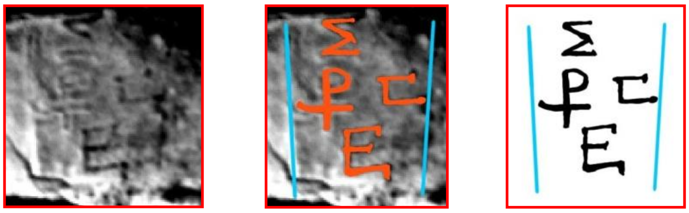	Σ<br>ω\ρ ς<br>E
**025**	F-1.6	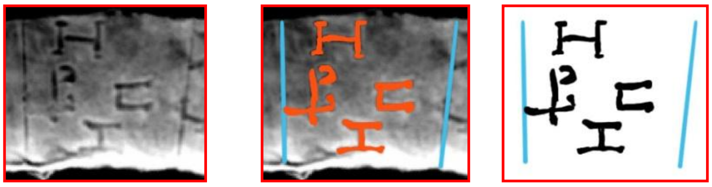	H<br>ω\ρ ς<br>Z
**026**	F-1.7	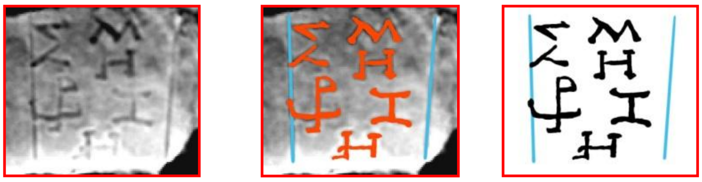	Σ H\M<br>ω\ρ Z<br>H
**067**	A-2.1	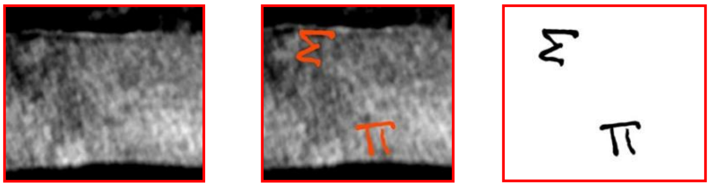	Σ<br>Π
**072**	A-2.2	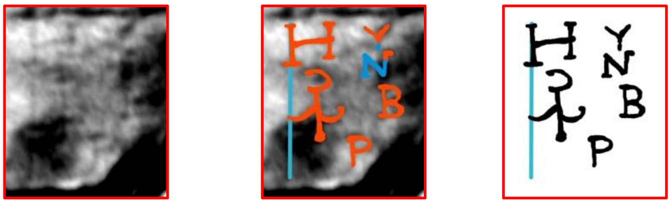	H N\Y<br>ω\ρ B<br>P
**078**	F-2.4	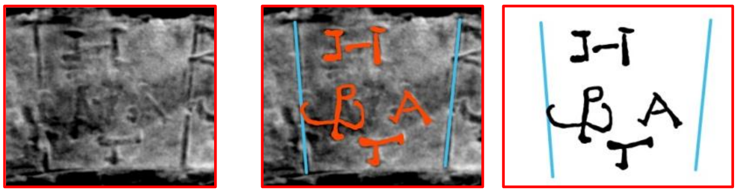	H<br>ω\ρ A<br>T
**079**	F-2.5	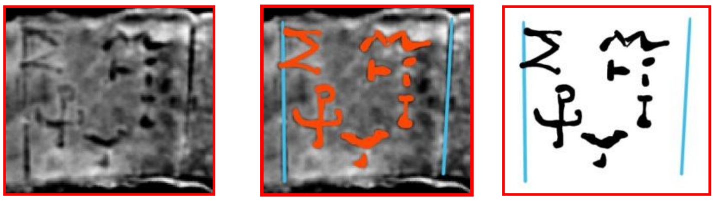	Σ H\M<br>ω\ρ I<br>Y
**114**	E-3.1	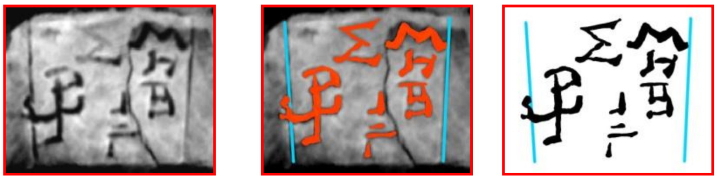	Σ H\M<br>ω\ρ IB<br>$\bar{\Gamma}$

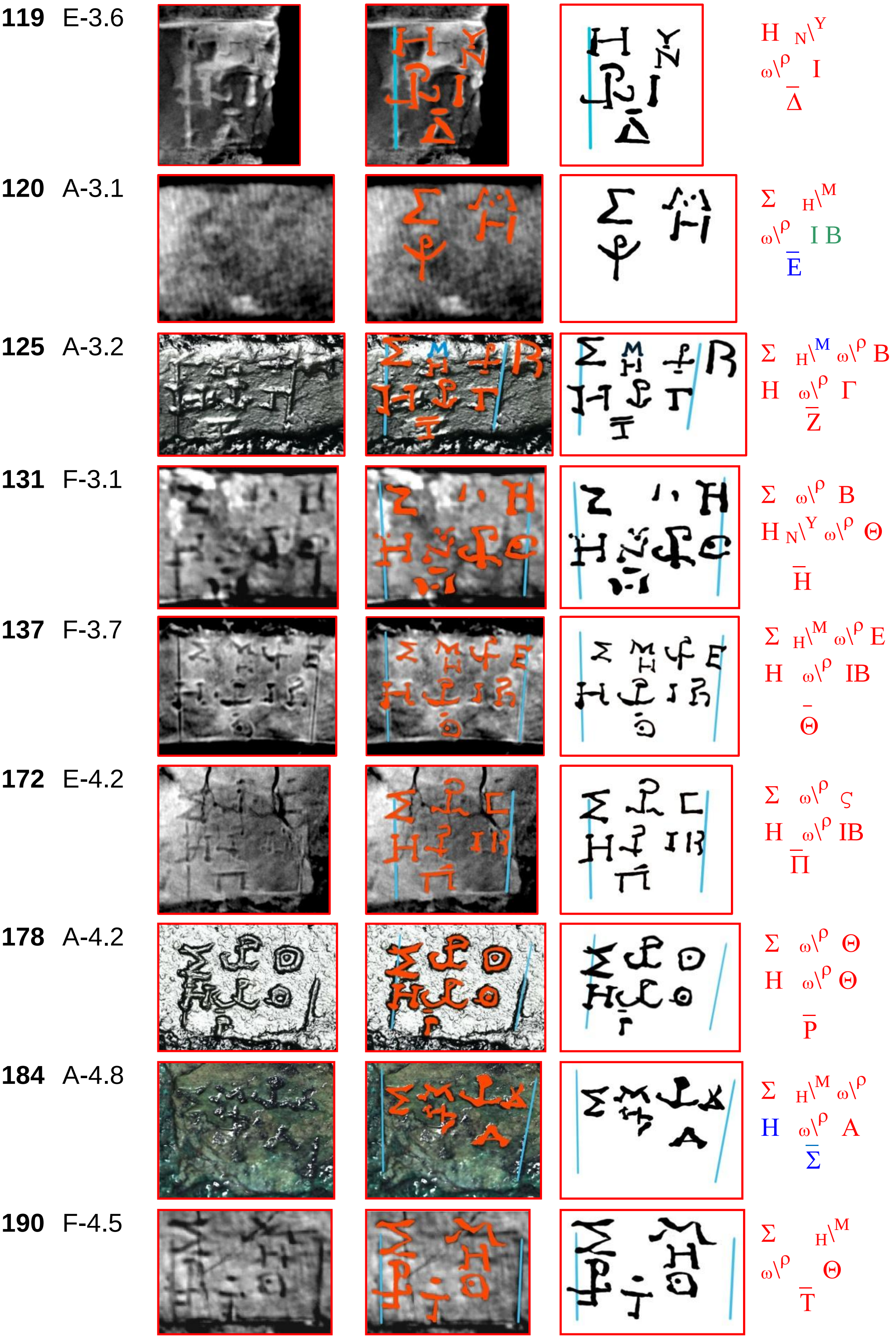

Background data: *Courtesy Antikythera Mechanism Research Project, 2005*. Foreground graphics: *Courtesy Tony Freeth, 2013*.

Figure S13 | Glyph data and its interpretation. ■ Traced from the data. ■ Inferred from the data or context. ■ Uncertain interpretation. Each glyph is numbered by its month number round the four-turn spiral of the 223-lunar month Saros Dial. The label after this denotes the fragment and position of the glyph—for example, F-3.2 means that the glyph was observed in Fragment F on Scale 3 (counting the four turns of the spiral from the inside) and it was the second glyph found round this scale. On the far right is the interpretation of the text.
