# Supplementary material for: Eclipse Prediction on the Ancient Greek Astronomical Calculating Machine Known as the Antikythera Mechanism
Source: PLoS One. 2014 Jul 30;9(7):e103275. doi: 10.1371/journal.pone.0103275 (PMC4116162; doi:10.1371/journal.pone.0103275)
Supplement: Figure S14 — Data for questioned glyph times. (PDF) [file pone.0103275.s014.pdf]

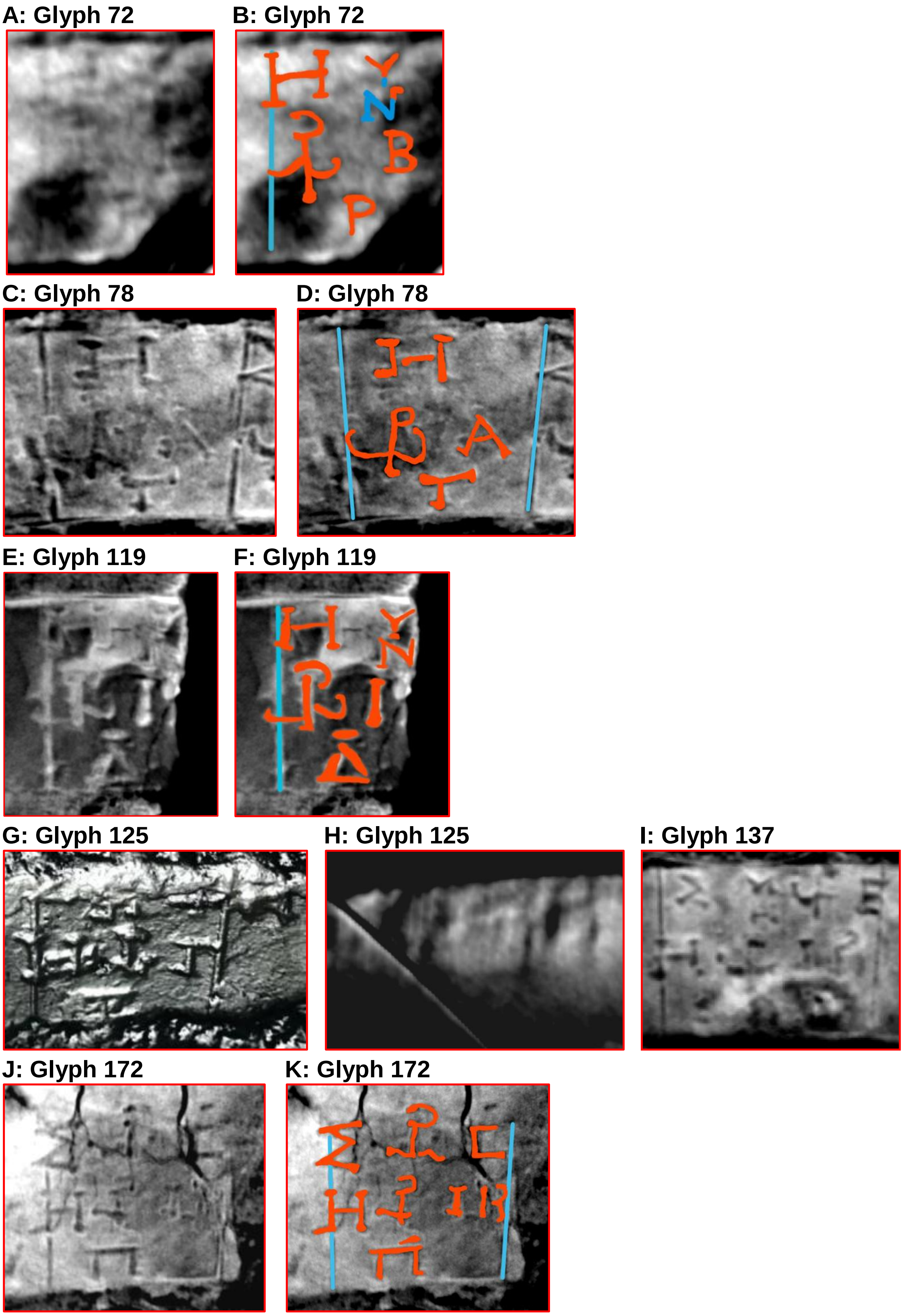

Background data: *Courtesy Antikythera Mechanism Research Project, 2005.* Foreground graphics: *Courtesy Tony Freeth, 2013.*

Figure S14 | Data for questioned glyph times. (**G**) is from PTM; all the rest from X-ray CT. For discussion, see Note S4.
