# Supplementary material for: Eclipse Prediction on the Ancient Greek Astronomical Calculating Machine Known as the Antikythera Mechanism
Source: PLoS One. 2014 Jul 30;9(7):e103275. doi: 10.1371/journal.pone.0103275 (PMC4116162; doi:10.1371/journal.pone.0103275)
Supplement: Figure S15 — Actual month lengths vs ZZM month lengths. (PDF) [file pone.0103275.s015.pdf]

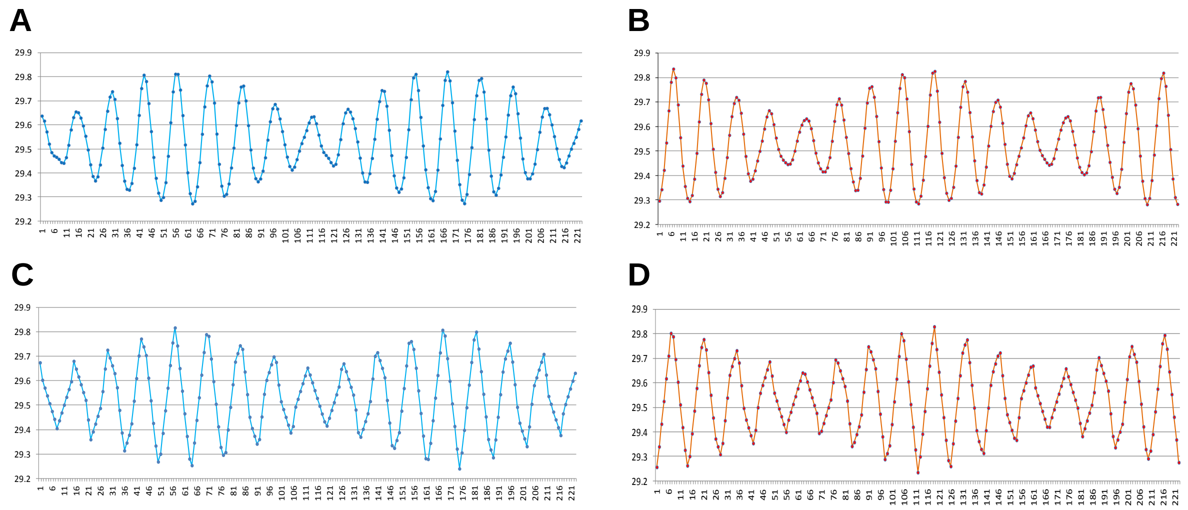

Courtesy Tony Freeth, 2013

Figure S15 | Actual month lengths vs ZZM month lengths. (**A**) Actual month lengths in days, FM to FM. (**B**) Actual month lengths in days, NM to NM. (**C**) ZZM month lengths in days, FM to FM. (**D**) ZZM month lengths in days, NM to NM. For discussion, see Note S4.
