# Supplementary material for: Eclipse Prediction on the Ancient Greek Astronomical Calculating Machine Known as the Antikythera Mechanism
Source: PLoS One. 2014 Jul 30;9(7):e103275. doi: 10.1371/journal.pone.0103275 (PMC4116162; doi:10.1371/journal.pone.0103275)
Supplement: Figure S16 — Graphics showing ZZM. (PDF) [file pone.0103275.s016.pdf]

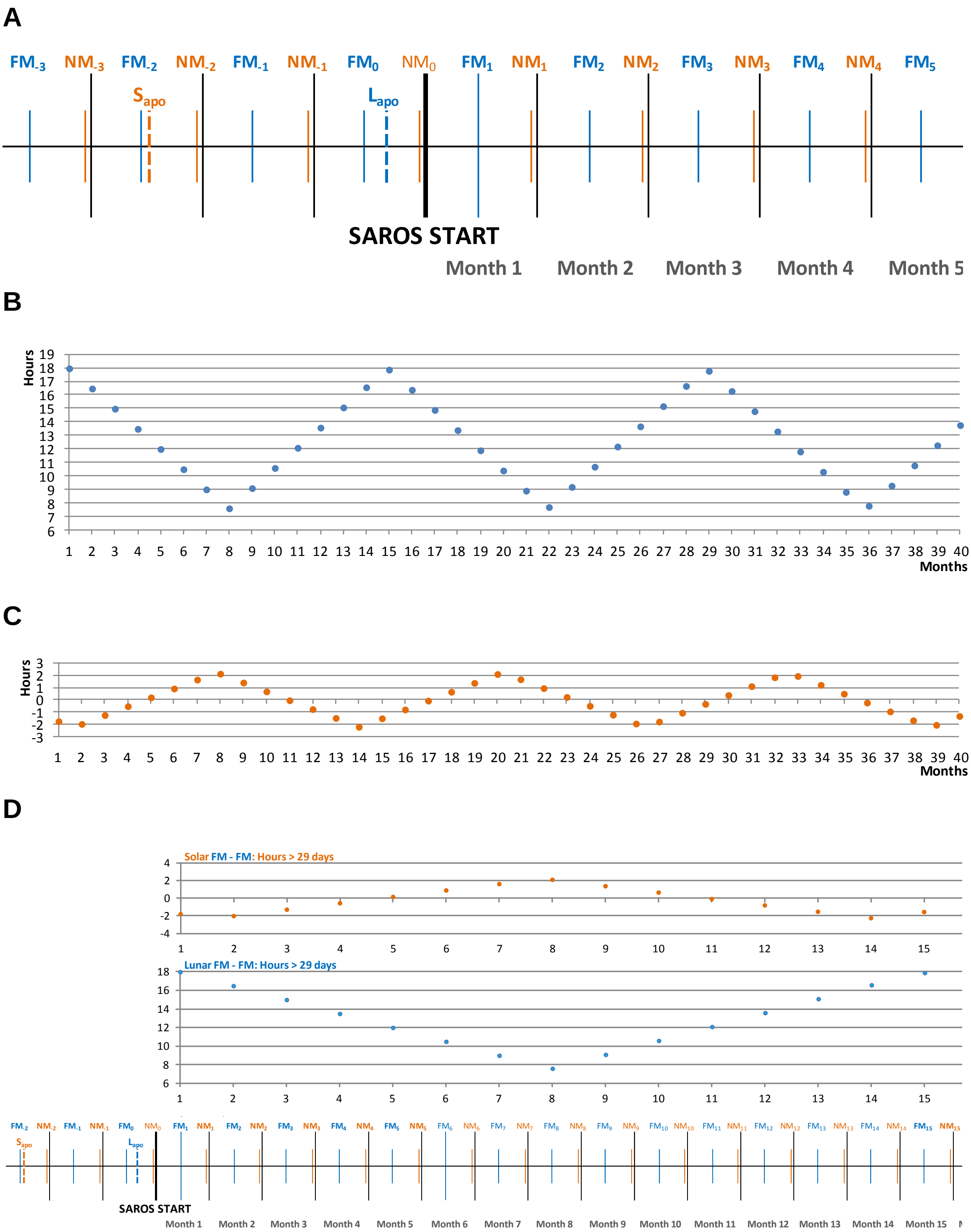

Courtesy Tony Freeth, 2013

Figure S16 | Graphics showing ZZM.

(**A**) The months just before and just after the start of the Saros Dial in ZZM. The orange and blue lines show the positions of New Moon and Full Moon. The black lines show the month starts on the Saros Dial at First Crescent Moon. Arbitrary positions for lunar apogee, L_{apo}, and solar apogee, S_{apo}, are shown.

(**B**) The zigzag function showing lunar contribution to the eclipse times from the *System B* mathematical model. The period of the function is the *Full Moon Cycle* of just under 14 lunar months.

(**C**) The zigzag function showing solar contribution to the eclipse times from the System B mathematical model. The period of the function is the *Year* of just over 12 lunar months.

(**D**) The lunar and solar zigzag contributions in relationship to the months of the Saros Dial. The additions of these contributions create the month lengths illustrated in Figure S15 (**C**).
