# Supplementary material for: Eclipse Prediction on the Ancient Greek Astronomical Calculating Machine Known as the Antikythera Mechanism
Source: PLoS One. 2014 Jul 30;9(7):e103275. doi: 10.1371/journal.pone.0103275 (PMC4116162; doi:10.1371/journal.pone.0103275)
Supplement: Figure S17 — Excel spreadsheet that calculates ZZM with arbitrary input parameters. (PDF) [file pone.0103275.s017.pdf]

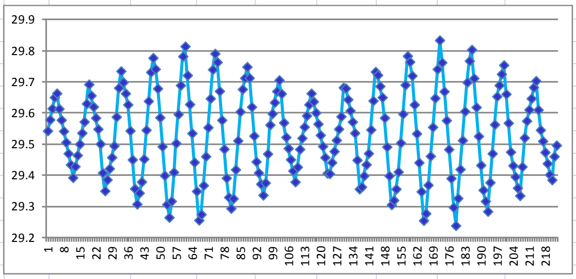

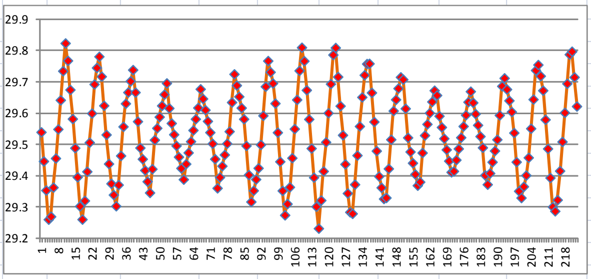

Synodic month	29.531	*days*
Anomalistic month	27.554	*days*
Year	365.250	*days*
Min lu 29d +	7.36	
Max lu 29d +	18.14	
Min sc 29d +	-2.12	
Max sc 29d +	2.12	
Lun anom at FM_1	20.000	days
Sol anom at FM_1	240.000	days

LUNAR			*RMS err*	*Round err*
Month	*Glyph t*	*Calc t*	5.35	5.27
20	18	20.35	2.346	2
26	7	20.57	10.428	10
79	10	8.26	1.735	2
114	12	10.68	1.322	1
125	2	6.39	4.391	4
131	14	18.07	4.067	4
137	5	21.94	7.062	7
172	18	13.40	4.604	5
178	21	17.62	3.384	3
184	4	12.77	8.769	9
190	9	9.34	0.342	0

Total	*Round err*
L + S	6.45

Lun anom at FM_1	20.000	days
Sol anom at FM_1	240.000	days

SOLAR			*RMS err*	*Round err*
Month	*Glyph t*	*Calc t*	8.10	7.55
25	6	22.15	7.846	8
72	14	22.95	8.954	9
78	1	13.58	11.416	11
119	22	3.44	5.436	5
125	3	8.90	5.903	6
131	21	14.78	6.217	6
137	12	12.66	0.656	1
172	12	17.08	5.082	5
178	9	23.00	10.003	10
184	1	10.45	9.454	9

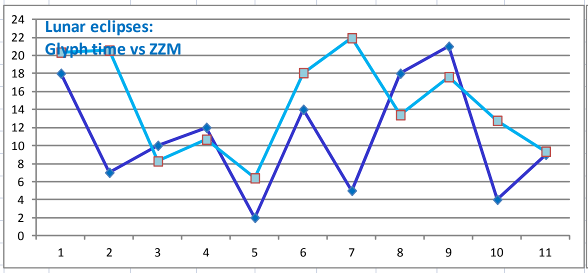

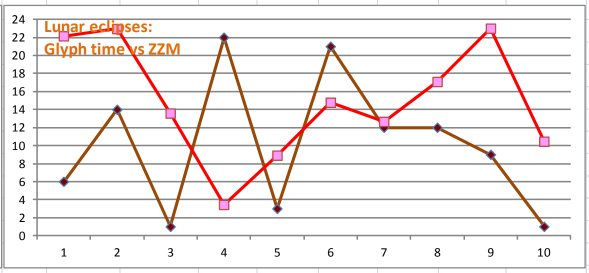

Lun anom at FM_1	20.00	days	Min	29.238
Sol anom at FM_1	240.00	days	Mean	29.535
Lun ecl time	8.00	hrs mech	Max	29.833

	LUNAR	LUNAR	LUNAR	LUNAR	LUNAR	LUNAR	LUNAR	LUNAR
	L_{apo}-FM	Lun>29d	S_{apo}-FM	Sol>29d	Tot>29d	Month	Acc	Time rel
Mnth	*an. mnths*	*hours*	*years*	*hours*	*hours*	*days*	*days*	*hours*
1	*0.726*	*12.229*	*0.657*	*0.788*	*13.017*	*29.542*	0.000	8.000
2	0.798	13.776	0.738	0.102	13.879	29.578	29.578	21.879
3	0.869	15.323	0.819	-0.583	14.740	29.614	59.192	12.618
4	0.941	16.870	0.900	-1.269	15.601	29.650	88.842	4.220
5	0.013	17.863	0.980	-1.955	15.909	29.663	118.505	20.128
6	0.085	16.316	0.061	-1.600	14.716	29.613	148.119	10.844
7	0.156	14.769	0.142	-0.914	13.855	29.577	177.696	0.699
8	0.228	13.222	0.223	-0.229	12.994	29.541	207.237	13.693
9	0.300	11.675	0.304	0.457	12.132	29.506	236.743	1.825
10	0.372	10.129	0.385	1.143	11.271	29.470	266.212	13.097
11	0.443	8.582	0.466	1.828	10.410	29.434	295.646	23.506
12	0.515	7.685	0.546	1.726	9.411	29.392	325.038	8.918

Lun anom at NM_1	7.21	days	Min	29.232
Sol anom at NM_1	254.77	days	Mean	29.528
Sol ecl time	12.00	hrs mech	Max	29.823

	SOLAR	SOLAR	SOLAR	SOLAR	SOLAR	SOLAR	SOLAR	SOLAR
	L_{apo}-NM	Lun>29d	S_{apo}-FM	Sol>29d	Tot>29d	Month	Acc	Time rel
Mnth	*an. mnths*	*hours*	*years*	*hours*	*hours*	*days*	*days*	*hours*
1	*0.262*	*12.497*	*0.698*	*0.445*	*12.942*	*29.539*	0.000	12.000
2	0.333	10.950	0.778	-0.240	10.710	29.446	29.446	22.710
3	0.405	9.403	0.859	-0.926	8.477	29.353	58.799	7.187
4	0.477	7.856	0.940	-1.612	6.245	29.260	88.060	13.432
5	0.549	8.410	0.021	-1.943	6.468	29.269	117.329	19.900
6	0.620	9.957	0.102	-1.257	8.700	29.363	146.692	4.600
7	0.692	11.504	0.183	-0.571	10.933	29.456	176.147	15.533
8	0.764	13.051	0.263	0.114	13.165	29.549	205.696	4.698
9	0.836	14.598	0.344	0.800	15.398	29.642	235.337	20.096
10	0.907	16.145	0.425	1.485	17.630	29.735	265.072	13.726
11	0.979	17.692	0.506	2.069	19.761	29.823	294.895	9.487
12	0.051	17.041	0.587	1.383	18.425	29.768	324.663	3.912

Courtesy Tony Freeth, 2013

Figure S17 | Excel spreadsheet that calculates ZZM with arbitrary input parameters. Only the first 12 rows of 223 rows of calculation are shown. For these parameters, the match is very poor.
