# Supplementary material for: Eclipse Prediction on the Ancient Greek Astronomical Calculating Machine Known as the Antikythera Mechanism
Source: PLoS One. 2014 Jul 30;9(7):e103275. doi: 10.1371/journal.pone.0103275 (PMC4116162; doi:10.1371/journal.pone.0103275)
Supplement: Figure S18 — Lunar eclipses for matching sequence beginning-04 May-12. (PDF) [file pone.0103275.s018.pdf]

Conjectural Lunar Group A—*Very far North of node*

Month 125 - D | **Month 172 - D** | **Month 214 - A** | **Month 219 - D** | **Month 167 - A** | **Month 43 - D**

No eclipse

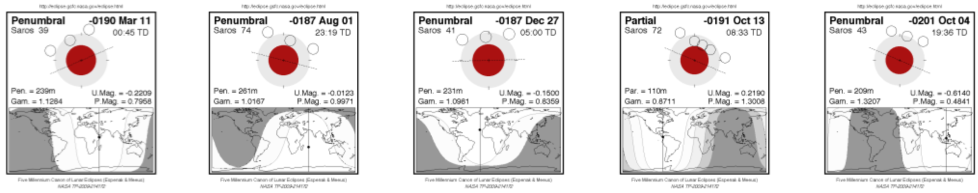

Conjectural Lunar Group B—*Far North of node*

Month 120 - A | **Month 90 - D** | **Month 73 - A** | **Month 137 - D** | **Month 26 - A** | **Month 184 - D**

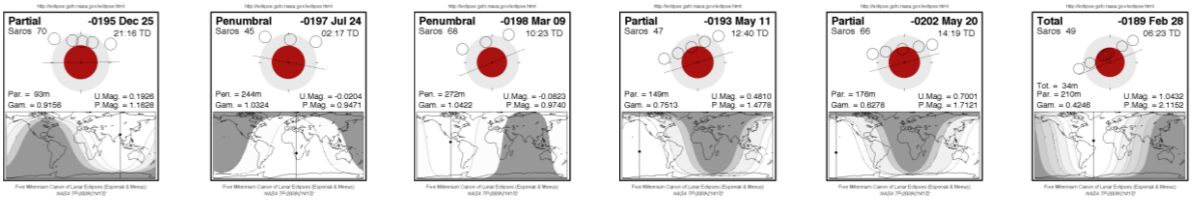

Conjectural Lunar Group C—*Close North of node*

Month 202 - A | **Month 8 - D** | **Month 155 - A** | **Month 55 - D** | **Month 108 - A**

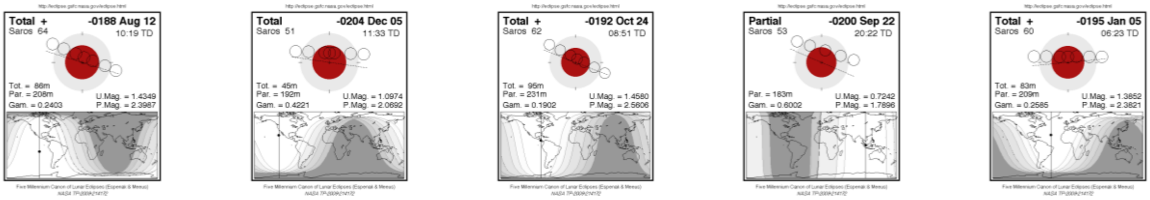

Conjectural Lunar Group D—*Nearly at node: North then South*

Month 102 - D | **Month 61 - A** | **Month 149 - D** | **Month 14 - A** | **Month 196 - D**

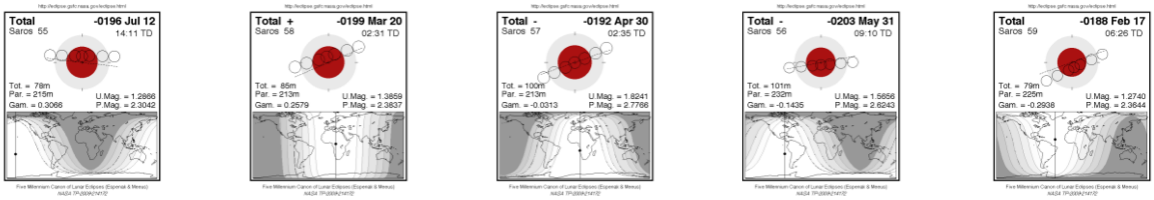

Conjectural Lunar Group E—*Close South of node*

Month 190 - D | **Month 20 - A** | **Month 143 - 0** | **Month 67 - A** | **Month 96 - D**

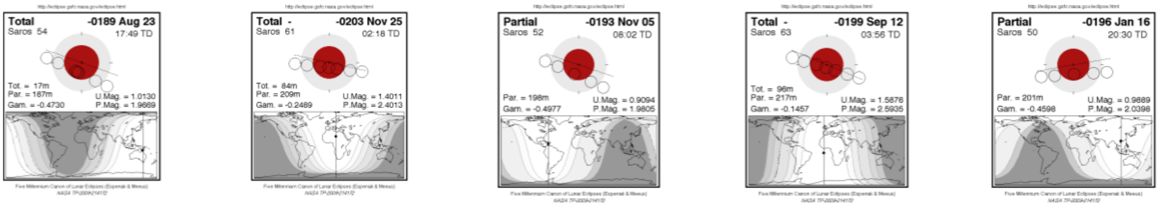

Conjectural Lunar Group F—*Far South of node*

Month 114 - D | **Month 49 - A** | **Month 161 - D** | **Month 2 - A** | **Month 208 - D**

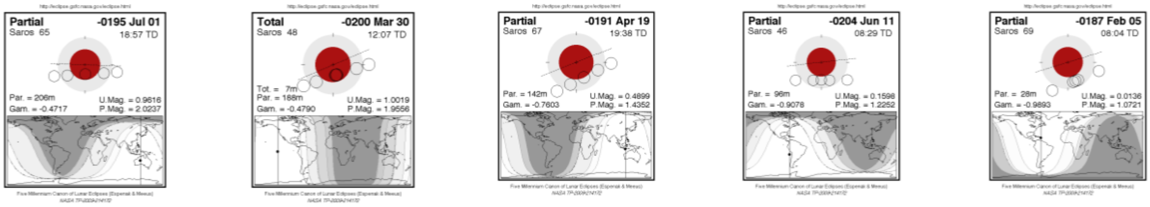

Conjectural Lunar Group G—*Very far South of node*

Month 178 - A | **Month 32 - D** | **Month 131 - A** | **Month 79 - D** | **Month 84 - A** | **Month 37 - A**

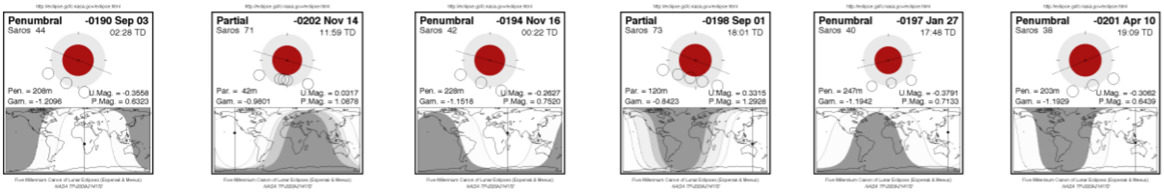

Eclipse map/figure/table/predictions courtesy of Fred Espenak, NASA/Goddard Space Flight Center

Figure S18 | Lunar eclipses for sequence beginning -204 May-12 [14], ordered by Index Letter groups.
