# Supplementary material for: Eclipse Prediction on the Ancient Greek Astronomical Calculating Machine Known as the Antikythera Mechanism
Source: PLoS One. 2014 Jul 30;9(7):e103275. doi: 10.1371/journal.pone.0103275 (PMC4116162; doi:10.1371/journal.pone.0103275)
Supplement: Figure S19 — Solar eclipse paths for matching sequence beginning-204 May-12. (PDF) [file pone.0103275.s019.pdf]

Conjectural Solar Group A—*Very far North of node*

Month 13 - D | Month 149 - A | Month 60 - D | Month 102 - A | Month 107- D

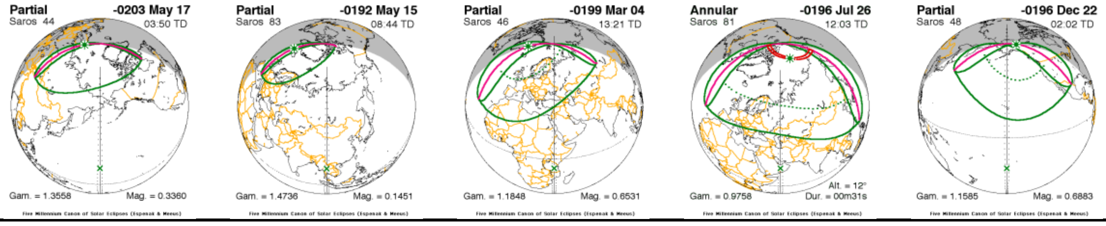

L. 9 Group—*Far North of node*

Month 55 - A | Month 154 - D | Month 8 - A | Month 201 - D

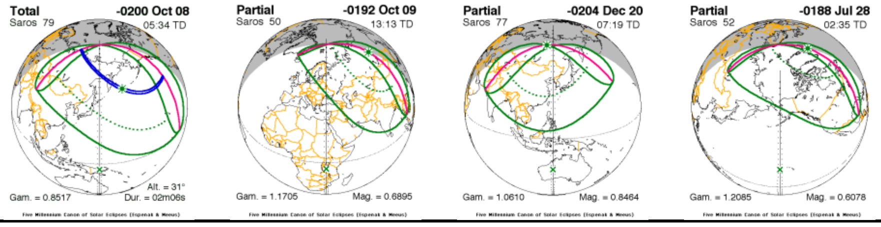

L. 18 Group—*Quite close North of node*

Month 184 - A | Month 25 - D | Month 137 - A | Month 72 - D | Month 90 - A

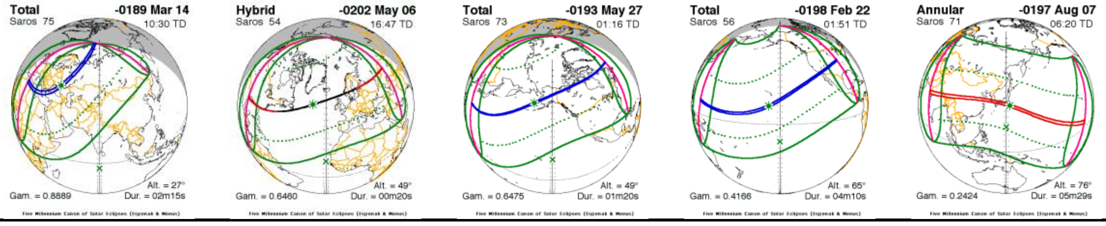

Conjectural Solar Group D—*Close North of node*

Month 119 - D | Month 43 - A | Month 166 - D | Month 219 - A

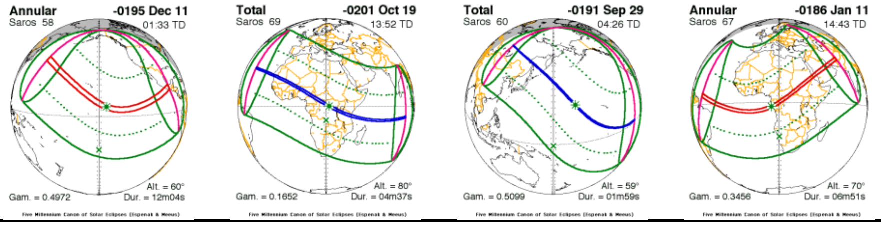

L. 29 Group—*Nearly at node*

Month 213 - D | Month 172 - A | Month 37 - 0 | Month 125 - A | Month 84 - D

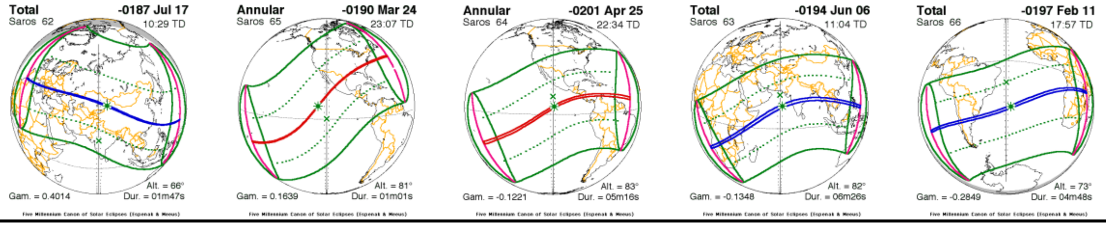

L. 36 Group—*South of node*

Month 78 - A | Month 131 - D | Month 31 - A | Month 178 - D | Month 207 - A

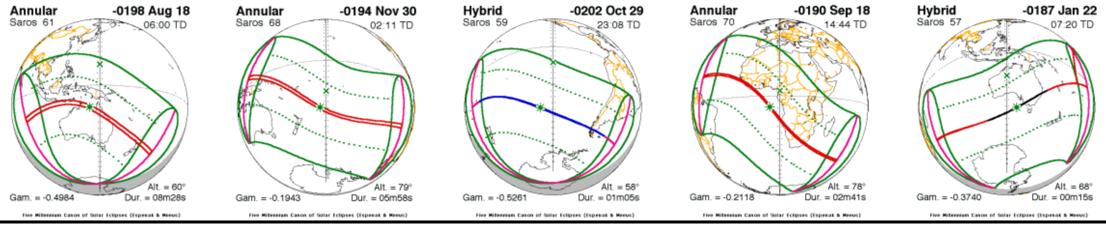

Eclipse map/figure/table/predictions courtesy of Fred Espenak, NASA/Goddard Space Flight Center

Figure S19 | Solar eclipse paths for sequence beginning -204 May-12 [14], ordered by Index Letter groups.
