# Supplementary material for: Eclipse Prediction on the Ancient Greek Astronomical Calculating Machine Known as the Antikythera Mechanism
Source: PLoS One. 2014 Jul 30;9(7):e103275. doi: 10.1371/journal.pone.0103275 (PMC4116162; doi:10.1371/journal.pone.0103275)
Supplement: Figure S20 — Theoretical EYu vs Actual gamma for sequence starting-204 May-12. (PDF) [file pone.0103275.s020.pdf]

A

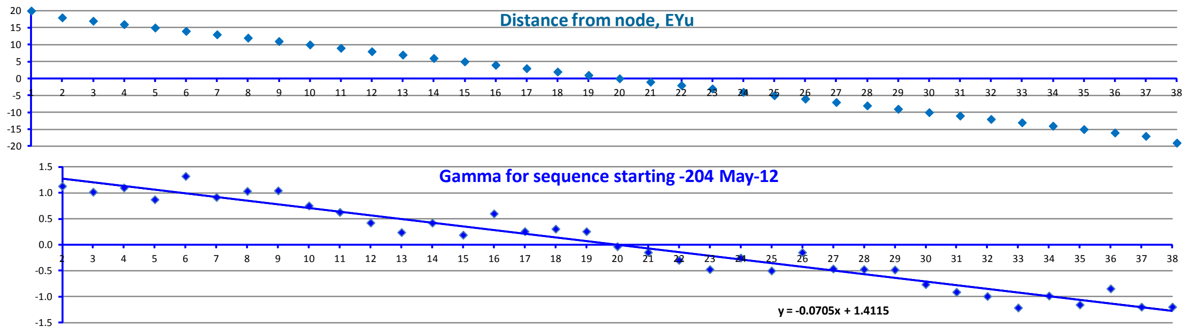

B

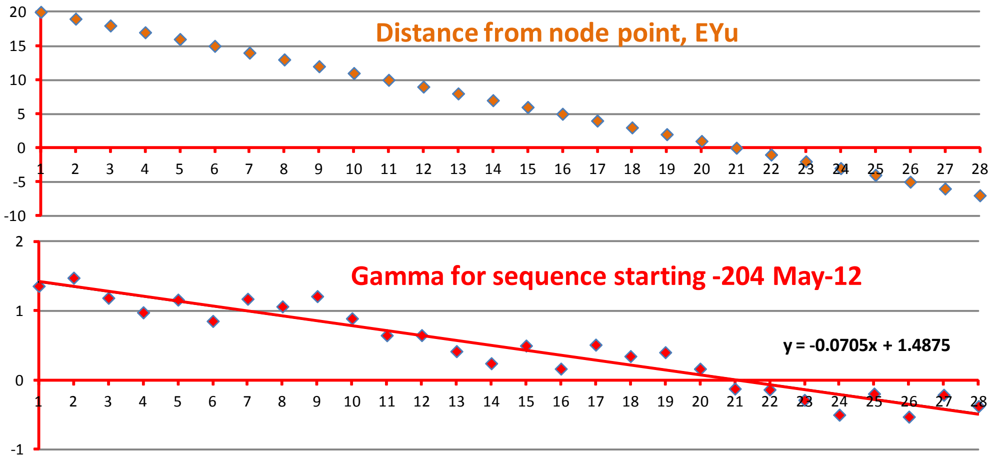

Courtesy Tony Freeth, 2013

Figure S20 | Theoretical EYu vs Actual gamma for sequence starting -204 May-12. (**A**) is for lunar eclipses and (**B**) for solar eclipses. Both theoretical and actual figures are ordered by the Index Letter Group ordering of EYM. For both lunar and solar eclipses, though the relationship between the two graphs is by no means exact, the trend is clear. Inclusion of an EP in a particular Index Letter Group can be viewed as a measure of gamma, which is closely related to the ecliptic latitude of the Moon at the eclipse. In addition, it should be noted that the North-South divide is in complete agreement between the EPs in the model and actual eclipses. The graph of gamma in (**A**) starts at 2 because there was no lunar eclipse in month 125, where the syzygy was furthest North of the node. See the discussion in Note S5.
