# Supplementary material for: Eclipse Prediction on the Ancient Greek Astronomical Calculating Machine Known as the Antikythera Mechanism
Source: PLoS One. 2014 Jul 30;9(7):e103275. doi: 10.1371/journal.pone.0103275 (PMC4116162; doi:10.1371/journal.pone.0103275)
Supplement: Figure S21 — The Saros Dial and the Full Moon Cycle. (PDF) [file pone.0103275.s021.pdf]

A

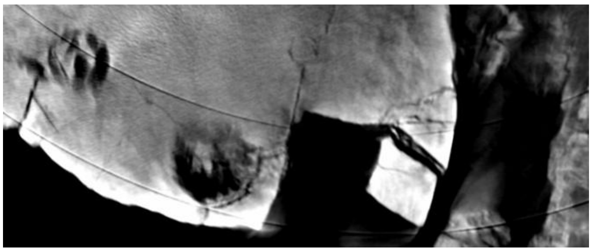

B

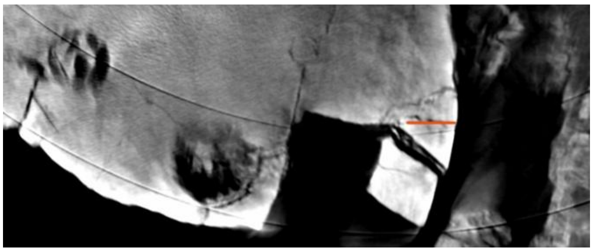

A, B: *Data courtesy Antikythera Mechanism Research Project, 2005.*

C

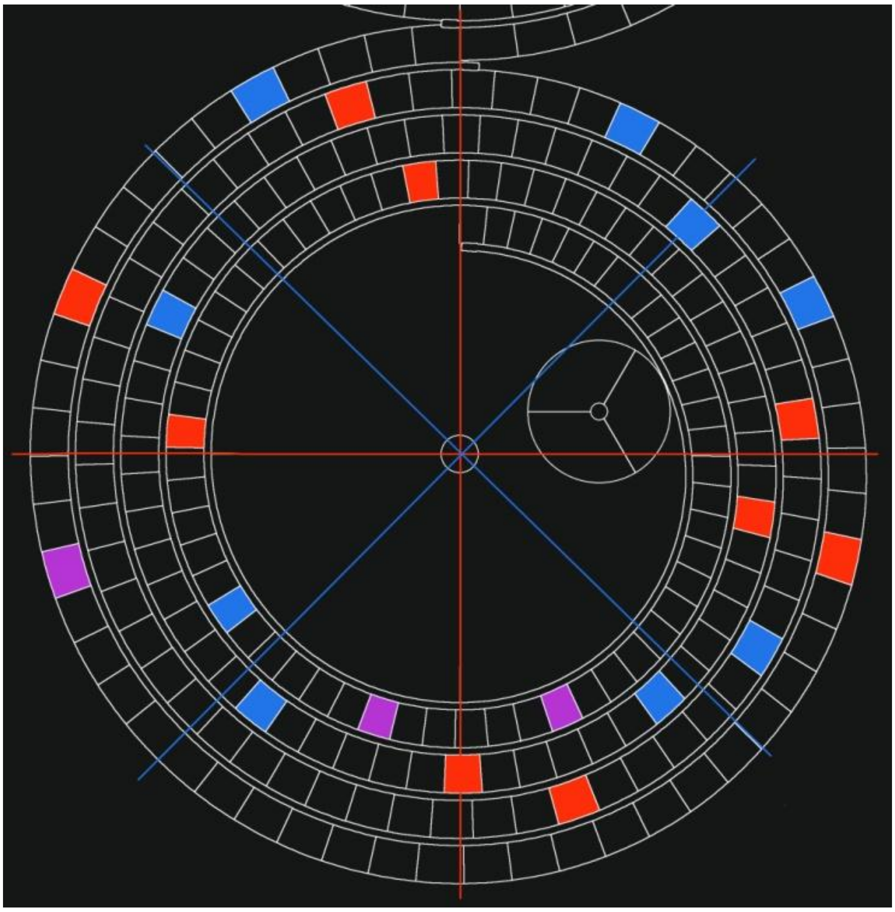

C: *Courtesy Tony Freeth, 2013*

Figure S21 | The Saros Dial and the Full Moon Cycle.

(**A**) X-ray CT of Fragment A, showing possible Full Moon Cycle mark at the 3 o'clock position of the central disk of the Saros Dial. (**B**) The mark is highlighted in red.

(**C**) Central eclipses from the matching sequence starting in -204 May-12. Total ■, Annular ■ and Hybrid ■ solar eclipses. Total eclipses are centred around the cardinal points (marked by the red lines); Annular eclipses are centred around the intercardinal points (marked by the blue lines); and Hybrid eclipses are between these two regions.
