# Supplementary material for: Eclipse Prediction on the Ancient Greek Astronomical Calculating Machine Known as the Antikythera Mechanism
Source: PLoS One. 2014 Jul 30;9(7):e103275. doi: 10.1371/journal.pone.0103275 (PMC4116162; doi:10.1371/journal.pone.0103275)
Supplement: Tables S1 — The observed index letter groups and corresponding glyphs. (PDF) [file pone.0103275.s022.pdf]

L.9				L.18					L.29					L.36				
N_1	Λ_2	B_1	Φ_2	Z_1	Θ_1	Σ_2	P_1	X_1	2	Π_2	K_1	Z_2	Φ_1	T_1	H_2	Θ_1	P_2	Ψ_2
**55**	**154**	**8**	**201**	**25**	**31**	**184**	**72**	**90**	**213**	**172**	**37**	**125**	**84**	**78**	**131**	**31**	**178**	**207**
Σ, Η	Η	Σ, Η	Η	Η	Η	Σ, Η	Η	Σ, Η	Η	Σ, Η	Σ, Η	Σ, Η	Σ, Η	Η	Σ, Η	Η	Σ, Η	Η

Table S1 | The observed index letter groups and corresponding glyphs. Where the glyph is missing, it has been reconstructed according to EYM. **Red** = observed; **Blue** = reconstructed. One feature that all these glyphs have in common is that they index solar EPs. Less than half also index lunar EPs. So these Index Letter Groups must refer to solar EPs and the corresponding lines of inscription describe shared characteristics of solar eclipses.
