# Supplementary material for: Eclipse Prediction on the Ancient Greek Astronomical Calculating Machine Known as the Antikythera Mechanism
Source: PLoS One. 2014 Jul 30;9(7):e103275. doi: 10.1371/journal.pone.0103275 (PMC4116162; doi:10.1371/journal.pone.0103275)
Supplement: Tables S2 — The positions of the observed glyphs on the Saros Dial. (PDF) [file pone.0103275.s023.pdf]

First Node Lunar glyphs		Second Node Lunar glyphs		First Node Solar glyphs		Second Node Solar glyphs	
Month	*EYu*	*Month*	*EYu*	*Month*	*EYu*	*Month*	*EYu*
26	**75**	20	**293**	13	**46**	78	**286**
120	**79**	67	**295**	25	**56**	125	**288**
131	**51**	79	**305**	72	**58**	137	**298**
178	**53**	114	**297**	119	**60**	172	**290**
190	**63**	125	**269**	131	**70**	184	**300**
		137	**279**	178	**72**		
Mean	**64.2**	*Mean*	**289.7**	*Mean*	**60.3**	*Mean*	**292.4**
Min	51	*Min*	269	*Min*	46	*Min*	286
Max	79	*Max*	305	*Max*	72	*Max*	300
Range	28	*Range*	36	*Range*	26	*Range*	14

Table S2 | The positions of the observed glyphs on the Saros Dial. The glyphs are measured in EYu relative to the start of the Eclipse Year. A first approximation to the positions of the node points can be obtained by averaging the positions in the eclipse year of the observed glyphs, with lunar glyphs preferred because of the asymmetry of solar glyphs. The mean position of the observed lunar glyphs near the first node point is 64.2 EYu from the start of the eclipse year and near the second node point is 289.7 EYu. As described in the main text, 66 EYu and 289 EYu are adopted for EYM.
