# Supplementary material for: Eclipse Prediction on the Ancient Greek Astronomical Calculating Machine Known as the Antikythera Mechanism
Source: PLoS One. 2014 Jul 30;9(7):e103275. doi: 10.1371/journal.pone.0103275 (PMC4116162; doi:10.1371/journal.pone.0103275)
Supplement: Tables S3 — Eclipse times in the glyphs. (PDF) [file pone.0103275.s024.pdf]

A

	CERTAIN / VERY LIKELY				***UNCERTAIN***				
Month	*Certain Lunar*	*Hrs mech*	*Certain Solar*	*Hrs mech*	*Uncertn Lunar*	*Hrs mech*	*Uncertain solar*	*Hrs Mech*	*Comment*
13							Night?, 1 or 4	1, 4, 13, 16	$\mathrm{N}^{\backslash\mathrm{Y}}$ *cannot be excluded. Time* Δ *or* A.
20	Night, 6	18							
25			Day, 6	6					
26	Day, 7	7							
67									*No time information*
72			Night, 2	14					$\mathrm{N}^{\backslash\mathrm{Y}}$ *very likely.* H *in Nature 2008, but* B *likely.*
78			Day, 1	1					*Wrong in Nature 2008. Day 1 almost certain.*
79	Day, 10	10							
114	Day, 12	12							
119			Night, 10	22					*Probably no text after* I.
120					12?	12?			*Probably* $\mathrm{H}^{\backslash\mathrm{M}}$*. Time uncertain—possibly* IB.
125	Day, 2	2	Day, 3	3					H *in Nature 2008.* B very *likely— compare 137*
131	Night, 2	14	Night, 9	21					
137	Day, 5	5	Day, 12	12					
172	Night, 6	18	Day, 12	12					*Lunar wrong in Nature 2008:* ς *is clear*
178	Night, 9	21	Day, 9	9					
184	Day, 4	4	Day, 1	1					
190	Day, 9	9							

B

**LUNAR**		**SOLAR**	
Month	*24-hour*	*Month*	*24-hour*
20	**18**	25	**6**
26	**7**	72	**14**
79	**10**	78	**1**
114	**12**	119	**22**
125	**2**	125	**3**
131	**14**	131	**21**
137	**5**	137	**12**
172	**18**	172	**12**
178	**21**	178	**9**
184	**4**	184	**1**
190	**9**		

Table S3 | Eclipse times in the glyphs. (**A**) Tabulation of the certain / uncertain eclipse time data in the glyphs. (**B)** Eclipse times that are regarded as certain / likely.
