# Supplementary material for: Eclipse Prediction on the Ancient Greek Astronomical Calculating Machine Known as the Antikythera Mechanism
Source: PLoS One. 2014 Jul 30;9(7):e103275. doi: 10.1371/journal.pone.0103275 (PMC4116162; doi:10.1371/journal.pone.0103275)
Supplement: Tables S4 — Comparison between eclipse times one Saros apart. (PDF) [file pone.0103275.s025.pdf]

Time Shift		Lunar Eclipses	Actual	Exel.	AM	Error	Solar Eclipses	Actual	Exel.	AM	Error
Saroi	*Exel.*	*Date*	*Hrs UT*	*Hrs*	*Hrs UT*	*Hrs*	*Date*	*Hrs UT*	*Hrs*	*Hrs UT*	*Hrs*
**+ 0 Sa**		**-204 Dec-05**	**07:49**	0	07:49	**00:00**	**-204 Jun-25**	**13:58**	0	13:58	**00:00**
**+ 1 Sa**		**-186 Dec-16**	**16:46**	+8	15:49	**00:57**	**-186 Jul-06**	**21:29**	+8	21:58	**-29:00**
**+ 2 Sa**		**-168 Dec-27**	**01:40**	+16	23:49	**01:51**	**-168 Jul-17**	**05:05**	+16	05:58	**-53:00**
**+ 3 Sa**	**+1 Ex**	**-149 Jan-07**	**10:30**	0	07:49	**02:41**	**-150 Jul-28**	**12:47**	0	13:58	**-01:11**
**+ 4 Sa**		**-131 Jan-17**	**19:15**	+8	15:49	**03:27**	**-132 Aug-07**	**20:36**	+8	21:58	**-01:22**
**+ 5 Sa**		**-113 Jan-29**	**03:52**	+16	23:49	**04:03**	**-114 Aug-19**	**04:34**	+16	05:58	**-01:24**
**+ 6 Sa**	**+2 Ex**	**-95 Feb-08**	**12:23**	0	07:49	**04:34**	**-96 Aug-29**	**12:38**	0	13:58	**-01:20**
**+ 7 Sa**		**-77 Feb-19**	**20:45**	+8	15:49	**04:56**	**-78 Sep-09**	**20:51**	+8	21:58	**-01:07**
**+ 8 Sa**		**-59 Mar-02**	**04:58**	+16	23:49	**05:09**	**-60 Sep-20**	**05:13**	+16	05:58	**-00:45**
**+ 9 Sa**	**+3 Ex**	**-41 Mar-13**	**13:02**	0	07:49	**05:13**	**-42 Oct-01**	**13:42**	0	13:58	**-00:16**

Table S4 | Comparison between eclipse times one Saros apart. These are derived from the NASA/GSFC data [14] and the calculated eclipse time using the Exeligmos Dial adjustment. The error in the calculated time is shown in red. Dates are given throughout this study using the normal convention that "-204" refers to "205 BC".
