# Supplementary material for: Eclipse Prediction on the Ancient Greek Astronomical Calculating Machine Known as the Antikythera Mechanism
Source: PLoS One. 2014 Jul 30;9(7):e103275. doi: 10.1371/journal.pone.0103275 (PMC4116162; doi:10.1371/journal.pone.0103275)
Supplement: Tables S5 — Astronomical parameters at possible start dates for the Saros Dial. (PDF) [file pone.0103275.s026.pdf]

Date	*Sun at NM_0 to desc. node*	*% of min diam. Moon at FM_1*	*Longitude mean Sun at FM_1*
Model Inputs	***53.27°***	***100.00%***	***46.53°***
-240-05-05 15:53	54.12°	100.05%	25.26°
-222-05-16 23:23	54.59°	100.06%	36.01°
**-204-05-12 13:21**	**55.06°**	**100.09%**	**46.75°**
-186-05-23 19:45	55.54°	100.15%	57.50°
-168-06-03 02:08	56.01°	100.22%	68.24°
-048-01-01 02:48	54.40°	100.13%	277.38°
-030-01-11 10:43	54.82°	100.08%	288.19°
-012-01-22 18:32	55.24°	100.05%	298.99°

Table S5 | Astronomical parameters at possible start dates for the Saros Dial. The top row shows the parameters derived from EYM and ZZM.
