# Supplementary material for: Eclipse Prediction on the Ancient Greek Astronomical Calculating Machine Known as the Antikythera Mechanism
Source: PLoS One. 2014 Jul 30;9(7):e103275. doi: 10.1371/journal.pone.0103275 (PMC4116162; doi:10.1371/journal.pone.0103275)
Supplement: Tables S6 — Possible synchronizing eclipses and times. (PDF) [file pone.0103275.s027.pdf]

A

		FULL MOONS			NEW MOONS		
Mnth	*Year*	*Date*	*Time UT*	*Ecl*	*Date*	*Time UT*	*Ecl*
**-48**		Jun-24	22:10		Jul-09	22:11	
**-47**		Jul-24	05:27		Aug-08	13:03	
**-46**		~~Aug-22~~	14:42	**n**	~~Sep-07~~	03:22	**A**
**-45**		~~Sep-21~~	02:38	**n**	Oct-06	16:43	
**-44**		Oct-20	17:29		Nov-05	04:54	
**-43**		Nov-19	10:52		Dec-04	16:06	
**-42**		Dec-19	05:55		Jan-03	02:40	
**-41**	-207	Jan-18	01:16		Feb-01	12:56	
**-40**		**Feb-16**	**19:14**	**t**	~~Mar-02~~	23:07	**T**
**-39**		Mar-18	10:27		Apr-01	09:30	
**-38**		Apr-16	22:26		Apr-30	20:32	
**-37**		May-16	07:40		May-30	08:47	
**-36**		Jun-14	15:14		Jun-28	22:46	
**-35**		Jul-13	22:22		Jul-28	14:28	
**-34**		~~Aug-12~~	06:07	**t**	~~Aug-27~~	07:14	**A**
**-33**		Sep-10	15:14		Sep-25	23:54	
**-32**		Oct-10	02:12		Oct-25	15:29	
**-31**		Nov-08	15:23		Nov-24	05:30	
**-30**		Dec-08	07:00		Dec-23	18:00	
**-29**		Jan-07	00:44		~~Jan-22~~	05:06	**P**
**-28**	-206	**Feb-05**	**19:26**	**t**	Feb-20	14:52	
**-27**		Mar-07	13:22		Mar-21	23:33	
**-26**		Apr-06	05:07		Apr-20	07:46	
**-25**		May-05	18:13		May-19	16:32	
**-24**		Jun-04	05:02		Jun-18	02:57	
**-23**		Jul-03	14:20		**Jul-17**	**15:50**	**P**
**-22**		**Aug-01**	**22:57**	**t**	Aug-16	07:19	
**-21**		Aug-31	07:33		Sep-15	00:44	
**-20**		Sep-29	16:42		Oct-14	18:54	
**-19**		Oct-29	03:00		Nov-13	12:38	
**-18**		Nov-27	15:00		Dec-13	04:58	
**-17**		Dec-27	05:00		~~Jan-11~~	19:11	**A**
**-16**	-205	~~Jan-25~~	20:44	**n**	Feb-10	06:54	
**-15**		Feb-24	13:24		Mar-11	16:15	
**-14**		Mar-26	05:58		Apr-09	23:53	
**-13**		Apr-24	21:40		May-09	06:52	
**-12**		May-24	12:04		Jun-07	14:24	
**-11**		Jun-23	01:02		~~Jul-06~~	23:32	**H**
**-10**		~~Jul-22~~	12:35	**n**	Aug-05	11:05	
**-9**		Aug-20	22:59		Sep-04	01:22	
**-8**		Sep-19	08:43		Oct-03	18:17	
**-7**		Oct-18	18:30		Nov-02	13:10	
**-6**		Nov-17	04:57		Dec-02	08:40	
**-5**		~~Dec-16~~	16:23	**n**	~~Jan-01~~	03:00	**A**
**-4**	-204	Jan-15	04:47		Jan-30	18:38	
**-3**		Feb-13	17:56		Feb-29	06:58	
**-2**		Mar-14	07:44		Mar-29	16:26	
**-1**		Apr-12	22:15		Apr-28	00:01	
**0**		May-12	13:21		May-27	06:52	

B

**Lunar**	**ZZM cal ecl**	**ZZM cal ecl**	**ZZM cal ecl**	**ZZM cal ecl**	**ZZM cal ecl**	**Eclipse**	**Error Range**		
Month	*mech hrs*	*local hrs*	*UT@ - 1.9 hrs*	*UT@ - 1.3 hrs*	*UT@ - 1.0 hrs*	*UT hrs*	*- 1.9 hrs*	*- 1.3 hrs*	*- 1.0 hrs*
-40	14.27	20.27	18.37	18.97	19.27	**19.23**	-0.86	-0.26	0.04
-28	17.1	23.1	21.2	21.8	22.1	**19.43**	1.77	2.37	2.67
-22	15.62	21.62	19.72	20.32	20.62	**22.95**	-3.23	-2.63	-2.33
**Solar**	**ZZM cal ecl**	**ZZM cal ecl**	**ZZM cal ecl**	**ZZM cal ecl**	**ZZM cal ecl**	**Eclipse**	**Error Range**		
Month	*mech hrs*	*local hrs*	*UT@ - 1.9 hrs*	*UT@ - 1.3 hrs*	*UT@ - 1.0 hrs*	*UT hrs*	*- 1.9 hrs*	*- 1.3 hrs*	*- 1.0 hrs*
-23	11.21	17.21	15.31	15.91	16.21	**15.83**	-0.52	0.08	0.38

Table S6 | Possible synchronizing eclipses and times.

(**A**) Eclipses up to 48 months before -204-05-12 [14]. Each row represents a synodic month, with the bottom row marked in blue being the first month of the Saros Dial. They are organized so that Full Moon occurs before New Moon in each month, just as they do on the Saros Dial where the First Crescent Moon marks the start of the month. Lunar eclipses: n = penumbral; p = partial; t = total. Solar Eclipses: P = partial; T = total; A = annular; H = hybrid. A blue box marks lunar eclipses and an orange box solar eclipses, which were visible from the ancient Greek empire. A cross marks eclipses that were not observable [14].

(**B**) Hours required by the model for calibrating eclipse times vs actual hours for eclipses in the Saros scheme starting -204 May-12. Local time is taken as being in the range UT + 1.0 hours (Sicily) to UT + 1.9 hours (Rhodes), with an intermediate possibility of UT + 1.3 hours (Epiros).
