# Supplementary material for: Eclipse Prediction on the Ancient Greek Astronomical Calculating Machine Known as the Antikythera Mechanism
Source: PLoS One. 2014 Jul 30;9(7):e103275. doi: 10.1371/journal.pone.0103275 (PMC4116162; doi:10.1371/journal.pone.0103275)
Supplement: Note S1 — Eclipses & Predictions. (PDF) [file pone.0103275.s028.pdf]

Note S1: Eclipses & Predictions

The Moon's Orbit

The first part of this note gives reference information and definitions about eclipses [14], much of which would have been familiar to ancient Greek astronomers, though not necessarily to contemporary scientists. It is the basic information that is needed to understand this research paper. This part can be skipped by those already familiar with these ideas. The last part gives the parameters of the basic cycles that underlie the Antikythera Mechanism.

The astronomy is viewed in a geocentric frame-of-reference, as used in ancient Greece. The Moon's basic orbital cycle against the stars is the *sidereal month* (mean period, p_{sid} = 27.32 days). The term *syzygy* refers collectively to Full Moon and New Moon. The Moon's cycle from syzygy to syzygy is the *synodic* or *lunar month* (p_{syn} = 29.53 days). Eclipses do not happen every month because the Moon's orbit is tilted at 5.1° to the plane of the Sun's orbit round the Earth (in a geocentric frame), called the *ecliptic*. The *nodes* of the Moon are the points where the Moon's orbit crosses the ecliptic. The node, where the Moon moves up from South to North, is the *ascending node* and when it moves down from North to South it is the *descending node*. The *Line of Nodes* joins the Moon's two nodes: it precesses in the opposite direction to the Sun through the zodiac in a period of about 18.6 years. The Moon's cycle from a node back to the same node is the *draconitic month* (p_{drac} = 27.21 days). Eclipses occur when the Sun is close enough to one of the Moon's nodes at syzygy. If the Moon is not close enough to a node at New Moon, then the shadow of the Moon misses the Earth and there is no eclipse. Similarly, if the Moon is not close enough to a node at Full Moon, then the shadow of the Earth misses the Moon and there is no eclipse. The points when the Sun coincides with the nodes of the Moon are the *Ascending Node Point* (ANP) and *Descending Node Point* (DNP): collectively, the *node points*. The Sun moves from one node point back to the same node point in a period less than a year, called an *eclipse year* of about 346.6 days: each node point is about 173.3 days from the other. During each eclipse year, eclipses can only occur in *eclipse seasons* when the Sun is near one of the two node points. Lunar eclipses are visible throughout the hemisphere where it is night. The visibility of solar eclipses is critically dependent on the location of the observer and is affected by *North-South asymmetry*, caused by *parallax*: for an observer in the Northern hemisphere, a solar eclipse must be much closer to the node to be visible if it is South of the node than if it is North of the node. The Moon's *anomaly* refers to its variable motion, caused by its elliptical orbit. The cycle of the Moon from *lunar apogee*, its slowest motion against the stars, back to apogee is the *anomalistic month* (p_{anom} = 27.55 days).

Eclipse prediction cycles

Eclipse prediction cycles are periods when a whole number of synodic months is equal to a whole number of half draconitic months. When this is also close to a whole number of anomalistic months, then the appearance of the repeat eclipse is very similar. There are a variety of different eclipse cycles. Two of the shortest are the 41-lunar month *Hepton* (six 6-month periods + a 5-month period) and the 47-lunar month *Octon* (seven 6-month periods + a 5-month period) [14]. These work because:

$$41\ p_{syn} = 44.493\ p_{drac} \approx 44.5\ p_{drac}$$

$$47\ p_{syn} = 51.004\ p_{drac} \approx 51.0\ p_{drac}$$

The primary cycle used in ancient Babylonian and Greek astronomy, was the very accurate 223-lunar month *Saros Cycle*, known from at least the seventh century BC in Babylonian astronomy [8]:

$$223\ p_{syn} \approx 242\ p_{drac} \approx 239\ p_{anom}$$

The Saros Cycle is a chance resonance between three orbital periods of the Moon, which is fundamental to the design of the Antikythera Mechanism [1]. The first part of the equation explains its effectiveness as an eclipse prediction cycle: if there is an eclipse in a particular month, then there will

be a repeat eclipse after 223 lunar months. Figure S1 shows the geographical pattern of a sequence of such repeats.

Eclipse years & eclipse seasons

Eclipses occur when a syzygy happens sufficiently close to either the Moon's ascending node or descending node. In other words, when the Sun is sufficiently close to one of the Moon's nodes at syzygy. The Sun moves from one node point back to the same node point in a period less than a year, called an *eclipse year* of about 346.6 days—each node point is about 173.3 days from the other. During each eclipse year, there are two periods during which eclipses can occur: when the Sun is near either the ANP or the DNP. These are called *eclipse seasons*. The eclipse year can be regarded as the *beat cycle* between the synodic month and the draconitic month. Since there are 223 synodic and 242 draconitic months in the Saros period, the eclipse year has 242 - 223 = 19 cycles in the Saros period, since the frequency of a beat cycle is the difference in the frequencies of the underlying cycles. So, in each Saros period there are 19 eclipse years with 38 eclipse seasons. An eclipse season lasts for a period of approximately one lunar month, centred around the node points. Outside these periods, the Sun is too far from a lunar node for an eclipse to occur. Generally, there is one lunar eclipse and one solar eclipse in each eclipse season. It is possible to have two lunar eclipses or two solar eclipses in a single eclipse season. These are at the outside limit of distance from node for an eclipse to occur. Such lunar eclipses are nearly always both penumbral; and such solar eclipses are always both small partial eclipses.

North & South of the node

Solar eclipses occur when both Sun and Moon are near the same node. If the Sun is *before* the descending node or *after* the ascending node, then the Moon is *North* of the node. If the Sun is *after* the descending node or *before* the ascending node, then the Moon is *South* of the node.

Lunar eclipses happen when the Sun is close to one node and the Moon is close to the opposite node. If the Sun is *before* the descending node or *after* the ascending node, then the Moon is *before* the ascending node or *after* the descending node and so is *South* of the node. Similarly, if the Sun is *after* the ascending node or *before* the descending node then the Moon is *North* of the node.

An eclipse is described as being *North* or *South* of the node according to whether the Moon at the time of the eclipse is North or South of the node.

For solar eclipses, *gamma* is the distance of the shadow axis from Earth's centre at greatest eclipse, measured in units of Earth's equatorial radius [14]. It is *+ve* when the shadow axis is North of the Earth's centre; and *-ve* when it is South. For solar eclipses *North* of the node, gamma is *+ve*.; and *South* of the node it is *-ve*.

For lunar eclipses, *gamma* is the distance from the centre of the shadow cone axis to the centre of the Moon, measured in units of Earth's equatorial radius. It is *+ve* when the shadow axis is *South* of the Moon's centre; and *-ve* when it is *North*. For lunar eclipses *North* of the node, gamma is *+ve*; and *South* of the node it is *-ve*.

For all eclipses: +ve gamma is equivalent to North of the node and –ve gamma to South.

Distance from node, ecliptic latitude and gamma

Ecliptic coordinates will be measured in a geocentric frame of reference. At an eclipse, the distance of the Moon from the node (whether measured in degrees or days) is closely related to its ecliptic latitude and to gamma. This relationship is not simple because of the elliptic orbit of the Moon and its variable motion.

For a solar eclipse, the position of the centre of the Moon's shadow on the Earth is created by geometric projection. In general, the further North the Moon is at eclipse in terms of ecliptic latitude, the larger positive gamma the eclipse will have; and similarly for South. In other words, the Moon's

ecliptic latitude is reflected in gamma. For a lunar eclipse, the Moon's ecliptic latitude is also clearly related to gamma.

Visibility of eclipses

The locations of visibility of eclipses are very different for lunar and solar eclipses. Lunar eclipses are the shadow of the Earth on the Full Moon and this can be seen from anywhere on the Earth, where it is sufficiently dark—in other words, the hemisphere of the Earth where it is night. Lunar eclipses are classified as *total* (t), *partial* (p) or *penumbral* (n), according to whether the Earth's umbral shadow covers the whole of the Moon, part of the Moon or whether the Moon only falls within the penumbral shadow of the Earth. Solar eclipses are the shadow of the Moon on the Earth and this only covers a fraction of the Earth, since the Moon is much smaller than the Earth. The path of the umbral shadow is called the *eclipse path*. An observer needs to be within this eclipse path to see a total eclipse. Outside the eclipse path, an observer who is sufficiently close will see a partial eclipse. Solar eclipses are classified as *Total* (T), *Annular* (A), *Hybrid* (H) or *Partial* (P). An Annular eclipse occurs when the angular diameter of the Moon is smaller than that of the Sun: at maximum eclipse, an annular ring can be seen surrounding the Moon. A Hybrid eclipse is an eclipse which is Total for part of its eclipse path and Annular for other parts. Solar eclipses can only be seen during daylight at specific geographical locations that are close enough to the eclipse path. Typical eclipse maps can be seen in Figure S2.

Occurrence of solar eclipses according to distance from node

From NASA's Solar Eclipse website [14]:

"If Full Moon takes place within about 17° of a node, then a lunar eclipse will be visible from a portion of Earth. The actual value ranges from 15.3° to 17.1° because of the eccentricity of the Moon's (and Earth's) orbit."

"If New Moon takes place within about 17° of a node, then a solar eclipse will be visible from some location on Earth. The actual value ranges from 15.39° to 18.59° because of the eccentricity of the Moon's (and Earth's) orbit."

These limits take no account of the location of the observer—they simply indicate that an eclipse will occur, which is observable somewhere on Earth. As will be seen, the Antikythera Mechanism is designed to give information about the visibility of eclipses from a specific location, for which it was designed. So it is necessary to discuss the observer's location.

Parallax and solar eclipses

When an eclipse is North of the node, its path of totality will tend to be mostly in the Northern hemisphere and vice versa for eclipses South of the node. The relationship is not exact because all the bodies are moving relative to each other and the Earth spins on its axis— total solar eclipse paths can be very varied in shape and direction. Another factor is the tilt of the Earth's axis, which means that the effect depends on the time of year: at the equinoxes, when the Sun is directly overhead at the equator, a solar eclipse North of the node will indeed project primarily onto the Northern hemisphere; at the winter solstice, when the axis of the Earth is tilted away from the Sun, an eclipse North of the node may be seen as total in the Southern hemisphere. For a northern observer, an eclipse South of the node will only be observable if it is not too far South. This is simply the effect of parallax, whereby a close object (the Moon) changes its relationship with a distant object (the Sun), when the observer moves position. Figure S2 (**B**) shows a total solar eclipse, which was too far South of the node to be visible, even as a partial eclipse, from anywhere in the ancient Greek empire.

Babylonian eclipse prediction

The Saros cycle and schemes of eclipse prediction based on it are first known in the astronomy from Ancient Mesopotamia in the seventh century BC [8]. Astronomical observations were systematically recorded from at least the eighth century BC and methods were developed for predicting the

occurrence of eclipses. By the third century BC a lunar theory had been developed that enabled the calculation of the times and magnitudes of eclipses [8].

Though it is technically possible to have two lunar eclipses in a single eclipse season [14], in Babylonian prediction schemes only one eclipse of each type was ever included for an eclipse season. Strictly speaking, the Babylonian schemes predict *eclipse possibilities* (EPs) [8], not eclipses, since a lunar eclipse will only be visible when it occurs at night and a solar eclipse will only be visible during the day from the right location—so a prediction cannot guarantee that an eclipse will be observed. The same is true for the Antikythera Mechanism, though EPs will often be referred to here (less accurately) as eclipse predictions.

With 38 EPs in a Saros period of 223 lunar months, a lunar or solar EP will happen on average every $^{223}/_{38}$ = 5.87 lunar months. An eclipse can only occur at syzygy. So the EPs can only happen at intervals of a whole number of lunar months—either 5 months or much more frequently 6 months. The mathematical language of Excel, where "*" means multiplication, will be used in these notes.

From the Saros cycle:

$6*p_{syn} = 6*(242/223)*p_{drac} = 6.511*p_{drac}$

6 lunar months is just over 6½ draconitic months: if the Moon is before the node at a solar eclipse, then it will move towards the other node (or past it) after six months for another eclipse.

Take the situation where the Moon is at a maximum distance before the node for an eclipse to occur. Each six-month repeat takes the Moon towards the node and subsequently past it. This process of eclipse repeats can only go on for seven periods of six months before the syzygy is too far from the node:

$42*p_{syn} = 42*(242/223)*p_{drac} = 45.578*p_{drac}$

The Moon is now too far from the node for the process to go for another six months, so a five month period is needed to bring the Moon back to near its initial position relative to the node:

$5*p_{syn} = 6*(242/223)*p_{drac} = 5.426*p_{drac}$

$(42 + 5)* p_{syn} = 47*p_{syn} = 47*(242/223)*p_{drac} = 51.004*p_{drac}$

This eclipse period is called an *Octon* and is denoted by "8-", where the "8" refers to the eight eclipse repeats separated by seven six-month gaps and the "-" refers to the five-month gap. The Moon is now just beyond its starting point relative to the node. Each six-month period will move it relative to the node and the process can only go on for another six six-month periods before another 5-month period is needed to bring it back:

$41*p_{syn} = 41*(242/223)*p_{drac} = 44.493*p_{drac}$

This period is called a *Hepton* and is denoted by "7-". It consists of seven eclipses separated by six-month gaps, followed by a five-month gap.

Continuing in this way, the Saros period can be sequenced as: *Octon-Hepton-Octon-Hepton-Octon* or 8-7-8-7-8-. Essentially, the scheme breaks down the Saros period into 38 predictions of EPs in as even a way as possible [8]. The same can be done for lunar EPs. The basic Babylonian eclipse prediction scheme includes 38 lunar EPs and 38 solar EPs. A Saros period can start anywhere within the cycle described, not necessarily at the start of an Octon.

Metonic & Saros cycles and associated periods

This section defines periods and rotation rates for the parameters of the Antikythera Mechanism, based on the Metonic and Saros cycles [1]. Let ω indicate rotations and p indicate periods for the relevant astronomical body. All parameters refer to mean motions. "y" stands for "year"; "sid" for "sidereal month"; "syn" for "synodic month"; "drac" for "draconitic month"; "anom" for "anomalistic month"; "N" for "Line of Nodes"; "A" for "Line of Apsides". All periods are in years and rotations in rotations per year. So $p_y = 1$. Sidereal and tropical parameters are not distinguished.

Metonic cycle: $19p_y = 254p_{sid} = 235p_{syn}$

Saros cycle: $223p_{syn} = 242p_{drac} = 239p_{anom}$

From the Metonic and Saros cycles:

Sidereal month: ω_{sid} = 254/19 rotations per year
p_{sid} = 19/254 years

Synodic month: ω_{syn} = 235/19 rotations per year
p_{syn} = 19/235 years

Anomalistic month: ω_{anom} = (239/223)x(235/19) rotations per year
p_{anom} = (223/239)p_{syn} = (223 x 19)/(239 x 235) years

Draconitic month: ω_{drac} = (242/223)x(235/19) rotations per year
p_{drac} = (223/242)p_{syn} = (223 x 19)/(242 x 235) years

The draconitic month is shorter than the sidereal month because of the rotation of the Line of Nodes. In other words:

Line of Nodes ω_N = ω_{drac} - ω_{sid} = (242 x 235)/(223 x 19) - 254/19
= (242 x 235 - 254 x 223)/(19 x 223)
= 228/(19 x 223) = 12/223 rotations per year
p_N = 223/12 years

The anomalistic month is longer than the sidereal month because of the rotation of the Line of Apsides. In other words:

Line of Apsides ω_A = ω_{sid} - ω_{anom} = 254/19 - (239 x 235)/(223 x 19)
= (254 x 223 - 239 x 235)/(19 x 223)
= 477/(19 x 223) = (9 x 53)/(19 x 223) rotations per year
p_A = (19 x 223)/(9 x 53) years

Longitude of solar apogee from Vernal Equinox: A_0 = 65.5°.

The *Full Moon Cycle* (FMC) is the cycle of the Moon's apparent diameter at Full Moon. It can be regarded as the beat cycle between the synodic and anomalistic months. So it has 239 - 223 = 16 cycles per Saros period.

Full Moon Cycle (FMC): p_{FMC} = 223/16 = 13.94 lunar months

Eclipses happen when the Sun is sufficiently close to one of the nodes of the Moon. For a lunar eclipse, the Sun and Moon are near opposite nodes; for a solar eclipse near the same node.

The *Eclipse Year* (EY) is the cycle of the Sun from one of the Moon's nodes back to the same node. Since the Moon's nodes precess backward through the zodiac in a cycle of around 18.6 years, the Eclipse Year is shorter than the sidereal year. It can be seen as the beat cycle between the synodic and draconitic cycles of the Moon. So it has 242 - 223 = 19 cycles per Saros period.

Eclipse year: p_{EY} = 223/19 = 11.74 lunar months
= (223/19)/(235/19) years = 223/235 years = 346.6 days

These figures are all based on mean motions of the Sun and the Line of Nodes. The Sun varies from its mean position by around 2.5° and the Line of Nodes by around 1.7° [14]. So in practice there are variations, but they are not large.
