# Supplementary material for: Eclipse Prediction on the Ancient Greek Astronomical Calculating Machine Known as the Antikythera Mechanism
Source: PLoS One. 2014 Jul 30;9(7):e103275. doi: 10.1371/journal.pone.0103275 (PMC4116162; doi:10.1371/journal.pone.0103275)
Supplement: Note S2 — Eclipse Inscriptions. (PDF) [file pone.0103275.s029.pdf]

Note S2: Eclipse Inscriptions

Previous publication of inscriptions round the Saros Dial

Inscriptions round the Saros Dial were transcribed and read by 1974 [6], though the function of the dial was not understood and only the surface inscriptions in Fragment A were available. Though some of the glyphs were illustrated in 1959 [20], they were not understood as referring to eclipses:

> *"On the lower dial the letters and numbers seem to record "moon, so many hours; sun, so many hours"; we therefore suggest that this scale indicates the main lunar phenomena of phases and times of rising and setting."*

The discoveries that the Antikythera Mechanism predicted eclipses and that the glyphs were eclipse predictions were published in 2006 [1]. The X-ray CT data of 2005 revealed many new inscriptions, which are completely hidden inside the fragments (see main text Methods). These augmented inscriptions, were also published in 2006 [1], though a translation was not included in the publication. The additional inscriptions at the top of Figure S4 (**B**) are from X-ray CT of Fragment E and at the bottom from X-ray CT of Fragment F. It should be noted that in the right-hand transcription from 2006 the lines of text from Fragment E at top-left in blue and orange are one row too high. In other words, the start of lines 4 - 7 should be the start of lines 5 - 8. This must have arisen because of a misalignment of Fragment E relative to Fragment A.

New tracing of eclipse inscriptions

Figure S5 shows a new tracing of the eclipse inscriptions on the lower half of the back plate. This includes modifications to the publications of 1974 [6] and 2006 [1]. All of the lines of text are partial and hard to read, due to two millennia of corrosion and damage. The author traced the letters of the eclipse inscriptions directly from the data with knowledge of the Greek alphabet but virtually no knowledge of the Greek language. This process had the advantage of not imposing expectations of what "ought" to be there, but the disadvantage of overlooking combinations of Greek letters that would be obvious to an expert. Comparison of these tracings with previous publications [1], [6] meant that some modifications were made and the mismatch in lines 4 - 7 was noted. The inscriptions were then examined from these tracings and from the original data by Dr Charles Crowther (University of Oxford). This led to a comprehensive re-examination of the data and many further additions.

First the Index Letter Groups are examined and explained, then a detailed analysis of the content of the inscriptions is given.

Decipherment of Index Letter Groups

The index letters in the glyphs are critical for this study, so a detailed examination of the data is included in Figure S6. The index letters evidently index the inscriptions round the Saros Dial, since some of the lines of inscriptions make no sense as words and can only be sets of index letters. One such group of index letters, the bottom line of inscriptions in L. 36, has no space for more inscriptions underneath. So each group of index letters must index the lines of inscriptions above them. As will be shown, these lines of inscriptions describe shared characteristics of the eclipses indexed by the group of index letters. The way that these groups are selected and ordered was previously very perplexing. The main text describes how EYM solves these problems.

Texts, translations and analysis of the eclipse inscriptions

Figure 7 in the main text shows a new tracing of the eclipse inscriptions. The inscriptions were transcribed, translated and analyzed by Dr Charles Crowther, Centre for the Study of Ancient Documents and The Queen’s College, University of Oxford (2013). His analysis is quoted in full below.

Structure of inscriptions

Dr Charles Crowther:

The text falls into five sections, the first of which may be introductory, although the surviving letters are too few for a judgement of any security to be made. The remaining four sections follow a regular pattern that allows uncertainties and lacunae to be resolved from one to the next. Each starts with a direction indication marked against compass points for which wind names are used: from the North (5: ἀπὸ Βο̣[ρέου]); from the North-North-West (10: ἀπὸ Θραϊκί[ου]); from the West (19-20: [ἀπὸ Ζε]φύ̣[ρου]); from the South (20: ἀπὸ Νότου). (The use of Thraskias (Θρασκίας or, as here, Θρα̣κίας: North-North-West wind) suggests that a ten or twelve-point wind rose is being used for compass directions (cf. D'Arcy Wentworth Thompson, "The Greek Winds", The Classical Review 32, 49-56 for the Aristotelian origin of this). The Tower of the Winds in Athens, in contrast, shows eight winds only (CIG I, 518; SEG 33 199), omitting both Thraskias and Meses (NNE).) This indication appears to represent a starting point since it is followed in each section by a new clause, introduced by the connecting particle δέ, consisting of two verbs indicating rotation and termination (περιἵστανται δὲ καὶ καταλήγουσιν), the latter in turn completed by a direction qualification: towards the South-West (6-7: πρ[ὸς] Λίβα); towards the East (13-14, 25-26, 33: πρὸς Ἀπηλιώτην). The next element is a single adjective, in two cases a feminine nominative plural, qualifying the implicit subject of the verbs, in the other two a feminine accusative singular; the referent of the latter is unclear. Each section then concludes with an indication of colour (τὸ δὲ χρῶμα), followed by a group of index symbols. [CC]

Epigraphic notes on inscriptions

Lines 1-4 appear to belong to an introductory section, although too little survives for any reconstruction to be attempted. In the text I have indicated a lacuna of 10-15 letter spaces to the left of the surviving text, using the preserved beginning of line 5 as a guide and assuming an even left margin; it is possible, however, that the lettering may have extended further to the left; the figures should accordingly be read as minimum lengths for the lacunae.

Line 1: the first letter was previously read as pi.

Line 2: the surviving letter traces invite the restoration [μ]ι̣κραί ("small", feminine nominative plural adjective) but the context and referent are lost; the feminine accusative singular form μικράν occurs in line 34.

Line 3: the visible lacuna after INON suggests that this is the end of a section or sentence. The letters themselves seem to belong to a word ending -ινός, as in adjectives indicating time or seasonal markers (θερινός, summer; χειμερινός, winter; ἰσημερινός, equinoctial, and so on).

Line 4: the surviving letter trace has been read as omicron, but seems to be open rather than closed, and may instead be psi, or, perhaps, the composite omega and rho ὥρα symbol.

Lines 5-6: Βο[ρέου] seems an evident restoration and fits the lacuna perfectly. περιἵστανται is read almost completely in line 5 and assured by its appearance in ll. 10-11 and 30-31; [κατα]λήγουσι is restored securely from ll. 12, 24-25, and 32. Both verbs, περιἵσταμαι (LSJ s.v. περιίστημι B II. "come round", "revolve") and καταλήγω (LSJ s.v. καταλήγω, "leave off", "stop") make perfect sense in an eclipse description, although they appear not to be used in technical writing in this context.

Line 7: the letter traces in the middle of the line belong to a feminine nominative plural adjective, but are elusive; one level of the X-ray CT scan appears to show the outline of an omega followed by epsilon before the plural ending -αι, but these traces are evanescent at best at other levels and do not lead to an obvious restoration. If the apparent epsilon could be taken as sigma, which is similar in general shape, [μέ]σ̣αι might be restored, as in ll. 26-27, although this seems a little too short for the lacuna.

Lines 14-15: [μεγά]|λην has been restored by analogy with μικράν in l. 34.

Lines 19-20: phi is secure at the beginning of line 20; since words are regularly divided syllabically at line ends and there seems to be space only for five letters in the preceding line, the restoration [ἀπὸ Ζε]|φ̣ύ[ρου] seems inevitable; there are possible compatible traces of the left foot of alpha at the beginning of l. 19, and the foot and tip of the left branch of upsilon in l. 20.

L. 23: Νότον, the accusative singular form, seems to be used in an adverbial sense, as νοτόνδε, to indicate rotation "southwards", to resolve a possible ambiguity in the direction of movement from West to East.

LL. 26-27: the lacuna after the partial mu in l. 26 is sufficient for only one letter, so that the restoration μ̣[έ]|σαι, "medium", seems required. [CC]

Dating of inscriptions

The most accurate physical dating of the Antikythera Mechanism currently involves stylistic analysis of the letter forms in the inscriptions. This method of dating is frequently subject to debate, since experts disagree. A previous publication in 2006 [1] (Supplementary Notes 2 (Glyphs and Inscriptions)) included such an analysis from a leading epigrapher:

> *According to Charalambos Kritzas (Director Emeritus of the Epigraphic Museum, Athens) the style of the writing could date the inscriptions to the second half of the 2nd Century BC and the beginning of the 1st Century BC, with an uncertainty of about one generation (50 years). Dates around 150 BC to 100 BC are a plausible range. We give here a few examples of the epigraphic clues to the dating, but detailed analysis will be published elsewhere:*
>
> *Π* *pi has unequal legs - second half of 2nd century BC*
>
> *Σ* *sigma has the two lines not horizontal but at an angle - second half of 2nd century BC, beginning of 1st century BC*
>
> *Μ* *mu has the two lines not vertical but at an angle - second half of 2nd century BC. There is one Μ with vertical lines*
>
> *Υ* *upsilon has the vertical line short - second half of 2nd century BC*
>
> *Α* *alpha - just post Alexander*
>
> *Ζ* *zeta is written like I with long horizontal lines - 2nd century BC*
>
> *Ω* *omega and not like ω - 2nd century BC*
>
> *Β* *beta unequal upper circle, compared with the lower circle - old*
>
> *Ο* *omicron very small - old*
>
> *Θ* *theta has a short line in the middle, in one case a dot - 2nd century BC*
>
> *Φ* *phi is arc like - old*
>
> *Ξ* *xi middle line short - old*

Dr Charles Crowther:

Ch. Kritzas' valuable comments on letter forms are accurate at a general level, but comparanda are limited for writing of this size in stone inscriptions, and lettering on bronze would have required a different technique; a further difficulty for finding parallels is the uncertainty over the provenance of the device. Even so, some qualifications can be offered.

Dr. Kritzas notes that pi has unequal legs and places this form in the second half of the second century BC. Since pi in Classical and early to mid-Hellenistic stone inscriptions has a right vertical that is consistently, and often markedly, shorter than the left, Dr Kritzas' comment appears to be an ellipsis for "has unequal legs with the right slightly shorter than the left". This form of pi, with right vertical close in length to the left, is by no means limited to the second half of the second century or later: three fragmentary Corinthian inscriptions recording decrees, which must antedate the destruction of the city in 146 BC, all show the same form (J.H. Kent, Corinth VIII(iii) 46a; 46b with Corinth I-77.13; N.

Robertson, Hesperia 45, 1976, 253-266 [SEG XXVI 392]); these fragments (for example, Figure S7*) offer an interesting comparison because their lettering is quite small – around 5-6mm – and the inscribing style is reminiscent of lettering on bronze, which seems to have been the regular medium for texts of this kind in Hellenistic Corinth. Of the other letter forms discussed by Kritzas, zeta requires further comment: in the Antikythera inscriptions it has the regular Classical and early-mid Hellenistic form, with two horizontals joined by a central vertical, which was gradually superseded on stone inscriptions from the middle of the second century onwards by a form similar to Z with a diagonal stroke linking the lower and upper horizontals. The other letter forms of the Antikythera inscriptions are compatible with a broad later-third to later-second century dating range. It seems better, accordingly, to widen the palaeographical dating range for the Antikythera inscriptions to the end of the third to the beginning of the first century BC, with a preference for the earlier half of this period.*

In an important recent discussion R. Hannah, Time in Antiquity (London, 2008) [22], p. 31 with fn. 10, has suggested that the letter forms of the Antikythera mechanism bear "strong similarities to inscriptions from the second half of the second century BC". The inscriptions cited by Hannah are all taken from S.V Tracy's remarkable study Attic Letter-Cutters of 229 to 86 B.C [23]. (Berkeley, 1990); the Athenian palaeographical tradition throughout the Hellenistic period, however, is notably distinct and of limited value for the dating of inscriptions from other areas. Of the particular examples offered by Hannah, all but one differ both in general appearance and in specific letter forms from the Antikythera texts; EM 7751 [IG II2 957] (the closest, and earliest, parallel, but pi has full or almost full length right vertical, and upsilon curved branches); Cutter of IG II2 937 (a very formal hand with quite slender letters; alpha with broken cross bar); EM 7670 [IG II2 1134] (very heavy serifs; alpha with curved or broken cross bar); EM 7667 [IG II2 1136] (pi with almost full-length right hasta; alpha with straight, slanting and curved cross bar; phi with double loop); the partial exception is Tracy's Cutter of FD III 2 no. 24, who is a Delphian rather than an Athenian stonecutter since the unpublished inscription cited by Tracy for his Athenian origin, EM 2857, is a fragment of a Delphian manumission document of conventional form; the general appearance and particular letter forms of this cutter's work belong to a distinctive Delphian style in evidence from the last quarter of the third century (for example, FD III 2, 19, archonship of Kallieros) onwards (cf. FD III 3, 383, of 180/79).

[CC]

Astronomical analysis of inscriptions

The grouping of the index letters means the eclipses indexed by each group should share common characteristics—as is implicit in the structure of the inscriptions. These characteristics are described repetitively for each group in a fixed order: *directions*, *magnitude*, *colour*. The characteristics are presumably intended as an "average" for that Index Letter Group: the user can then make adjustments, knowing that the order of the Index Letters in each group indicates descending order from North to South (main text, Figure 7).

Directions

The *directions* statements are the most problematic. When the inscriptions round the Saros Dial were first published in 1974 [6], language relating to directions were noticed:

> *"In the inscription on the lower back dial (fig. 37) lines 13-14 might be read ΠΡΟΣ ΑΠΗΔ[Ι]ΩΤΗΝ, "Towards the East (wind)"; similarly perhaps lines 16-17 may be some form of ΙΑΠΥ[Γ]Ο[Σ], "west- north-west (wind)" and line 6 some form of ΛΙΨ (genitive ΛΙΠΟΣ) "west-south-west (wind)." But what the point of these directional mentions may be I cannot guess."*

The idea appears to be that the directions refer to winds, with the implication that it was a meteorological inscription—particularly since the language used is that of wind directions. At this stage it was not realized that the inscriptions referred to eclipses. However, the idea that wind directions were intended has persisted. Whilst there is some literature that describes the meteorological effects of solar eclipses [24], even for a total eclipse the effects are generally agreed

to be minor, since an eclipse shadow cannot in general create sufficient thermal gradients to have large-scale wind effects (ignoring the *butterfly effect*!). It hardly seems worth taking space on the cramped plates of the Antikythera Mechanism to inscribe a prediction that there might be a mild breeze as a result of a total solar eclipse. For a small partial solar eclipse, the wind would be negligible.

The *directions* statements within each group are as follows:

L. 9 Group: *From the North, and they revolve and end towards the South-West.*

L. 18 Group: *From the North-North-West, and they revolve and end towards the East.*

L. 29 Group: *From the West, and they revolve southwards and end towards the East.*

L. 36 Group: *From the South, and they revolve and end towards the East.*

The Index Letter Groups are defined by the geometry of the eclipses. It seems far more likely that the directions relate to the geometry of the eclipse event, than to wind directions. This could be connected to the eclipse path, the direction of motion of the Moon or to the directions of obscuration during the eclipse. Though there appears to be some common characteristics of solar eclipse paths that are grouped by the Index Letter scheme (Figure S19), it is a difficult concept to pin down because eclipse paths are complicated and were impossible to calculate in the ancient world. Finally, eclipse paths do not "revolve" in the way described in the inscriptions. So this seems very unlikely. The inscriptions cannot be describing the direction of the Moon's motion during an eclipse, since this also does not "revolve" in the way described in the inscriptions: it is always from East to West.

It is most plausible that the directions statements refer to the directions of obscuration during an eclipse. The first question is what frame of reference should be used for defining these directions: horizontal, equatorial or ecliptic? Evidence from Babylonian cuneiform texts suggests that the Babylonian descriptions of eclipse obscuration directions are most likely to have used either equatorial or ecliptic co-ordinates—with ecliptic perhaps more likely [25]. This would certainly coincide with the ecliptic frame of reference generally adopted in the Antikythera Mechanism—for example, on the Zodiac Dial. The Index Letter Groups are also defined in terms of the North-South position of the eclipses relative to the ecliptic. The most probable frame of reference is therefore ecliptic, where the WE-axis is along the ecliptic and the SN-axis is normal to it.

According to its gamma, the solar eclipse in Figure S8 (**A**) would probably fit best as part of either *L. 18 Group—Quite close North of node* or *Conjectural Solar Group D: Close North of node* (Figure 7).

L.18 Group: *From the North-North-West, and they revolve and end towards the East.*

The phrase "West wind" generally means a wind coming from the West that is moving East. With this sort of convention, the direction of obscuration in Figure S8 (**A**) might be described as, *"From South-East, rotating towards South-West."* This is almost diametrically opposite to the L. 18 Group description. This pattern, whereby the Antikythera eclipse inscriptions often appear to give opposite directions, runs throughout the text. Returning to the "West wind" analogy, with a different convention it could more aptly be described as an "East wind" to define its direction of movement towards the East. The key question is: what are the conventions that define the directions of obscuration?

Define the *shadow line* as the line joining the centre of the eclipsing object (either the Moon or the Earth's shadow) with the centre of the eclipsed object (either Sun or Moon). To formalize the idea of a *direction of obscuration*, a *shadow vector* is required that is aligned with the shadow line and defines a direction. The vector is intended to point towards the *described* direction: in other words, it points towards the West if the direction is described as "West". There are four possibilities, visualized for the partial solar eclipse in Figure S8 (**A**): (**C**) the shadow vector always points from the centre of the eclipsing object to the centre of the eclipsed object (*Always Towards)*; (**D**) the shadow vector points from the centre of the eclipsing object to the centre of the eclipsed object until maximum eclipse, after which it points away (*Towards then Away)*; (**E**) the shadow vector points from the centre of the eclipsed object to the centre of the eclipsing object until maximum eclipse, after which it points away

(*Away then Towards)*; (**F**) the shadow vector always points from centre of the eclipsed object to the centre of the eclipsing object (*Always Away).* The directions in the inscriptions all apply to solar eclipses. They are now examined to determine which of these conditions applies.

Since the L. 18 directions end as *"...towards the East."*, options **D** and **F** can be eliminated. Three of the four directions in the Index Letter Groups end up as *"...towards the East"*, so these options can be eliminated more generally than just for this eclipse. Since the L. 18 directions start as "*From the North-North-West..."* option **E** can be eliminated. This means that option **C** is the only one that matches this eclipse. The match is not exact but it is close enough. Since all the initial directions in the text are in the S-W-N sector and the Moon travels from right to left relative to the Sun, option **C** is also the only choice that can work for more general eclipses. The convention that corresponds to *"Always Towards"* will be referred to as the *shadow vector convention*. It runs counter to the conventions, which are often assumed, that the direction should describe where the eclipsing object is coming from (the "West wind" convention) and that it should reflect the "direction of motion" of the eclipsing object as the eclipse comes to an end. It also appears to be the opposite of the conventions used for specifying directions of obscuration in Babylonian texts [25].

The conclusion is that the directions statements in the eclipse inscriptions refer to *directions of obscuration* based on an *ecliptic frame of reference*, using the *shadow vector convention*.

Eclipse magnitude

The *magnitude* statements within each group are as follows:

L. 9 Group: *medium(?)*

L. 18 Group: *large*

L. 29 Group: *medium*

L. 36 Group: *small*

For the characterization of the Index Letter Groups, see Figure 7 and Note S3. It might be expected that the *L. 29 Group: Nearly at node: North then South* would give eclipses of largest magnitude. If this described the magnitude of eclipses regardless of location, this would be true. However, the descriptions are clearly intended to reflect observations based on the location of the observer. It is far more likely that a total eclipse in the L. 18 Group would appear to be total for an observer at a geographic latitude that is reasonably far North (Figure 7). This is reflected in the description *"large"* in the L. 18 Group. It might be expected that the eclipses in the L. 9 Group: *Far North of node* would be described as *"small"*, but *medium* would be acceptable if the observer is sufficiently far North. Then the Conjectural Solar Group A: *Very far North of node* would presumably be described as *"small"*. Referring to the six Index letter groups in Figure 7 from *Very Far North of node* to *Close South of node,* a conjectural sequence for the eclipse magnitudes is the symmetrical pattern: *small,* ***medium****, large,* ***large****,* ***medium****,* ***small***, where bold indicates it was read from the observed text.

The description in the solar eclipse inscriptions are consistent with the idea that they are describing *eclipse magnitudes* from the location of an observer sufficiently far North geographically. There is probably not enough information here to determine the latitude of the intended use of the Mechanism any more closely than *"sufficiently far North"*.

Eclipse colour

The *colour* statements within each group are as follows:

L. 9 Group: *...and the colour is black...*

L.18 Group: *...and the colour is red...*

L. 29 Group: *...and the colour is black...*

L. 36 Group: *...and the colour is black...*

It is surprising to see the colours of solar eclipses included in the inscriptions. Historically, there is some literature describing the colours of solar eclipses [26], but the colour of a solar eclipse was rarely recorded in antiquity. Again, this is much more relevant for lunar eclipses. The L. 18 group is the only group that includes a colour "red" that is not "black". By comparison with *magnitude*, this may be because the L. 18 Group is most likely to include eclipses that are total for the location of the intended northerly user of the Mechanism. A total solar eclipse can look *"red"* due to light scatter [14].

Discussion of Index Letter Groups

It must be said that the solar Index Letter Groups are unlikely to reliably group solar eclipses with common *directions of obscuration*, common *magnitudes* or common *colours*. There is a serious problem for solar eclipses in grouping them by Index Letters that are defined simply by criteria related to North-South of the node point, as realized by the Index Letter Groups (main text and Note S3). The directions of obscuration and the magnitude for a solar eclipse depend critically on the location of the observer and the time of year. Comparisons between the solar eclipses in Figure S8 (**A**) and (**B**) illustrate these problems. Though these central eclipses have similar gamma and would probably be grouped by EYM in the same Index Letter Group, they are viewed from very different locations relative to the eclipse paths and this leads to contrasting *directions of obscuration*. Part of the reason for this is that the eclipse in Figure S8 (**A**) was observed at a time of year close to the vernal equinox, when the Sun was overhead at the equator, whereas the eclipse in Figure S8 (**B**) was observed much closer to the summer solstice, when the Earth's axis was pointed more towards the Sun in the northern hemisphere. The other part of the reason is that the gamma in (**A**) was that much larger than the gamma in (**B**), so the eclipse path was projected further north onto the globe. The directions that appear in the solar eclipse inscriptions can at best be regarded as a broad qualitative description. Lunar eclipses could be much more meaningfully grouped in this way, as is discussed in Note S3. The other aspect that is also clearly different is the apparent magnitudes of the eclipses in (**A**) and (**B**). They are both total eclipses, but appear to be of very different magnitude from the location of the observer.

It might be thought that all of this invalidates the idea of the *Index Letter Groups*. The root of the problem probably lies elsewhere: the *solar Index Letter Groups* were carried over by analogy with similar *lunar Index Letter Groups* and *lunar eclipse inscriptions*. The whole scheme works so much better for lunar as opposed to solar eclipses. Later, robust arguments will be put forward that the Antikythera Mechanism also included lost lunar eclipse inscriptions.
