# Supplementary material for: Eclipse Prediction on the Ancient Greek Astronomical Calculating Machine Known as the Antikythera Mechanism
Source: PLoS One. 2014 Jul 30;9(7):e103275. doi: 10.1371/journal.pone.0103275 (PMC4116162; doi:10.1371/journal.pone.0103275)
Supplement: Note S3 — Eclipse Year Model, EYM. (PDF) [file pone.0103275.s030.pdf]

Note S3: Eclipse Year Model, EYM

Stimulus for EYM

The model described in the main text is based on *eclipse years* (Note S1). It was originally stimulated by the wish to divide the Saros dial on the Antikythera Mechanism into 19 mean eclipse years on a spreadsheet. To enable this, the initial model divided each month into 19 units, with 223 units for each eclipse year. However this meant that half-units were needed for such parameters as the distance between the node points (111.5 units). So this was modified to a division of each mean lunar month into 38 divisions, which will be called *Eclipse Year units* or *EYu* for short. This enables the whole model to be calculated with integers alone.

In terms of the parameters of the Antikythera Mechanism (Note S1):

1 EYu = p_{syn}/38 = (19/235)/38 = 1/470 years

= 365.25/470 days = 0.777 days

Each eclipse year is 446 units and the distance between the two node points is 223 EYu.

The months on the Antikythera Mechanism start at first crescent Moon [1]. Calculating the actual timing of this relative to new Moon is difficult and varies from month to month [27]. In summary, the first crescent Moon is usually observed between one and two days after astronomical New Moon. For EYM it will be assumed that First Crescent Moon is at 2 EYu (1.55 days) after New Moon, though the exact timing does not affect the model.

Closeness to node; closeness to node point; ecliptic latitude

All calculations will be done in terms of mean motions. *Closeness to node* differs from *Closeness to node point*, because the nodes of the Moon are not stationary. Recall that in terms of the parameters of the Mechanism (Note S1), the rotation of the *Line of Nodes* is 12/223 rotations per year. For example, if a particular New Moon is 10 days before the DNP, then the node at this syzygy must be 10*(12/223) = 0.54 days after DNP, so that the Sun and lunar node will coincide at the DNP 10 days later. In other words, *Closeness to node* = 10.54 days at this New Moon. This can be generalized for both New Moons and Full Moons as follows:

Closeness to node in EYu = (1 + 12/223)**Closeness to node point* in EYu

= (235/223)**Closeness to node point* in EYu

This can be translated into degrees, so that:

Closeness to node° = (235/223)*(360/p_{year})**Closeness to node point* in days

= (235/223)*(360/p_{year})*(p_{year}/470)**Closeness to node point* in EYu

= (180/223)**Closeness to node point* in EYu

Closeness to node point can be seen as a surrogate measure for the *ecliptic latitude* of the Moon. If mean motions are assumed for all the bodies, then this can be calculated to a reasonable degree of approximation as:

Ecliptic latitude° ≈ *Closeness to node* in degrees*tan (5.1°)

= (180/223)*tan (5.1°)**Closeness to node point* in EYu

≈ 0.0720**Closeness to node point* in EYu

= 9/125**Closeness to node point* in EYu

≈ 1/14**Closeness to node point* in EYu

This is obviously an approximation, since the astronomical bodies move on elliptical orbits, not straight lines and the distance of the Moon is variable. Since all the measures are essentially related to each other by constant factors and EYM is concerned with relative (not absolute) closeness to

node at syzygies, EYM uses the concept of *Closeness to node Point* in EYu, except when calculations need to be made about *Closeness to node* in degrees.

Eclipse prediction by numbers

Data on the positions of the node points is included in Table S2. The definitions of EYM, including the limits for determining EPs, are in the main text. To check whether a particular syzygy should be included as a glyph in EYM is done simply by counting. The results are in Figure S10. It is easy to check that EYM is consistent with all the known glyph data, including the alphabetical sequence of index letters. Note that there are three extra-alphabetic symbols at the end of the scheme. There is evidence in L. 29 of the eclipse inscriptions of the first such symbol, which looks exactly like a "2" (Note S2). There is no direct evidence for the other two symbols, but they are here denoted by "3" and "4", though of course they would not have been at all likely to have had this form!

The breakdown of the Saros period into eclipse years, showing the generation of the glyphs by EYM, is illustrated in Figure S9.

Comparison of EYM with a previous model

The previous model [4] will be referred to as N2008. EYM is very similar in principle to N2008, but it uses simple arithmetic instead of far more advanced calculation. The pattern of lunar glyphs in EYM is composed of a complete Babylonian 38 EP set, with the characteristic Babylonian 8-7-8-7-8- pattern (albeit starting in the middle of a Hepton). Surprisingly, the pattern of solar glyphs is not a subset of this pattern. In EYM, the solar glyphs form the pattern: 8-8-8-7-7-, though the border between the last two '7-'s is blurred by the excluded solar EPs at months 190 and 195. This non-Babylonian distribution of the glyphs was already inherent in N2008 [4]: the five month gaps 8→13, 55→60, 102→107, 149→154 show that this model had three consecutive Octons, 13→60, 60→107, 107→154. This is not possible in the Babylonian scheme. This irregularity in N2008 does not appear to have been noticed before. The non-Babylonian scheme generated by EYM should not be of too much concern, since the Babylonian schemes are created to make the distribution of EPs as even as possible throughout the Saros period [8], whereas EYM is arguably based on a better principle for determining EPs—notably, *closeness to node*.

N2008 had the defect that it generated lunar glyphs in both Months 125 and 126 [4]. The authors justified exclusion of the second lunar glyph, by arguing that lunar eclipses in consecutive months are almost always penumbral [14] and lunar EPs were never included in consecutive months in the Babylonian schemes [8]. The current model has the same defect in the same month—as well as two additional months—and the same solution is adopted. This is the *consecutive month rule*.

The limits used for determining EPs in N2008 [4] were as follows:

> *"The sequence is based on asymmetrical elongation criteria as follows (where any number in the given range will produce the same generated sequence):*
>
> | | | |
> |---|---|---|
> | *Lunar* | *15.4° - 16.1°* | *for example, 16°* |
> | *Solar - North* | *15.4° - 16.1°* | *for example, 16°* |
> | *Solar - South* | *5.7° - 6.1°* | *for example, 6°* |
>
> *These compare with Ptolemy's figures in the second century AD for estimating eclipse likelihood by syzygy elongations from the closest node:*
>
> | | |
> |---|---|
> | *Lunar* | *12.2°* |
> | *Solar - North* | *17.7°* |
> | *Solar - South* | *8.4°"* |

The limits used in EYM are very similar. For comparison, the *Closeness to node* limits in EYM will be calculated. Recall the relationship between *Closeness to node* and *Closeness to node point*, described earlier. This means that 20 EYu *from node point* = $20*(235/223)*(p_{year}/470)*(360/p_{year})$ = $20*(360/470) = 16.1°$ *from node*. The limits are:

Lunar	20 EYu from node point	= 16.1° from node
Solar - North	20 EYu from node point	= 16.1° from node
Solar - South	7 EYu from node point	= 5.4° from node

Modern figures [14] should also be noted for comparison, though these take no account of the location of the observer:

Lunar	17.0° from node	*"The actual value ranges from 15.3° to 17.1° because of the eccentricity of the Moon's (and Earth's) orbit."*
Solar	17.0° from node	*"The actual value ranges from 15.39° to 18.59° because of the eccentricity of the Moon's (and Earth's) orbit."*

Notes on EYM and the solar Index Letter Groups

After realizing that the North-South division of the EPs was essential for understanding the structure of the Index Letter Groups, one of the main problems in reaching this model was the L. 29 Group, which includes glyphs that are North and South of the node.

The uncertain index letter Ψ_2 is the last of the L. 36 Group. If Ψ_2 did not exist, then the theory can easily accommodate this by changing the solar South limit from 7 to 6. This does not change the other glyphs, but it would mean that there was one fewer glyph and the index lettering of the last four glyphs would change. In other words, the identification of Ψ_2 is not essential for the theory: it is included since it does appear to be there.

EYM exactly generates the known Index Letter Groups, with one exception: Σ_2 is in the right L. 18 group but in the wrong place within the group. It is relatively easy to establish that this is not caused by the node points being in the wrong place: it can be proved that changing the positions of the node points cannot fix the problem.

For definitions, see Note S1. As described earlier, *Closeness to node point* in EYu is directly related to the *ecliptic latitude* of the Moon. This is also strongly related to *gamma*—the further North the Moon is in terms of ecliptic latitude, the further away from the Earth's centre will be the centre of the Moon's shadow on the Earth. The relationship is not entirely direct because of the elliptic orbits of the Moon and Sun: for a particular lunar longitude, the size of gamma will depend on the relative distances of Moon and Sun from the Earth.

Closeness to node point North or South is loosely related to the geographical latitude of the centre of the Moon's shadow on Earth—though this depends on the tilt of the Earth's axis, which depends on the time of year relative to the solstices and equinoxes. The eclipse times give rough information about the longitude of the eclipse path. In very broad terms, the Antikythera Mechanism's eclipse prediction scheme gives information about both longitude and latitude of the eclipse path for solar eclipses and hence about their observability.

In the main text it was noted that the solar eclipse predictions alternate between Ascending and Descending node and that, in terms of their distance in EYu from the node point, they form an exactly linear ordering. The underlying reason for this is that the positions of the New Moons in EYu within the eclipse year form a complete set of odd numbers from 1 - 445 (with no two being equal), because of their mathematical definition. Since there are 223 odd numbers in this range, it is sufficient to establish that different month numbers map to different positions in EYu in the eclipse year. Suppose m and n are two month numbers in the range 1 - 223. Suppose also that they map to the same position in EYu. By the definition of EYM (using the mathematical language of Excel):

mod((m-1)*38 + 36, 446) = mod((n-1)*38 + 36, 446)

In other words, there is an integer N such that:

(m-1)*38 + 36 = (n-1)*38 + 36 + 446N

(m - n)*19 = 223*N

Since 19 and 223 are prime and m, n are in the range 1 - 223, this can only be true when m = n. So different month numbers map to different positions in EYu in the eclipse year.

Since the DNP is at an even number 66 of EYu, the distances from node point that create glyphs near the DNP form a complete interval of odd numbers in the asymmetric interval defined by the glyph limits (*odd - even = odd*). Similarly, the ANP is at an odd number 289 of EYu, so the distances from node point that create glyphs at the ANP form a complete interval of even numbers in the asymmetric interval defined by the glyph limits (*odd - odd = even*). When put together and written in descending order, a complete interval of integers is created, which alternate between odd and even: in other words between the Ascending and Descending nodes.

It is evident from Figure 7 that there is an asymmetry in the glyphs between North and South. There are twenty glyphs *North* of the node, one *At* the node and seven *South*—nearly three times as many North as South. So the asymmetry noted by Ptolemy [19] certainly preceded him and was understood by the time of the Antikythera Mechanism.

Ptolemy's derivation of the South limit of 8.4° was calculated from Alexandria with a latitude of 31.2° N [19]. It should be possible to reverse Ptolemy's argument, from the 5.4° limit on the Antikythera Mechanism, to derive a latitude for the intended use of the Mechanism—though of course this would imply that Ptolemy's argument was already known when the Mechanism was made. (The designer must have used some method for calculating the South limit from latitude.)

The model is sensitive to changes in some input parameters. It is not very sensitive to changes in the time from New Moon to First Crescent Moon, which can be changed within the range 1.6 - 2.4 EYu (1.2 - 1.9 days). However, even small changes to the positions of the nodes or the glyph limits mean that the model does not work, both for the indexing of the glyphs and the Index Letter Groups—particularly for the L. 29 Group. So the exact positions of the node points at 66 and 289 EYu are critical.

Lunar eclipse inscriptions

As shown in Figure S11, the lunar EPs also alternate between Ascending and Descending—except at the start and finish. The progression in terms of EYu is completely linear—again, except for the first and last entries. The underlying reason for this is that the positions of the Full Moons in EYu within the eclipse year form a complete set of even numbers from 2 - 444 (with no two being equal), because of their mathematical definition (as for the New Moons earlier). The irregularities at the beginning and end of the range are caused by the *consecutive month rule*, which excludes three potential glyphs at the extremes of the limits that define the glyphs in EYM. Without the consecutive month rule, there would be 41 lunar glyphs forming a completely linear progression in terms of distance from node point, with alternation between Ascending and Descending.

Might there also have been *lunar Index Letter Groups* and accompanying inscriptions on the Antikythera Mechanism? In general in the Antikythera Mechanism, dials that include index letters are surrounded by the inscriptions that they index. The front Zodiac Dial includes index letters, which refer to the Parapegma (star calendar) inscriptions round the dial [6]; the Saros Dial includes index letters, which refer to the solar eclipse inscriptions round the dial. The inscriptions on the Metonic dial include much information—*year numbers*, *month names* and *excluded day numbers* [4]—but no index letters. Similarly, the *Olympiad Dial*, which is inside the Metonic Dial, includes the names and year numbers of the *Panhellenic Games* [4], but no index letters. It is also known that the general principles on which these dials were based are described in the Back Cover inscriptions [1], [6]. It seems reasonable to conclude that there were probably no inscriptions relating directly to the Metonic or Olympiad Dials around the Metonic Dial. This is exactly the space that is needed for lunar eclipse inscriptions: it is hard to see the designer resisting the urge to fill this space, since nearly all of the other available spaces on the external plates of the Antikythera Mechanism are covered in inscriptions.

Lunar eclipse directions of obscuration, magnitudes and colour

A persuasive argument in favour of lunar eclipse inscriptions is that the whole scheme works much better for lunar eclipses. It is far more reliable to predict the directions of obscuration of lunar eclipses than solar eclipses. This can be seen in the groups of eclipses shown in Figure S18, where the directions of obscuration relative to the ecliptic (marked by the inclined line) are shared by these groups of eclipses. Figure S12 also shows that the appearance of a lunar eclipse and the trajectory of the Moon relative to the ecliptic are not dependent on the location of the observer, as they are with solar eclipses. The directions of the *shadow vector* will be far more stable in a group of *lunar* eclipses defined by ecliptic latitude—though it must be said the these directions depend to an extent on whether the eclipse is at the Ascending or Descending Node. These directions can be derived from the distance North or South of the node for a lunar eclipse, but are far more uncertain for a solar eclipse, since the directions of obscuration depend critically on the observer's position.

The *magnitude* of a lunar eclipse can also be deduced from its ecliptic latitude and hence from its distance from node point. Again, Figure S18 illustrates why the magnitude of lunar eclipses within an Index Letter Group will broadly share common characteristics.

It is surprising to find eclipse colours in the solar inscriptions, since colours associated with solar eclipses are not often recorded either in ancient or modern astronomy. The opposite is the case for lunar eclipses, whose colours were frequently noted in ancient astronomy. For example, the following, describing the total lunar eclipse of -330 Sep-20, just before Alexander the Great's decisive Battle of Gaugamela (Arbela) [28]:

> *"But about the first watch the Moon in eclipse, hid at first the brilliance of her heavenly body, then all her light was sullied and suffused with the hue of blood."*

There is also a modern colour scale, the Danjon Scale, which describes the colours of lunar eclipses [14]. Lunar eclipse colours were always an established part of astronomy: solar eclipse colours were not.

There are several small fragments of the Antikythera Mechanism, which include text but whose original position has not been determined. An initial examination of these fragments has not identified any that might have been part of lunar eclipse inscriptions round the Metonic Dial. Despite the strength of the arguments, unless further evidence comes to light, the idea of lunar eclipse inscriptions can only remain conjectural—though the arguments are strong.
