# Supplementary material for: Eclipse Prediction on the Ancient Greek Astronomical Calculating Machine Known as the Antikythera Mechanism
Source: PLoS One. 2014 Jul 30;9(7):e103275. doi: 10.1371/journal.pone.0103275 (PMC4116162; doi:10.1371/journal.pone.0103275)
Supplement: Note S4 — Glyph Times. (PDF) [file pone.0103275.s031.pdf]

Note S4: Glyph Times

Time frames

The glyphs, which are presented in full in Figure S13, not only made predictions of a lunar or solar eclipse in a particular month but also what time of day that eclipse might occur. Eclipse times in the glyphs are given as integers on a 12-hour scale using the ancient Greek number system with the alphabet standing for numbers and an additional symbol ς for 6. The times in the glyphs are modified by the Day/Night indicators $\mathrm{H}^{\backslash M}$ and $\mathrm{N}^{\backslash Y}$. These will be translated into a 24-hour scale as follows. The times are assumed to be equinoctial hours [1] and the reference time for the beginning of the day is assumed to be 6.00 am local time. If a lunar eclipse time, n, in a given glyph is preceded by $\mathrm{H}^{\backslash M}$ (*of the day*), the time is taken as t = n. If it is not preceded by $\mathrm{H}^{\backslash M}$, the eclipse is assumed to occur at night and the time is taken as t = n + 12. Similarly, a solar glyph time n is interpreted as t = n if the symbol $\mathrm{N}^{\backslash Y}$ (*of the night*) is absent and t = n + 12 if it is present. "hours mech" refers to the eclipse time in the 24-hour scale described above. In other words, $\mathrm{H}^{\backslash M}$ refers to the period 06:00 hrs - 18:00 hrs (local time); $\mathrm{N}^{\backslash Y}$ refers to the period 18:00 hrs - 06:00 hrs (local time)

The eclipse times given in the NASA/GSFC eclipse maps [14] (Figure S2, Figure S18, Figure S19) are in TD—Terrestrial Dynamic time, which is an absolute time frame. The astronomical reference time frame relevant for this study is Universal Time (UT), since this tied to the rotation of the Earth and hence can be translated into local times. These two time frames differ by Delta T (ΔT), which measures the changes in the Earth's variable rotation: ΔT = TD - UT. In -200, ΔT was 03h33m and in -100 it was 03h14m [14].

Local time depends on longitude, so it is relevant to the intended place of use of the Mechanism: it is determined by local Noon, when the Sun is at its local zenith. There are a number of proposed locations for the intended use of the Mechanism [4], [16]: Sicily—longitude 15°, equivalent to UT + 1.0 hrs; the region of Epiros in north-western Greece—approximate longitude 20°, equivalent to a local time of UT + 1.3 hrs; and Rhodes—longitude 28°, equivalent to UT + 1.9 hrs. So plausible locations for the Mechanism span much of the ancient Greek empire between 15° and 28° longitude. The following equivalents apply to the time frames:

hours mech + 6 = hours local = UT + 1.0 - 1.9 hours, depending on location

Previous publication

A previous publication [4] was deeply pessimistic about the prospects of making sense of the glyph times:

> *"The glyph times are incomplete as their generation remains obscure."*
>
> *"We conclude that the process of generation of glyph times was not sound and may remain obscure."*
>
> *"We have not discovered a rational or plausible basis for the glyph times. We conclude that the generation of the glyph times may not have been well founded and therefore that it may be difficult to discover how it was actually done."*

This research article shows that this pessimism was not justified. A model is developed here that is not exact but is a very close match with the glyph times. One important research stage was to question the previous interpretation of the data and to discover a number of mistakes and probable misreadings.

Analysis of the glyph times

There are 18 published glyphs [4]. The data is of variable quality: some being certain and some uncertain. These are closely examined, since the times are critical to the fit and integrity of the model. It should be borne in mind that the interpretations of the text do not just depend on the single X-ray CT slices that are illustrated here: they depend on the totality of the data, which can only be viewed in

full from the 3D X-ray volumes using 3D X-ray viewing software. Considerable revisions have been made to a previous interpretation [4]: in particular, there have been revisions to the assessments of the glyph times in Glyphs 72, 78, 119, 120, 125, 172. The following are notes on the uncertain eclipse times. The data concerning the glyph times is shown in Figure S13 with close-ups of questioned glyph times in Figure S14 (**A**) - (**I**).

Glyph 13: This is attested by the X-ray CT of Fragment A, which is very indistinct. It probably does not contain $\mathrm{N}^{\backslash \mathrm{Y}}$ and the number is possibly Α or Δ, but very uncertain with current data.

Glyph 72: Figure S14 (**A**)**,** (**B**)**.** The X-ray CT data is from a scan of Fragment A, where the quality is not good. After Η, $\mathrm{N}^{\backslash \mathrm{Y}}$ appears to be very likely. Previously, the text after the $\omega^{\backslash \rho}$ symbol was read as possibly Η [4], but this is probably not correct. It is very likely to be Β.

Glyph 78: Figure S14 (**C**), (**D**). There is a clear Α after the $\omega^{\backslash \rho}$ symbol and some possible marks between these two. Previously, these marks were interpreted as the $\mathrm{N}^{\backslash \mathrm{Y}}$ symbol [4], but this is incorrect. By direct examination of the 3D X-ray CT volume of Fragment F, the first stroke of what was wrongly assumed to be the Ν of $\mathrm{N}^{\backslash \mathrm{Y}}$ can clearly be seen to be the continuation of the right-hand curve of the ω in $\omega^{\backslash \rho}$. In all other glyphs, the modifiers $\mathrm{H}^{\backslash \mathrm{M}}$ and $\mathrm{N}^{\backslash \mathrm{Y}}$ come directly after Σ or Η, so it is very unlikely that $\mathrm{N}^{\backslash \mathrm{Y}}$ would come after $\omega^{\backslash \rho}$, especially since there is plenty of space after Η. So it seems clear that $\mathrm{N}^{\backslash \mathrm{Y}}$ can be ruled out. It could be that there is an Ι (10) after $\omega^{\backslash \rho}$ or that this is simply a random mark. All the established text elements in this glyph are seen as dark lines (low X-ray density), surrounded by a light border (high X-ray density)—presumably where the engraving tool raised the surface metal at the edge of the scribed line. The possible Ι has no such border. In addition, Ι here would be too close to the $\omega^{\backslash \rho}$ symbol. So the conclusion is that the glyph almost certainly reads $\omega^{\backslash \rho}$ Α (Hour 1).

Glyph 119: Figure S14 (**E**)**,** (**F**)**.** $\mathrm{N}^{\backslash \mathrm{Y}}$ is clear. There is an Ι (10) after the $\omega^{\backslash \rho}$ symbol. It looks as if it is just possible that there was a symbol after that. However, close examination of the X-ray CT indicates that this is probably not the case. Though most of the evidence of this glyph is from the accretion layer, there is some persistence of the text into the plate itself but this does not occur for the marks after Ι. The most likely number is $\mathrm{N}^{\backslash \mathrm{Y}}$ Ι (22).

Glyph 120: Σ and the $\omega^{\backslash \rho}$ symbol can just about be made out. $\mathrm{H}^{\backslash \mathrm{M}}$ (day) is definitely included. The symbol after $\omega^{\backslash \rho}$ was previously interpreted in Nature 2008 as Η, but this is highly dubious. Possibly ΙΒ, but very uncertain.

Glyph 125: Figure S14 (**G**), (**H**). This can be seen in the PTM of Fragment A (AK48a) and in the X-ray CT of Fragment A. The diagonal line in (**H**) is a *ring artifact*, which is a defect created in the X-ray CT process. The solar time is certain. The lunar time in Glyph 125 is the character at top-right of (**G**), which has spilled over into Month 126. It was previously reported as Η [4], but the X-ray image in (**H**) strongly suggests that it is probably Β. It looks very similar to the definite Β in Glyph 137 in (**I**), which can be seen on the right of the middle row of characters. The bottom curve of this Β has not been drawn back to the vertical and could easily be mistaken for Η if the top-half were not visible.

Glyph 172: Figure S14 (**J**), (**K**). The lunar eclipse time was previously interpreted as Ε (5) [4], but this is not correct. It is at the top-right of the glyph. The small horizontal top stroke that gave the impression of an Ε is evidently not connected to the rest of the symbol. In any case, it would make an Ε that is too large. This must be a random mark. The symbol looks exactly like all the other instances of **ς** (6), for example in Glyphs 20 and 25. So this glyph is interpreted as almost certain: lunar **ς** (6) and solar ΙΒ (12).

Table S3 summarizes where the glyph times in the data are certain and where uncertain. In some cases, the uncertainties mean that no definite options can be identified; in other cases, there are a limited number of distinct possibilities.

Observations of eclipses

It has been considered that the eclipse times on the Saros dial might have been derived from observations of eclipses and work has been done to find such matches [1]. In any given Saros period of 223 lunar months, most of the solar eclipses would not have been visible from anywhere in the ancient Greek empire. For example, for the "matching" sequence discussed in Note S5, maps of eclipse paths [14] show that, out of the 28 solar eclipses in the Saros period, only 8 were visible from anywhere in the ancient Greek empire. Lunar eclipses would have been visible at night but not during the day. EYM implies that there were 38 predictions of lunar eclipse possibilities and 28 of solar eclipse possibilities. So sufficient observations of eclipse times would have needed to have been made over several Saros periods to have any chance of getting enough data for all the glyphs. A rough estimate might be that a minimum of five Saros periods (90 years) would have been necessary—probably more. If fewer observations were available, it could have been that the data was a mixture of observation and theoretical extrapolation to fill in the gaps—as with the so-called *Saros Canon* from the astronomy of Mesopotamia [8]. This could possibly have been the source of the data. However, this article argues that it is most likely that the eclipse times on the Antikythera Mechanism were derived primarily from an arithmetic model, rather than from observations.

Glyph times and the length of the synodic month

Graphs of the lengths of the synodic month from Full Moon to Full moon and from New Moon to New Moon are shown in Figure S15 (**A**) and (**B**). They reveal the essential features that must be modelled. These graphs also illustrate the essential difficulty of calculating eclipse times, since they depend on the addition of all the previous variable month lengths. Both the lunar and solar anomaly play an important role in determining the length of the month, with lunar anomaly dominating. The graphs exhibit two periodicities. The higher frequency oscillation follows the phase of the lunar anomaly at Full Moon. This produces a beat cycle between the synodic month (with 223 cycles per Saros period) and the anomalistic month (with 239 cycles per Saros period). This beat cycle has a frequency, which is the difference between the two: in other words (239 - 223) = 16 cycles per Saros period, giving a period of 223/16 = 13.94 months. It is known as the *Full Moon Cycle* (FMC) (Note S1) and is equivalent to the cycle that follows the apparent diameter of the Moon, as seen from Earth, as it varies from apogee (small) to perigee (large). The second periodicity is seen in the envelopes of the functions. This variation shows the beat cycle between the *FMC* (16 cycles per Saros) and the *annual cycle* (18.03 cycles per Saros). This has a frequency of (18.03 - 16) = 2.03 cycles per Saros. Notice the complimentary form of the graphs for months FM-FM and months NM-NM: both in terms of lunar anomaly and solar anomaly, they are almost in antiphase for both periodicities.

The eclipse times depend on the addition of the varying lengths of all previous months. So the essential problem is to find a method of calculating month lengths that add up to a match with the eclipse time data in the glyphs.

A flaw in the Antikythera scheme for eclipse times

The eclipse time scheme on the Saros Dial has an inherent flaw. The idea is that the eclipse times repeat every Saros cycle, with a time shift of 8 hours, as shown by the Exeligmos Dial [4]. After three Saros periods—the Exeligmos period of just over 54 years—the eclipse times predicted by the Mechanism should repeat exactly. In reality, this does not happen. One of the reasons is that the Saros period (18.03 years) is not a whole number of years, so the solar anomaly does not repeat after a Saros period and its influence on month lengths (and hence eclipse times) also varies. This produces significantly different eclipse times in successive Saros periods. Table S4 shows that the

accumulative errors can become very large, accumulating to more than five hours over nine Saros periods (162 years) in the lunar example in the table.

Possible models

A number of approaches to modelling the eclipse times have been considered: kinematic and mechanical models, as well as models derived from Babylonian astronomy—both daily increment models and monthly models.

Kinematic & Mechanical models

It is already known that in the Antikythera Mechanism the lunar position in the zodiac is based on the ancient Greek epicyclic theory of lunar motion [1] and the solar position is also likely to have been based on an epicyclic model. So it might seem reasonable that the glyph times were generated by a kinematic model. Kinematic models that implement these epicyclic theories have been tested to see if they match the glyph times, without any good match. In any case, it would have been virtually impossible for the ancient Greeks to have made such mathematical calculations of month lengths. Modern calculations use trigonometry. Whilst trigonometry is believed to have emerged in Hellenistic Greece with the work of Hipparchus and his *table of chords* [29], the modern trigonometry needed to express both lunar and solar positions from the epicyclic theory would have been a near-impossible challenge in ancient Greece. The calculation of syzygies requires the solution of a trigonometric equation, which expresses either the conjunction or opposition of the ecliptic longitudes of Sun and Moon, which has no analytic solution. The *Newton-Raphson* iterative technique was used to determine the times of these syzygies and such methods would not have been available in ancient Greece.

The Antikythera Mechanism itself could have been used to mechanically calculate syzygies by simply moving the Sun and Moon pointers so that they are either in conjunction or opposition. However, the Mechanism is far too inaccurate to have made meaningful calculations of syzygy times [30]. It barely gives accuracies to the nearest day, let alone the nearest hour. It might have been possible to build a much larger machine to make these calculations to reasonable accuracy. The negative theoretical results about the match with the glyph times makes this seem unlikely—though such a mechanical method could have introduced sufficient errors to contradict this argument!

Daily increment models

Since Greek geometric theories of lunar and solar motion do not appear to provide a suitable model for the glyph times, there is little choice but to examine Babylonian arithmetic methods. It is difficult to imagine any other suitable methods that could have been calculated by the ancient Greeks. By the era of the Antikythera Mechanism, the Babylonian techniques had reached great heights of sophistication and could predict lunar eclipse times with an accuracy of an hour [31] and solar times with an accuracy of two hours [32]. It is also known that these Babylonian arithmetic methods persisted, even after the work of Ptolemy in the second century AD, for several hundred years into the Middle Ages [33].

System B methods, which model variable motion with piece-wise linear zigzag functions, will be consdered [9], [11]. Much of the astronomy in the Antikythera Mechanism reflects the descriptions in the first century BC astronomy primer, *Introduction to the Phenomena* by Geminos [12], which describes a way of determining syzygy times, using the Exeligmos Cycle and Babylonian *System B*. This involves the calculation of the daily positions of Sun and Moon, based on a model that calculates the daily incremental positions of these bodies [9], [11]. The increments are calculated from the characteristic zigzag functions of Babylonian System B astronomy. The zigzag functions determine the variable motions that are generated by the lunar and solar anomalies. It is assumed that the motion is constant for each day, where the daily velocity is derived from the zigzag function. The ecliptic longitudes of Sun and Moon are then derived by addition of these daily increments. The hour of syzygy can then be calculated assuming constant motions for the day of syzygy. By the end of the

Saros dial, these models depend on the addition of more than 6,500 daily increments to determine lunar and solar longitudes. So they are very finely dependent on the input parameters for the minima and maxima of the zigzag functions. Small changes in the second sexagesimal place (one part in 3,600) radically alter the generated eclipse times. In exploring such models, it is possible to get a reasonable match for the lunar glyph times or for the solar glyph times, but not for both with the same input parameters. This appears to be true whether the model conforms to the known parameters from Babylonian astronomy or whether slightly different parameters are chosen. Such models cannot be completely excluded, but no match to all the eclipse times has been found with any acceptable accuracy.

The ZigZag Model, ZZM

One other type of model developed by the Babylonians calculated month lengths, based more simply on zigzag functions that depend on the lunar and solar anomalies. These have proved to be more successful for modelling the eclipse times on the Saros Dial. An arithmetic model will be developed that closely conforms with the glyph times. The time of syzygy will be taken as being synonymous with the eclipse time. Though the time of maximum eclipse is usually a few minutes different from the time of syzygy [14], for the purposes of this study, syzygy time is quite accurate enough. An assumption is that the times of eclipses on the Saros Dial are the times of maximum eclipse, though this is by no means certain, since the Babylonian observations usually recorded the times of first contact [8]. Exploration of the idea that the glyph times on the Antikythera Mechanism refer to first contact times has not been successful in getting a better fit between the model and the glyph times.

The model needs to calculate the lengths of each lunar month in a way that closely matches the graphs in Figure S15 (**A**), (**B**) and which add up to match the glyph times. The model is based on Babylonian *System B*. In this system, the length of the lunar month above 29 days is calculated as the addition of a periodic lunar component and a periodic solar component [9], [11]. In the scheme under consideration, the lunar component is dependent on the phase of the lunar anomaly at the end of the mean lunar month and the solar component on the phase of the solar anomaly at the end of the mean lunar month. In the model described here, both of these components are modelled with linear zigzag functions, though the Babylonian data suggest that the solar contribution was more complicated in System B, using second-order difference functions. This is discussed later. Such functions do not appreciably improve the overall match of the model to the glyph times, so the model that is developed here uses linear zigzag functions for both lunar and solar anomaly.

The following notation will be used. FM_n refers to the Full Moon in the nth month of the Saros Dial and NM_n to the nth New Moon. Recall that the months of the Saros Dial start at the first crescent Moon, not astronomical New Moon, so FM_n comes before NM_n in each month. The syzygies in the months before the start of the Saros Dial will be referred to as NM_0, FM_0, NM_{-1}, FM_{-1} etc. *Lunar apogee* will be referred to as L_{apo} and *perigee* as L_{per}; and similarly S_{apo} and S_{per} for the solar equivalents. A graphic of the basic set-up is given in Figure S16 (**A**).

All of the astronomy for the models will be measured from the Full Moon in the first month of the dial, FM_1. The model has a number of input parameters.

Fixed parameters

The period of the *synodic month*, p_{syn} = 29.531 days

The period of the *anomalistic month*, p_{anom} = 27.554 days

The period of the *solar year*, p_y = 365.25 days

These can be calculated from the Metonic and Saros cycles and the customary length of the solar year or they can be derived from Babylonian values. These very small variations make no difference to the times generated by the model.

Parameters tied to the astronomy

The parameters defining the minima and maxima of the zigzag functions, m_l, M_l, m_s, M_s, are tied to the astronomy. They need to be chosen so that the minima, mean and maxima of the lunar month lengths generated by the model conform well to these parameters for real lunar months [14] (Figure S15). For a typical Saros period, these values can be calculated from the NASA/GSFC ephemerides data [14]:

Minimum lunar month length = 29.27 days
Mean lunar month length = 29.53 days
Maximum lunar month length = 29.82 days

In practice, there is some small leeway in the choice of the minima and maxima parameters, m_l, M_l, m_s, M_s, but only within small percentage differences. The Babylonian figures for these parameters are given later.

Free parameters

The free parameters are the phases of the *lunar* and *solar anomalies* at the astronomical reference point, FM_1, as well as the *times* of FM_1 and NM_1. These will be chosen to create the best possible fit between the model and the glyph times.

Lunar anomaly = Number of days $L_{apo} \rightarrow FM_1$
Solar anomaly = Number of days $S_{apo} \rightarrow FM_1$
Time of FM_1
Time of NM_1

In general, the lunar anomaly input will be a number of days from 0 to p_{anom} (27.554 days). However the model is designed so that any number can be entered, since the model evaluates the parameter modulo p_{anom}. For example, negative numbers can be entered and this will be done later to show graphically the value of this parameter that optimizes the model. Similarly, the solar anomaly input will be the number of days from S_{apo} to FM_1—usually a number from 0 to p_y (365.25 days).

Developing ZZM

Figure S16 (**A**) shows the basic setup at the start of the Saros Dial. In ZZM the phases of the lunar and solar anomalies at the end of the month length are calculated on the basis of mean lunar and anomalistic months. The model is the same in principle as the models in *System B* of Babylonian astronomy to determine the lengths of synodic months. In this model, the length of the month is considered to be the sum of two components, G and J, where G depends solely on the lunar anomaly and J on the solar anomaly. G is a linear zigzag function, and some of its defining parameters are preserved [9], [11], [34]. These are expressed in sexagesimal in the customary Babylonian "time units", where each time unit is 1/360th of a day = 1/15 hours = 4 minutes:

m_l = **1, 52; 34, 35** = 112.5668 time units = 7.5046 hours
M_l = **4, 29; 27, 5** = 269.4500 time units = 17.9633 hours

The function J is a correction for the length of the synodic month that takes into account the solar anomaly. The exact details of J are uncertain [9], [11], [13]. It appears to be a function that can be either positive or negative, with a mean value of 0. J seems to have been calculated as a second-order difference function, depending on a first-order linear zigzag difference function H. The preserved records for J and H are incomplete and poorly understood. Second order constant differences produce quadratic functions and so this process generates a set of linked parabolic arcs to model the solar contribution. This procedure does not improve the fit of the model appreciably. Since it is significantly more complicated, ZZM sticks to a linear zigzag function for the solar contribution.

ZZM is based on simple linear zigzag functions for the contribution of both lunar and solar anomaly to the lengths of the synodic month.

The lunar parameters that optimize the model are similar to the Babylonian parameters:

m_l = 7.36 hours (Babylonian 7.50 hours)

M_l = 18.14 hours (Babylonian 17.96 hours)

The solar parameters that optimize this model are:

m_s = - 2.12 hours
M_s = + 2.12 hours (Babylonian maximum of J possibly 2.16 hours [11])

The model is built in an Excel spreadsheet, since this readily emulates the columns of Babylonian System B. (The Babylonian astronomical cuneiform tablets could be regarded as the precursors of modern spreadsheets!) For mathematical formulae, the language of Excel will be used.

Let the phase of the lunar anomaly in days at FM_1 be a_1. Assuming mean motions, for month n the phase of the lunar anomaly at FM_n in units of anomalistic months is:

$a_n = Mod(a_1 + (n-1)*p_{syn}, p_{anom}) / p_{anom}$

This is a number between 0 and 1 anomalistic months. In line with Babylonian System B, the lunar contribution, l_n, is the excess hours over 29 days of the length of the lunar month from F_{n-1} to F_n. It is calculated using a zigzag function as a weighted mean of M_l and m_l, depending on the value of a_n. If the Moon is near apogee, then its apparent velocity is slow and the month is long; and similarly near perigee the month is short.

If a_n<=0.5, then $l_n = ((0.5- a_n)* M_l + a_n* m_l)/0.5$

If 0.5< a_n, then $l_n = ((a_n -0.5)* M_l +(1- a_n)* m_l)/0.5$

In Excel, if X is a condition, then (X) is evaluated as TRUE or FALSE according to whether the condition X is true or false. In calculations, such as (X)*1, (X) evaluates as 1 if (X) = TRUE and 0 if (X) = FALSE. This can be used to avoid the complications of nested IF formulae. So the above expressions for l_n can be expressed as the simple and clear formula:

$l_n = (a_n<=0.5)*((0.5< a_n)*M_l + a_n*m_l)/0.5 + (0.5< a_n)*((a_n -0.5)*M_l +(1- a_n)*m_l)/0.5$

The results of graphing this over a number of months with suitable parameters is shown in Figure S16 (**B**).

Practical computation of this function is very simple. A fixed number d is successively subtracted or added from the running total until the minimum, m_l, or the maximum M_l is reached. At this point the *reflection principle* [9], [11] applies: if subtraction of d would take the function below m_l, then the amount by which it would fall below m_l is added to the minimum. Similarly for additions exceeding M_l. This gives rise to a linear zigzag function. It should be noted that, in general, there is no data point at the minimum or maximum of the function, but all points fall on the zigzag.

For the spreadsheet it might seem simpler to directly express the above process of subtraction in Excel in order to calculate the contribution of the lunar anomaly. However, at each stage a conditional statement would need to be incorporated to decide whether a minimum or maximum had been reached, when the reflection principle would apply and would need to be implemented. This would lead to far more complex formulae than the expression of a simple weighted mean to calculate the lunar contribution, which is exactly equivalent.

The same set of ideas is used for the solar contribution to the excess hours over 29 days of the length of the lunar month. The only difference is that the solar contribution can have either positive or negative values and $m_s = - M_s$. This follows the Babylonian models.

The length of the lunar month is determined by how fast the Moon catches up with the Sun. If the Sun is moving quickly near perigee, the Moon takes longer to catch up and the month is long. Conversely, when the Sun is near apogee, it moves slower and the month is short. So the zigzag function for the solar contribution acts in the opposite way to the lunar contribution: the Sun being near solar apogee shortens the month and being near solar perigee lengthens the month. Let the phase of the solar anomaly in days at FM_n be b_n and the solar contribution to the eclipse time in hours be s_n.

$b_n = Mod(b_1 + (n-1)*p_{syn}, p_y) / p_y$

$s_n = (b_n<=0.5)*((0.5< b_n)*m_s + b_n*M_s)/0.5 + (0.5< b_n)*((b_n -0.5)*m_s +(1- b_n)*M_s)/0.5$

The results of graphing this over a number of months with suitable parameters is shown in Figure S16 (**C**).

The total month length is calculated by simple addition of the lunar and solar contributions, as visualized in Figure S16 (**D**). All of the above applies in a similar way to the months from NM to NM. (The Babylonian technique of adding periodic functions with different periods to approximate a continuously varying function lies at the root of the development of Fourier analysis 2,000 years later.)

The minima and maxima of the zigzag functions are crucial in matching the month lengths of the model to real month lengths. Actual month lengths vs model month lengths are graphed over a Saros period of 223 months in Figure S15 (**C**), (**D**). The graphs of the resulting month lengths are very similar in form to the graphs of actual months—though somewhat more spikey. The parameters m_l, M_l, m_s, M_s determine the large and small maxima and minima of these graphs, as well as the mean value of the computed month lengths. This is why they are referred to as "tied parameters", since their values cannot be altered very much without compromising a reasonable match of the model with actual month lengths. The free parameters, $L_{apo}\rightarrow FM_1$ and $S_{apo}\rightarrow FM_1$ determine the starting phases of the short and long frequency variations of the graph and can be chosen at will. The complete model can now be explored. Figure S17 shows the model match with arbitrary input parameters for the phases of the lunar and solar anomalies at FM_1 and the syzygy times at FM_1 and NM_1. The error graphs show the matches between the model and the glyph times. With these parameters, the match between glyph times and calculated times is hopelessly inaccurate with a total rms error of 6.45 hours. To get a good fit to the glyph times, the input parameters must be optimized.

Optimizing the parameters of the model

Determining the tied parameters

Babylonian parameters are used to set the initial tied parameters—in other words, the parameters that determine the shape of the synodic month graphs (Figure S15 (**C**), (**D**)). A combination of trial-and-error, graphs and Excel VBA macros was then used to optimize all the parameters.

Determining the free parameters

The error between two times will be defined as the *clock distance* between the times. This is the shortest distance round a clock between the two times. For example, the clock distance between 23:00 hrs and 01:00 hrs is 2, not 22.

$$\text{Clock distance}(t_1, t_2) = \min(\text{abs}(t_1 - t_2), \text{abs}(24 - (t_1 - t_2)))$$

If $\mathbf{g} = (g_1, g_2 \ldots g_n)$ is a set of glyph times and $\mathbf{t} = (t_1, t_2 \ldots t_n)$ is a set of corresponding model times:

$$\text{rms error}(\mathbf{g}, \mathbf{t}) = \sqrt{}((\textstyle\sum_{k=1}^{n}(\text{Clock distance}(g_k - t_k))^2)/n)$$

The aim is to determine the free parameters that minimize the rms error between the glyph times and the model times. The model was designed with the expectation that it would be optimized with $L_{apo} = FM_1$, because of the previous proposal that each quadrant of the Saros Dial was synchronized with the Full Moon Cycle [1]. With this assumption, the variation in the rms error due to solar anomaly was then graphed, dependent on the number of days that S_{apo} is before FM_1 (Figure 10 (**B**)). The other free parameters are the times of FM_1 and NM_1. These simply move all the calculated eclipse times up and down the y-axis. They are optimized for each choice of the anomaly parameters. Part of this process of optimization is shown in the main text in Fig. 9. In the graph in (**B**) there is an evident minimum error for both lunar and solar glyph times with S_{apo} at 346 days before FM_1. Taking this value, the rms error for different values of the lunar anomaly at FM_1 can be graphed. In the graph in (**C**) there is an evident minimum error at 0 for both lunar and solar glyph times. The minimum for lunar eclipse times is at nearly exactly the same point as for solar times.

Lunar and solar anomalies

In order to use the model, the lunar and solar anomalies must be observed. The solar anomaly is easy to measure, since it is tied to the annual cycle. The lunar anomaly is much harder. Ancient observations were far too inaccurate to measure lunar anomaly directly, by observing the Moon's variable motion relative to the stars. The ancient Babylonians discovered an astonishing relationship between so-called *horizontal observations* of the Sun and Moon near opposition and the lunar anomaly. So ancient astronomers could keep track of the phase of the lunar anomaly. This Babylonian scheme was uncovered in some remarkable research [35], [36].

The model with optimal parameters

Now the fit of the model to the glyphs can be assessed with optimal parameters. The results are shown in the main text in Figure 10 (**A**). Notice that the input times for FM_1 and for NM_1 have been optimized independently. The reason for this is discussed below. Though this is not a perfect model, it is certainly a good model. It should be remembered that each eclipse time generated by the model represents the addition of all the calculated month lengths after the initial time of FM_1. So the errors in the calculations of month lengths in the model are presumably very small. It might be expected that the fit of the model to the glyph times would work well in the early months but become progressively worse, but this is not the case.

The uncertain glyph times

Lunar

Glyph	*Time options*	*ZZM*	*ZZM rounded*
120	**12?**	**11.34**	**11**

Solar

Glyph	*Time options*	*ZZM*	*ZZM rounded*
13	**1, 4, 13, 16**	**13.95**	**14**

Earlier it was discussed how some of the glyph times are uncertain, with a number of possible options. Here it is shown that ZZM calculates times that are consistent with one of the identified options in the uncertain data. In each case, the calculated time is only one hour different from the best option that fits the model.

Second-order difference models

As mentioned earlier, the System B models appear to have used second-order difference functions for the solar anomaly contribution [9], [11], [13]. Though the exact details of the parameters of the Babylonian model are unclear, there are not many choices about how it might have worked. The first-order zigzag H generates a second-order function J that is phase-shifted relative to the first-order zigzag and whose values are not symmetrical relative to the x-axis. It is fairly easy to fix these problems to produce a "normed" function that works in the way that is wanted.

Some defining parameters for the second-order solar anomaly contribution in sexagesimal are recorded [11], including:

M = variously: Version 1: **32;28,6** Version 2: **32;28** Version 3: **32;28,5,15** time units

= 32.4667 time units (to four places of decimals for all versions) = 2.1644 hours

It is hard to know how to interpret this maximum if J is dependent on another function H, with its own minimum and maximum. It is possible to reconstruct a linear zigzag function H (with suitable parameters that preserve the minimum, mean and maximum of actual month lengths), so that J is a second-order difference function. M is far too large to define the maximum of the first-order zigzag function H that generates the second-order function J. It could well refer to the maximum of the derived second-order function J, which are optimized when M = 2.12 for the lunar times. The details of how second-order difference functions might have worked are not given here, since it does not improve the fit of the model to the glyph times.

Surprisingly, the Babylonians are not known to have used second order zigzag functions for the lunar contribution, despite the fact that it is appreciably larger than the solar contribution. It is a natural

extension of the idea of using second-order differences for the solar contribution. It is not difficult to design a model, using second-order differences for both lunar and solar contributions. This generates much smoother graphs of month lengths than ZZM, which are remarkably like actual month lengths, though it does not improve the overall fit with the glyph times. If the input parameters are optimized for the lunar glyphs alone, then it does give a better result, with an rms error of 1.0 hours.

Discussion of errors

If the glyph times were accurately calculated using an arithmetic model, then this model should give exact results when the final figures are rounded. Since ZZM does not exactly match the glyph times, it cannot be the original arithmetic model or else there were mistakes in the calculations. A number of attempts have been made to eliminate the errors in the model. As discussed, second-order difference models have been tried without appreciable improvement. Another approach has been to emulate on a spreadsheet the rounding of calculations to a fixed number of sexagesimal places—again without success. It may have been that there were other types of rounding errors. These were not in general well understood in antiquity [37]:

> *"In general it must be said that the ancients were little concerned about the influence of rounding off and accumulated errors. Often the errors are of the same order of magnitude as the effect under consideration. Apparently it was only under the influence of modern analysis that we have learned to consider the evaluation of errors as an essential part of numerical methods."*

It does not appear that the error problem lies in the fact that ZZM calculates syzygy times, when first eclipse contact times were intended, since the pattern of errors is not systematic in this way. It may be that the answer lies in the subtle corrections that were often made in Babylonian astronomy. It is difficult to identify patterns in the errors because the calculated eclipse times are the summation of a large and variable number of month lengths—so obscuring errors in calculating individual month lengths. An exact model will hopefully be identified eventually but the basic input parameters—particularly the lunar and solar anomalies—will probably not change. Finding such a model is a challenge for experts in ancient Babylonian astronomy. (I have not yet checked *System A* models.) However, ZZM does give a very good approximation to the glyph times. For example, the lunar time for Glyph 190 is exact, despite the fact that ZZM adds up 189 calculated month lengths from FM_1 to reach this time. ZZM is comparable to the first few terms of the Fourier expression of a function: it incorporates the essential phase inputs, but does not quite result in the detailed accuracy of a perfect match. ZZM's optimizing parameters have already given convincing support to the structural theory that the Saros Dial was synchronized with the Full Moon Cycle. In the next section, the implications for determining *epoch* by combining the parameters of EYM and ZZM are explored.
