# Supplementary material for: Eclipse Prediction on the Ancient Greek Astronomical Calculating Machine Known as the Antikythera Mechanism
Source: PLoS One. 2014 Jul 30;9(7):e103275. doi: 10.1371/journal.pone.0103275 (PMC4116162; doi:10.1371/journal.pone.0103275)
Supplement: Note S5 — Astronomy & Epoch. (PDF) [file pone.0103275.s032.pdf]

Note S5: Astronomy & Epoch

Matching exercises

Over the period 400 BC (-399) to 1 BC (0), there are just under 5,000 lunar months. Using an exhaustive Excel VBA macro, it can be established that there are 113 start months, where the sequence of glyphs on the AM match the actual eclipse record, both in position and type of eclipse [1]. All of these matches depend on the inclusion of penumbral lunar eclipses and none of them match the $H^{\backslash M}$ and $N^{\backslash Y}$ data in the glyphs. It seems that something is wrong. It is also not very promising for determining the epoch of the Mechanism. The justification for this matching attempt and the puzzlement at its failure are based on the idea that the glyphs were determined by observation of actual eclipses or maybe a mixture of observation and theoretical interpolation—such as the *Saros Canon* from Babylonian astronomy [8]. This paper challenges this idea: the design of eclipse prediction on the Antikythera Mechanism can best be explained by underlying arithmetic models, not by observation.

If the same matching exercise is now carried out for the full sequence predicted by EYM, then there are 35 matches. Do these matches imply much about epoch? It is important to change the focus of the matching process, since the EYM sequence is purely arithmetical, not in any way based on observations—so it will not necessarily coincide with actual eclipses in the forthcoming Saros period at the date it was created. There might well be a month in the future when EYM predicts perhaps a solar EP but there is no eclipse in reality or perhaps a penumbral lunar eclipse that did not quite happen because the Moon was just too far from the node. The designer simply would not know of the error in advance. The priority when considering matches should therefore be less concerned with one or two errors of glyph matching and more focussed on whether the astronomy fits the input parameters of EYM and ZZM. Are the Moon's node points in the right place for the matching sequence? Is the phase of the Moon's anomaly correct for FM_1? Do the syzygy times in the first month match actual times? Assuming that the glyphs and the glyph times were generated by mathematical models, the greatest priority is to establish that the input parameters are correct.

Matching EYM & ZZM to the astronomy and epoch

The parameters that synchronize EYM and ZZM with the astronomy give information about epoch. Both EYM and ZZM require astronomical inputs that limit the possible start dates for the Saros Dial. For a possible start date for the Saros Dial that matches EYM with the astronomy, it is first necessary to check that the Sun will be at the Moon's descending node point close to 66 EY-units = 51.28 days after NM_0. (Even if EYM were not correct, the position where the Sun is at the first descending node of the Moon on the Saros Dial is closely determined by the glyphs, as is seen in Figure S9, which shows the division of the Saros Dial into eclipse years.)

All the calculations will assume mean motions. Recall that the Line of Nodes precesses backwards relative to the ecliptic towards the Sun at a rate of 12/223 rotations per year (Note S1). So the Sun's mean motion relative to the nodes is ((235/223)* 360)° per year = 379.4° per year. For the Sun to reach the descending node point 51.28 days after NM_0, the ecliptic longitude of the Sun must be close to ((51.28/365.25)*379.4)° = 53.27° at NM_0. Since this calculation is based on mean motions, start dates will be considered, which are within 5° of this figure. To calculate this constraint, the position of the descending node at New Moons was computed using the *Alcyone* ephemeris software.

The optimized input parameters for ZZM also determine constraints on the start date, since L_{apo} should be at FM_1 and S_{apo} should be 346 days before FM_1.The phase of the lunar anomaly at Full Moon can be calculated using the *Alcyone* software, which can output the geocentric angular diameter of the Moon at Full Moon in arcseconds. This is a measure of the lunar anomaly at FM_1 and from this the percentage diameter of the Moon relative to its minimum diameter can be calculated. This varies between 100% when the Full Moon is at apogee to 114% when the Full Moon is at perigee. For ZZM, the Moon should be close to 100% at FM_1 and a margin of error of 2% will be

allowed. The Alcyone software can also compute the ecliptic longitude of the mean Sun at FM_1. According to the optimized parameters for ZZM, S_{apo} should be 346 days = 341.03° before FM_1. Since the ecliptic longitude of S_{apo} is 65.5° [19], this means that the Sun should have a longitude of mod(65.5° + 341.03°, 360) = 46.53° at FM_1. All of these constraints can be expressed on a spreadsheet, which then determines which dates satisfy the optimized input parameters of EYM and ZZM. It is extremely unlikely that the Mechanism's date is outside the range -250 to 0. Possible start dates that satisfy the parameters of both EYM and ZZM within this date range are tabulated in Table S5, which is a comprehensive list within the chosen time period, providing that EYM and ZZM are essentially correct. Notice that the first five of these are one Saros period apart, as are the last three—though the gap between -168-06-03 and -048-01-01 is not a multiple of the Saros period. The top row of Table S5 displays the parameters that should be satisfied for both EYM and ZZM.

The Saros/Exeligmos scheme was designed with the idea that the eclipse times will repeat every Saros cycle with an 8-hour shift, but this is not correct in practice, as is shown in Table S4. So the possibility arises that the inaccuracies of the eclipse time system on the Saros Dial will reveal the Saros period for which the dial was set up: the epoch of the Saros Dial. The parameter that distinguishes these possible start dates is the longitude of the mean Sun at FM_1. According to ZZM this must be close to 46.53°—so that the phase of the solar anomaly at FM_1 optimizes ZZM. There is only one good match for this parameter: with FM_1 at -204-05-12, the longitude of the mean Sun at FM_1 is 46.75°, which is a remarkably close match. All the other values for this parameter are more than 10° wrong.

The date of -204-05-12 for the first Full Moon of the Saros Dial was identified previously (Evans, J., Carman, C. C. *On the Epoch of the Antikythera Mechanism*, Workshop Presentation, Leiden 2013). It came as a considerable surprise that the methods described in this research article came up with exactly the same date, though the methods used here are entirely different. 205 BC is an unexpectedly early date, though not outside the range defined by the evidence. As discussed in Note S2, current stylistic dating of the inscriptions is consistent with this date. The previous work essentially used matches with actual eclipse times as well as the assumption that the pattern of solar EPs on the Saros Dial conforms to a standard Babylonian 8-7-8-7-8- scheme, which is not supported by this study. The method used here to identify this date relies on optimal input parameters for a model that is not exact. Nonetheless, the fact that both sets of arguments point to the same date for the start of the Saros Dial does suggest that it is a strong candidate epoch. It is not necessarily the date of the Mechanism itself: it could just be that there were astronomical observations available for this date. However, it does seem most likely that the Saros Dial was initially set up for a date that was close to the date of manufacture and launch of the Antikythera Mechanism.

Synchronizing ZZM with the astronomy

In order to use ZZM to predict eclipse times, it needs to be synchronised with the astronomy. Determining the hour of a syzygy is a difficult problem in a culture such as ancient Greece without advanced scientific instruments. Observations of the Full Moon with the naked eye are in general inadequate to determine the hour of syzygy: indeed, Full Moon may occur during the day when the Moon is not visible. The situation is even worse for New Moons, which are not usually visible at all. However, the hour of syzygy *can* be determined when there is an eclipse, since the time of maximum eclipse is close to the time of syzygy. This applies to both Full Moons and New Moons, which become visible at solar eclipses. It appears that the only way that the ancient Greeks could have synchronized a mathematical model with the astronomy is by using eclipse times. FM_1 is never an eclipse on the Saros Dial. So the synchronizing eclipse for the lunar glyphs must have been a lunar eclipse either before the start of the Saros Dial or within the dial itself. (In the less likely case that the synchronizing eclipse is after the Saros Dial start, then the month lengths would need to be extrapolated backwards to determine the previous syzygy times. This is no problem with the *System B* models, since they are generated by simple addition or subtraction.)

Can ZZM dispense with a solar eclipse?

When ZZM was first developed, it was assumed that a good approximation to the time of NM_1 could be obtained by using the model to calculate the month length from FM_1 to FM_2 and then adding half this figure to the time at FM_1 to get the time at NM_1. The assumption was that New Moon would occur half way through the month from Full Moon to Full Moon with a good degree of accuracy. This would minimize the number of independent input parameters by making it unnecessary to have a solar eclipse as well as a lunar eclipse to synchronize the New Moon times for the model: a single lunar eclipse would be sufficient to synchronize the complete mathematical model with the astronomy. This assumption turned out to be wrong. Realizing this explained why it was so hard to develop a consistent model that worked for both lunar and solar glyph times when the astronomy was referenced to FM_1, whereas models where the astronomy was referenced to NM_0 seemed to work much better. A specific example illustrates the problem. There was a Full Moon on -193 Dec-04 20:36 UT and the following New Moon was on -193 Dec-18 20:40 UT. This is just 4 minutes over 14 days. It is easy to establish from the NASA/GSFC ephemerides data [14] that the minimum length of the synodic month is close to 29.2 days. Half of this is 14.6 days = 14 days 14 hours. In fact, the NASA/JPL data show that the time from Full Moon to New Moon varies over a surprising range of 24 hours, from a minimum of 14.0 days to a maximum of 15.0 days. It is not at all accurate to estimate the time of the next New Moon by taking the time of Full Moon and adding half of a mean synodic month. The models that were referenced to NM_0 worked better because of the chance that the length of the time from NM_0 to FM_1 was very close to half a mean synodic month. So ZZM must be synchronized to a lunar eclipse for the Full Moon times and a solar eclipse for the New Moon times.

Synchronising eclipses

To establish synchronizing lunar and solar eclipses, the NASA/JPL ephemerides were examined. In Table S6 (**A**), the syzygies and eclipses are derived from the NASA/GSFC ephemerides for the period 48 months before -204-05-12 [14]. It is not necessary to synchronize with a total eclipse: a good partial eclipse would be fine. To synchronize ZZM it seems natural to choose eclipses that are close to the start of the Saros scale—for example, Lunar -22 months, Solar -23 months. There are three plausible lunar eclipses and only one solar. The eclipse times that give the syzygy times at FM_1 and NM_1 in the mathematical model can be calculated for these possibilities and then compared with the actual eclipse times corresponding to the series starting on -204 May-12. Since the model uses mean months for calculation, it is simple to extrapolate forwards to the calculated time of FM_1. It would be expected that the choice of visible solar eclipses for calibration might be small, but the number of suitable lunar eclipses is also limited. The only two eclipses that work well are Lunar -40 months and Solar -23 months: the errors for other dates are too large. It is not clear why neither of the lunar eclipses at -22 months or -28 months would have been chosen. Table S6 (**B**) shows the errors for various synchronizing eclipses and longitudes. At a local time of UT + 1.3 hours, for example, the errors are: Lunar Eclipse - 0.26 hours (- 16 minutes) and Solar + 0.08 hours (+ 5 minutes). These are excellent figures, considering the poor accuracy of clocks (water clocks) and observations in ancient Greece. It is tempting to infer the intended longitude for the use of the Mechanism, but it is not really justified because of the inaccuracy of observations at the time and the fact that the model is not exact.

The matching sequence from -204-05-12

EYM and ZZM establish that eclipse prediction on the Antikythera Mechanism was almost certainly based on simple arithmetic models, rather than on observations. Though the idea is theoretically well-founded, it does not necessarily mean that it will always give good results in practice, since it is based on mean months. The length of the synodic month varies over nearly 14 hours, so the Moon's anomaly makes a real difference in terms of closeness to node and hence to eclipse possibilities.

Having found a likely start date for the Saros Dial, it is interesting to look at the actual eclipses in the subsequent Saros Dial and to compare them with the Index Letter Groups. Historical details of eclipses are available [14] and are a remarkable resource for historians of astronomy. Details of the

full "matching" sequence together with eclipse maps are given in Figure S18 and Figure S19. It should be noted that there is no lunar eclipse in Month 125 to match the observed lunar glyph in the same month (which poses a problem for determining epoch on the basis of observations). This is the glyph that is farthest North of the node: the Moon was just a bit too far North for an actual eclipse to occur. Otherwise, all the glyphs are matched with corresponding eclipses.

The eclipse data suggest that the conjectural lunar Index Letter Groups do group together eclipses with similar characteristics: for example, for the directions of obscuration and the magnitudes of lunar eclipses. For solar eclipses, it is evident that the Index Letter Groups define eclipses with similar eclipse paths in broad geographic terms: the eclipse paths in the group *Very far North of the node* are generally further north in geographic latitude than those in *Far North of the node*, which are in turn further north than those in *Close North of the node*. The eclipse path is the projection of the Moon onto the Earth, so this relationship would be expected, though it is not entirely simple because of the tilt of the Earth's axis—as discussed in Note S1. If the Moon's distance North of the node is given (*gamma* is fixed), then the Moon's shadow will project at different latitudes onto the Earth according to whether the Earth's north pole is tilted towards or away from the Sun. In other words the relationship between gamma and geographic latitude is not simple.

It is interesting to look at the Moon's North-South distance from the node point in EYu compared with the actual gamma of the "matching" sequences of eclipses shown in Figure S18 and Figure S19. Figure S20 shows the close relationship between the theoretical calculation of *EYu from node point* and the *gamma* of the eclipses in the matching sequence, organized by Index Letter Groups. The matches are clearly not exact but the trend is clear. It is striking that the slope of the regression line is exactly the same for both lunar and solar eclipses. It should be noted that for both lunar and solar the North-South divide is in complete agreement between the EPs in the model and actual eclipses.

The Saros Dial and the Full Moon Cycle

Previously, it was proposed that the Saros Dial was designed so that each quarter turn of the dial coincided with a Full Moon Cycle (FMC) [1]. In addition, a possible scale mark was noted at the 3 o'clock position of the Saros Dial (Figure S21 (**A**), (**B**)) and this reinforced this idea. There are 16 FMCs in the Saros period and the dial has 4 turns—so the idea makes sense in design terms, with each quarter turn representing a Full Moon Cycle. This means that the user of the Mechanism can estimate the apparent angular diameter of the Moon at Full Moon at any month of the dial by noting the angle of the pointer within each quadrant. If the pointer is near the start of a quadrant, then the Moon will be close to apogee at Full Moon and so will appear small; with the pointer in the middle of a quadrant the Moon will appear large at Full Moon; and will return to a small angular diameter as the pointer reaches the end of the quadrant. This has obvious implications for the appearance of lunar eclipses. Though the duration of eclipses is mediated by the angular diameter of the Moon at eclipse, there are many other factors that determine duration and these confound any simple relationship—both for lunar and solar eclipses.

The opposite is the case for New Moons and solar eclipses. If the Moon is at apogee at FM_1, then it will be just past perigee when it reaches NM_1, since half of the anomalistic month is only about one day short of half the synodic month. This means that the apparent diameter of the Moon will be large at New Moon when the pointer is near the beginning or end of each quadrant of the dial and it will be small when the pointer is in the middle of the quadrant. The cycle of the angular diameter of the New Moon could reasonably be called the *New Moon Cycle*, though the New Moon is not visible, except at an eclipse. Figure S21 (**C**) shows Total, Annular and Hybrid eclipses for the "matching" sequence from -204 May-12. This amply bears out the design of the dial, with Total eclipses centred around the cardinal points (marked by the red lines); Annular eclipses centred around the intercardinal points (marked by the blue lines); and Hybrid eclipses between these two regions. The coincidence of each quarter of the Saros Dial with the Full Moon Cycle explains why the dial has four turns. It also explains why there is no glyph in Month 1: the start of the dial was designed so that FM_1 was at L_{apo}, not so that the first month included an eclipse prediction.
